# Deubiquitinase USP7 stabilizes KDM5B and promotes tumor progression and cisplatin resistance in nasopharyngeal carcinoma through the ZBTB16/TOP2A axis

**DOI:** 10.1038/s41418-024-01257-x

**Published:** 2024-01-29

**Authors:** Bin Zhang, Jie Li, Yijun Wang, Xixi Liu, Xiao Yang, Zhiyun Liao, Suke Deng, Yue Deng, Zhiyuan Zhou, Yu Tian, Wenwen Wei, Jingshu Meng, Yan Hu, Chao Wan, Zhanjie Zhang, Fang Huang, Lu Wen, Bian Wu, Yajie Sun, Yan Li, Kunyu Yang

**Affiliations:** 1 Cancer Center, Union Hospital, Tongji Medical College, Huazhong University of Science and Technology, Wuhan, 430022 China; 2 Institute of Radiation Oncology, Union Hospital, Tongji Medical College, Huazhong University of Science and Technology, Wuhan, 430022 China; 3 Hubei Key Laboratory of Precision Radiation Oncology, Wuhan, 430022 China

**Keywords:** Cancer microenvironment, Clinical trial design

## Abstract

Cisplatin-based chemotherapy improves the control of distant metastases in patients with nasopharyngeal carcinoma (NPC); however, around 30% of patients fail treatment due to acquired drug resistance. Epigenetic regulation is known to contribute to cisplatin resistance; nevertheless, the underlying mechanisms remain poorly understood. Here, we showed that lysine-specific demethylase 5B (KDM5B) was overexpressed and correlates with tumor progression and cisplatin resistance in patients with NPC. We also showed that specific inhibition of KDM5B impaired the progression of NPC and reverses cisplatin resistance, both in vitro and in vivo. Moreover, we found that KDM5B inhibited the expression of ZBTB16 by directly reducing H3K4me3 at the ZBTB16 promoter, which subsequently increased the expression of Topoisomerase II- α (TOP2A) to confer cisplatin resistance in NPC. In addition, we showed that the deubiquitinase USP7 was critical for deubiquitinating and stabilizing KDM5B. More importantly, the deletion of USP7 increased sensitivity to cisplatin by disrupting the stability of KDM5B in NPC cells. Therefore, our findings demonstrated that USP7 stabilized KDM5B and promoted cisplatin resistance through the ZBTB16/TOP2A axis, suggesting that targeting KDM5B may be a promising cisplatin-sensitization strategy in the treatment of NPC.

## Introduction

At present, the most effective treatments for Nasopharyngeal carcinoma (NPC) include radiotherapy- and/or radiotherapy combined with adjuvant chemotherapy [1]. Cisplatin-based simultaneous/adjuvant radiotherapy and chemotherapy is considered to be the standard treatment for locally advanced NPC [2, 3]. However, cisplatin resistance is one of the main obstacles in the treatment of locally advanced NPC [4].

Previously, some perspectives about cisplatin resistance have been proposed: these include defective drug accumulation [5], defective homologous recombination [6], homologous recombination-independent mechanisms [7] and pro-survival signaling pathways [8, 9]. In recent years, epigenetic alteration has been shown to be associated with cisplatin resistance in many cancers [10]. Epigenetic mechanisms implied the reversible nature of the drug-tolerant phenotype [11]. Therefore, it is of significant clinical importance to clarify the specific mechanism of cisplatin resistance in NPC through the lens of epigenetics.

KDM5B (lysine-specific demethylase 5B), also known as PLU-1 or JARID1B, is a member of a sub-family of the JMJD family (jmjc-KDMs) that removes methyl groups from H3K4me2/3 [12]. By catalyzing demethylation of H3K4me2/3 to inhibit the expression of multiple genes [13–15], KDM5B regulates many cellular processes, including cell stemness [16], DNA repair [17–19], and cancer immunity [20, 21]. However, the role of KDM5B in cisplatin resistance in patients with NPC remains unclear.

Topoisomerases are enzymes responsible for overcoming topological problems in the process of DNA replication, transcription and repair, and has been identified as a target for antitumor therapy [22]. Topoisomerase II- α (TOP2A) plays an important role in genome structure and chromosome separation [23]. The activation of TOP2A also promotes resistance to platinum-based chemotherapy in cancer [24]. Therefore, a deeper understanding of the regulatory mechanisms of TOP2A may help to elucidate the mechanisms of cisplatin resistance in NPC.

In this study, we demonstrated that KDM5B promoted the progression of NPC and resistance to cisplatin in vivo and in vitro. We also identified a novel KDM5B deubiquitinase, USP7, which can enhance the stability of KDM5B in NPC cells. We also showed that KDM5B inhibited the expression of ZBTB16, a transcriptional repressor of TOP2A, thereby regulating the expression of TOP2A. Finally, we found that the USP7/KDM5B/ZBTB16/TOP2A axis contributed to the sensitivity of NPC cells to cisplatin treatment.

## Material and methods

### Cell lines

Human NPC cell lines HNE1 and CNE2 were obtained from the Sun Yat-sen University Cancer Center (Guangzhou, China) [25], and both HNE1 and CNE2 are cell lines of poorly differentiated squamous cell carcinoma in human NPC. NPC cell lines were maintained in RPMI-1640 medium (Gibco, USA) supplemented with 10% fetal bovine serum (FBS; AC03L055, Shanghai Life-iLab Biotech, China) and 100 U/ml each of penicillin and streptomycin. All cells were placed in the incubator at 37 °C in 5% CO2.

### Quantitative real-time PCR (RT-qPCR) assay

In brief, RNA was extracted by using TRIzol reagent (#R401-01 RNA isolater Total RNA Extraction Reagent, vazyme, Nanjing, China). RT-qPCR was performed by using a reverse transcription kit and PCR kit (#R323-01 HiScript III RT SuperMix for qPCR, #Q111-02 AceQ qPCR SYBR Green Master Mix, vazyme, Nanjing, China) referring to the manufacturer’s instructions. GAPDH served as the reference gene. The primer sequences for RT-qPCR are provided in Supplementary Table S2.

### In vivo tumor growth study

Ethical approval was obtained by the Ethics Committee of Tongji Medical College, Huazhong University of Science and Technology for all animal procedures. BALB/c nude mice (4–5 weeks old) were purchased from Shulaibao Biotech (Wuhan, China). Mice were randomly divided into groups (at least *n* = 5). HNE1 cells (5 × 10^6^) treated in different manners were collected and inoculated subcutaneously into the left dorsal flank of nude mice. Tumor volume was calculated using the formula (L × W^2^)/2.

Additional Materials and Methods can be found in Supplementary Materials.

## Results

### Abnormal KDM5B expression promotes proliferation, invasion, and cisplatin resistance in NPC cells

**Fig. 1:**
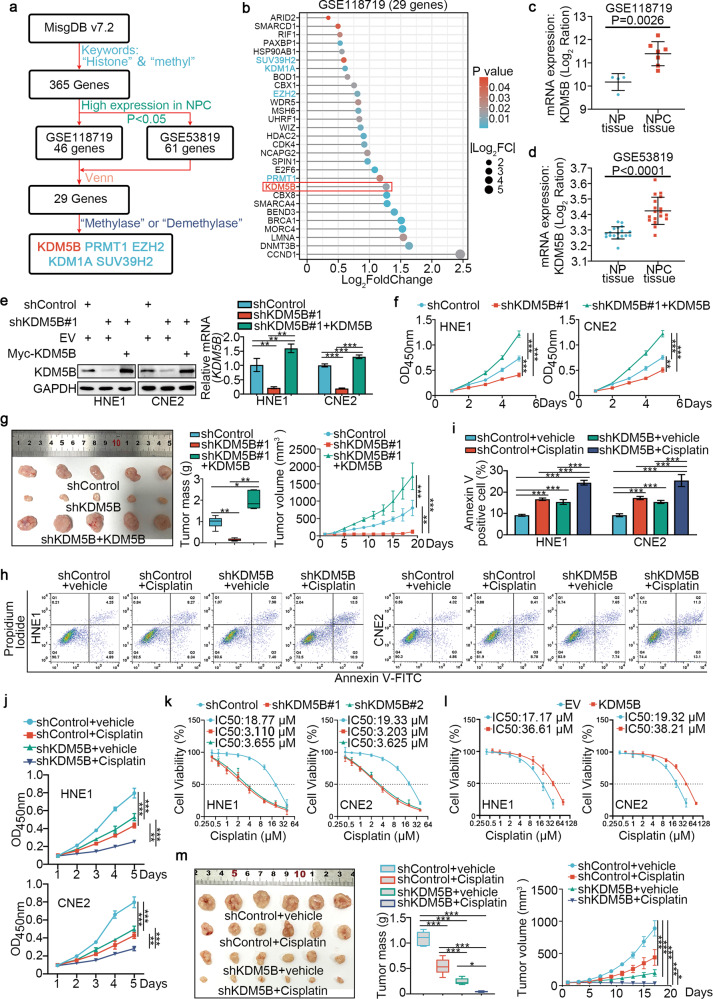
Abnormal KDM5B expression promotes proliferation, invasion and cisplatin resistance of NPC cells. **a** The flow chart of how to identify KDM5B responsible for regulating histone methylation in NPC. **b** The bar chart showing the 29 genes ranking according to Log2FC value. **c**, **d** Differential expression analyses of KDM5B between tumor and normal tissues in GSE118719 (**c**) and GSE53829 (**d**) datasets. **e**, **f** HNE1 and CNE2 cells infected with lentivirus vectors expressing KDM5B specific shRNAs or shKDM5B + KDM5B plasmid, after puromycin selection cells were harvested for Western blotting analysis (**e**), RT-qPCR analysis (**e**), CCK-8 assay (**f**). Statistical significance was determined by one-way ANOVA followed by Tukey’s multiple comparisons test. Data presented as Mean ± SD with three replicates. NS not significant; **P < 0.01; ***P < 0.001. **g** HNE1 cells were transfected with indicated constructs. After puromycin selection, cells were injected subcutaneously into the nude mice for xenografts assay. Tumor volumes were measured every 3 days. Tumors were harvested, photographed, and weighed. Statistical significance was determined by one-way ANOVA followed by Tukey’s multiple comparisons test. Data presented as Mean ± SD with five replicates. *P < 0.05; **P < 0.01; ***P < 0.001. **h**–**j** HNE1 and CNE2 cells were infected with shControl or shKDM5B plasmid for 48 h. then cells were treated with or without cisplatin (4 μg/ml) for another 48 h. Cells were collected for fluorescein isothiocyanate (FITC)/PI flow cytometry (**h**, **i**) and CCK-8 assay (**j**). Statistical significance was determined by one-way ANOVA followed by Tukey’s multiple comparisons test. Data presented as Mean ± SD with three replicates. **P < 0.01; ***P < 0.001. **k** HNE1 and CNE2 cells were infected with shControl or shKDM5B for 72 h. Cells were treated with a serial dose of cisplatin. Then, these cells were collected for CCK-8 assay and subjected to measure the IC_50_ values of cisplatin. **l** HNE1 and CNE2 cells were transfected with EV or myc-KDM5B for 72 h. Cells were treated with a serial dose of cisplatin. Then, these cells were collected for CCK-8 assay and subjected to measure the IC_50_ values of cisplatin. Statistical significance was determined by one-way ANOVA followed by Tukey’s multiple comparisons test. Data presented as Mean ± SD with three replicates. **m** HNE1 cells were transfected with indicated constructs. After puromycin selection, cells were injected subcutaneously into the nude mice for xenografts assay. These mice were treated with or without cisplatin (5 mg·kg^−1^·day^−1^, 10 days). Tumor volumes were measured every 3 days. Tumors were harvested, photographed, and weighed. Statistical significance was determined by one-way ANOVA followed by Tukey’s multiple comparisons test. Data presented as Mean ± SD with six replicates. *P < 0.05; ***P < 0.001.

We conducted a bioinformatics analysis using public databases and identified 365 genes involved in histone methylation-related processes in the MSigDB. Combining transcriptome data from GSE118719 (high expression genes in NPC) and GSE53819 (high expression genes in NPC), we found 5 histone methyl modifier genes, with KDM5B ranking the highest (all *P* < 0.05; Fig. 1a, b). Boxplots (Supplementary Fig. 1a, c) and Principal component analysis (PCA) (Supplementary Fig. 1b, d) were used to visualize the quality control of the GSE118719 and GSE53819 datasets. Further, KDM5B was significantly upregulated in tumor tissues compared with tumor-adjacent tissues in the GSE118719 and GSE53819 datasets (all *P* < 0.001, Fig. 1c, d). To investigate the role of KDM5B in NPC, we silenced KDM5B expression in HNE1 and CNE2 cells (Supplementary Fig. 2a, b). CCK-8 (Cell Counting Kit-8), colony-formation and Transwell assays showed that KDM5B depletion suppressed NPC cell progression in vitro (Supplementary Fig. 2c–e). Moreover, we showed that rescue of KDM5B expression in cells with KDM5B silencing reversed the suppressive effects on cell proliferation that induced by KDM5B knockdown in vitro and in vitro (Fig. 1f, g and Supplementary Fig. 2f, g).

Next, we aimed to investigate whether KDM5B contributed to cisplatin resistance in NPC cells. We demonstrated that KDM5B silencing sensitized NPC cells to cisplatin, whereas KDM5B overexpression in NPC cells increased the half-maximal inhibitory concentration (IC50) for cisplatin (Fig. 1k, l). Moreover, CCK-8 and colony-formation assays showed that KDM5B silencing enhanced the inhibitory effect of cisplatin on NPC cell proliferation (Fig. 1j and Supplementary Fig. 2h). We also performed Annexin-V/propidium iodide (PI) assay to show that KDM5B knockdown plus cisplatin treatment significantly increased apoptosis (Fig. 1h, i). Additionally, the inhibitory effect of cisplatin on NPC tumor growth was significantly increased following KDM5B knockdown (Fig. 1m). Under the treatment of cisplatin, the knockdown KDM5B group further enhanced the activation of Caspase 3 compared with the control group (Supplementary Fig. 2i).

### KDM5B negatively regulates ZBTB16 expression in NPC

**Fig. 2:**
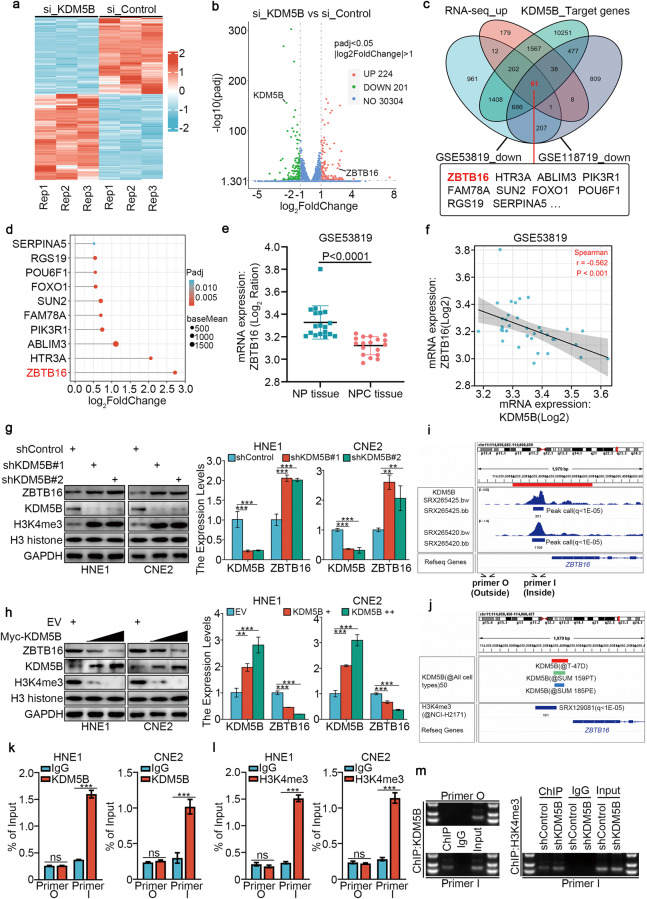
KDM5B negatively regulates ZBTB16 expression in NPC. **a**, **b** HNE1 cells infected by siControl or siKDM5B were harvested for Transcriptome RNA sequencing. Heatmap (**a**) and volcano plot (**b**) were used to show the differential expressed genes. **c** Venn diagrams showing numbers of target genes of KDM5B and downregulated genes in the GSE118719 and GSE53819 databases. **d** The bar chart showing the top of 10 genes ranking according to Log2FC value. **e** Differential expression analyses of ZBTB16 between tumor and normal tissues in GSE53819 databases. **f** ZBTB16 was negatively correlated with KDM5B in GSE53819 databases. **g** HNE1 and CNE2 cells were infected with shControl, shKDM5B #1, or shKDM5B #2 for 72 h. Cells were collected for Western blotting analysis and RT-qPCR analysis. **h** HNE1 and CNE2 cells were infected with indicated plasmids for 72 h. Cells were collected for Western blotting analysis and RT-qPCR analysis. Statistical significance was determined by one-way ANOVA followed by Tukey’s multiple comparisons test. Data presented as Mean ± SD with three replicates. **P < 0.01; ***P < 0.001. **i**, **j** The ChIP-seq of KDM5B on the promoter region of *ZBTB16*. **k**–**m** The ChIP-qPCR of KDM5B on the promoter region of *ZBTB16* in HNE1 and CNE2 cells (**k**, **l**). And the DNA electrophoresis of the products from the ChIP assay (**m**). Statistical significance was determined by two-side Student *t*-test. Data presented as Mean ± SD with three replicates. ***P < 0.001.

To further explore the underlying mechanism by which KDM5B regulates the progression of NPC cells, we applied RNA-seq analysis after silencing KDM5B with siRNA in HNE1 cells (Fig. 2a, b), and we used Western Blotting to confirm the successful silencing of KDM5B (Supplementary Fig. 3a). PCA and boxplots (Supplementary Fig. 3b, c) were used to visualize the quality control of RNA sequencing. ChIP-Atlas (http://chip-atlas.org/) was used to predict the target genes of KDM5B. Next, we combined our RNA-seq datasets (upregulated genes) with the GSE118719 (downregulated genes) and GSE53819 (downregulated genes) databases to screen 61 candidate genes (Fig. 2c). We analyzed these 61 candidate genes using GO enrichment analysis and found no pathway related to cisplatin resistance (Supplementary Fig. 3d). Consequently, we chose the most upregulated gene, ZBTB16 (Fig. 2d), from these 61 genes for further study. Consistently, in the GSE53819 dataset, the expression of ZBTB16 in tumor tissue was significantly downregulated compared with normal tissue (Fig. 2e), and was negatively correlated with KDM5B expression (Fig. 2f). We next assessed whether KDM5B inhibits ZBTB16 expression directly through histone demethylation in the *ZBTB16* promoter region. We found that KDM5B knockdown increased the protein and mRNA levels of ZBTB16 in HNE1 and CNE2 cells (Fig. 2g). Conversely, KDM5B overexpression reduced ZBTB16 mRNA and protein levels in HNE1 and CNE2 cells (Fig. 2h). We also performed an NPC tissue microarray (*n* = 35). The results showed a significant negative correlation between KDM5B and ZBTB16 in samples from patients with NPC (Supplementary Fig. 7a). ChIP-Atlas analysis revealed a KDM5B binding peak in the promoter of *ZBTB16* (Fig. 2i). We also showed that KDM5B repressed the transcription of target genes by removing methyl groups from H3K4me2/3 [12], and H3K4me3-binding sites overlapped with the KDM5B-binding sites in the promoter region of *ZBTB16* (Fig. 2j). ChIP-qPCR analysis revealed the presence of KDM5B and H3K4me3 in the promoter of *ZBTB16* (Fig. 2k, l), and demonstrated that KDM5B knockdown enhanced H3K4me3 binding (Fig. 2m).

### KDM5B promotes progression and cisplatin resistance in NPC cells by directly inhibiting the expression of ZBTB16

**Fig. 3:**
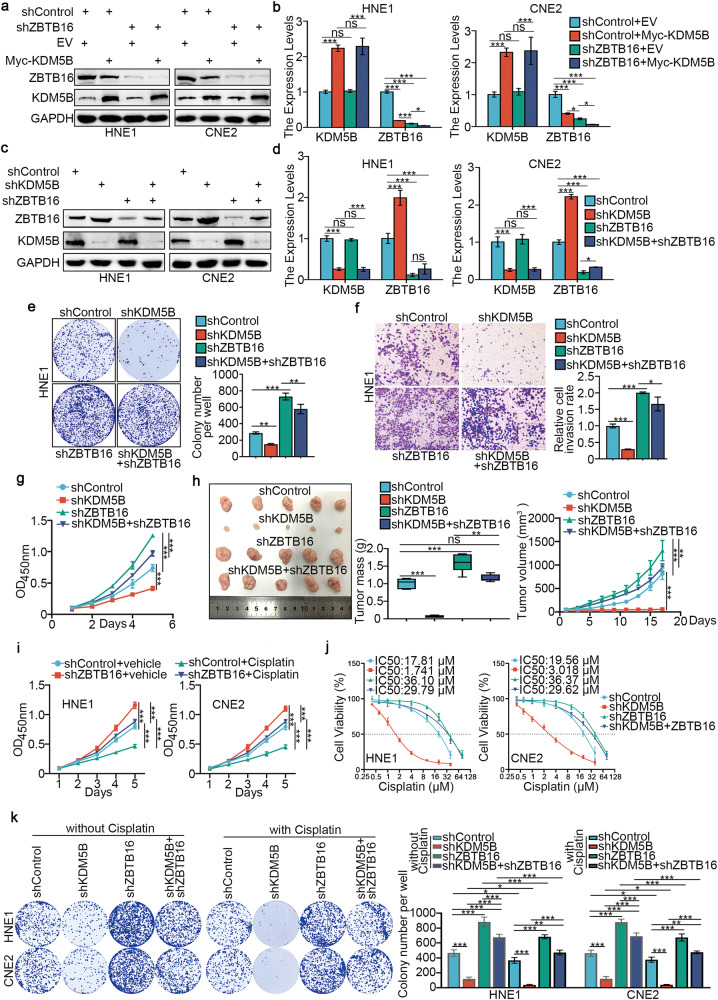
KDM5B promotes the progression and cisplatin resistance of NPC by directly inhibiting the expression of ZBTB16. **a**, **b** HNE1 and CNE2 cells were infected with shControl or shZBTB16 for 48 h. Then, cells were transfected with pcDNA3.1 or myc-KDM5B as indicated. After 24 h, cells were harvested for Western blotting analysis (**a**) and RT-qPCR analysis (**b**). Statistical significance was determined by one-way ANOVA followed by Tukey’s multiple comparisons test. Data presented as Mean ± SD with three replicates. NS not significant; *P < 0.05; ***P < 0.001. **c**–**g** HNE1 and CNE2 cells were infected with indicated shRNAs for 72 h. Cells were collected for Western blotting analysis (**c**), RT-qPCR analysis (**d**), colony-formation assay (**e**, only HNE1 cells) and transwell assay (**f**, only HNE1 cells) and MTS assay (**g**, only HNE1 cells). Statistical significance was determined by one-way ANOVA followed by Tukey’s multiple comparisons test. Data presented as Mean ± SD with three replicates. NS not significant; *P < 0.05; ***P < 0.001. **h** HNE1 cells were infected with indicated shRNAs. After 72 h puromycin selection, cells were harvested and subcutaneously injected into nude mice for xenografts assay. Tumor volumes were measured every 3 days. Tumors were harvested, photographed, and weighed. Statistical significance was determined by one-way ANOVA followed by Tukey’s multiple comparisons test. Data presented as Mean ± SD with five replicates. NS not significant; **P < 0.01; ***P < 0.001. **i** HNE1 and CNE2 cells were infected with shControl or shZBTB16 plasmid for 48 h. then cells were treated with or without cisplatin (4 μg/ml) for another 48 h. Cells were collected for CCK-8 assay. **j** HNE1 and CNE2 cells were infected with indicated shRNAs for 72 h. Cells were treated with a serial dose of cisplatin. Then, these cells were collected for CCK-8 assay and subjected to measure the IC_50_ values of cisplatin. **k** HNE1 and CNE2 cells were infected with indicated shRNAs for 48 h. then cells were treated with or without cisplatin (4 μg/ml) for another 48 h. Cells were collected for colony-formation assay. Statistical significance was determined by one-way ANOVA followed by Tukey’s multiple comparisons test. Data presented as Mean ± SD with three replicates. *P < 0.05; **P < 0.01; ***P < 0.001.

To explore the function of ZBTB16 in NPC, we silenced ZBTB16 expression in HNE1 and CNE2 cells (Supplementary Fig. 4a, b). CCK-8, colony-formation and Transwell assays showed that ZBTB16 depletion promoted NPC cell proliferation, invasion, and migration in vitro (Supplementary Fig. 4c–e). We also showed that overexpression of KDM5B in ZBTB16 ablation cells further reduced the expression of ZBTB16 compared with knockdown of ZBTB16 alone in HNE1 and CNE2 cells (Fig. 3a, b). Co-knockdown of KDM5B and ZBTB16 attenuated both the downregulation effect of ZBTB16 induced by ZBTB16 silencing alone (Fig. 3c, d), and the promotion effect of NPC cells in vitro and in vivo induced by ZBTB16 silencing alone (Fig. 3e–h). Furthermore, ZBTB16 silencing in NPC cells increased IC_50_ values for cisplatin (Supplementary Fig. 4f), while ZBTB16 overexpression rendered NPC cells more sensitive to cisplatin treatment (Supplementary Fig. 4g). CCK-8 assays showed that ZBTB16 silencing decreased the inhibitory effect of cisplatin on NPC cell proliferation (Fig. 3i). Through Annexin-V/PI analysis, we showed that ZBTB16 knockdown plus cisplatin treatment significantly reduced the rate of apoptosis compared with cisplatin alone (Supplementary Fig. 4h). Notably, IC_50_ cytotoxicity assay (Fig. 3j), colony-formation (Fig. 3k) and CCK-8 (Supplementary Fig. 4i) assay all showed that ZBTB16 silencing alone promotes cisplatin resistance in NPC cells, but this effect was weakened by co-knockdown of KDM5B and ZBTB16.

### ZBTB16 negatively regulates TOP2A expression in NPC

**Fig. 4:**
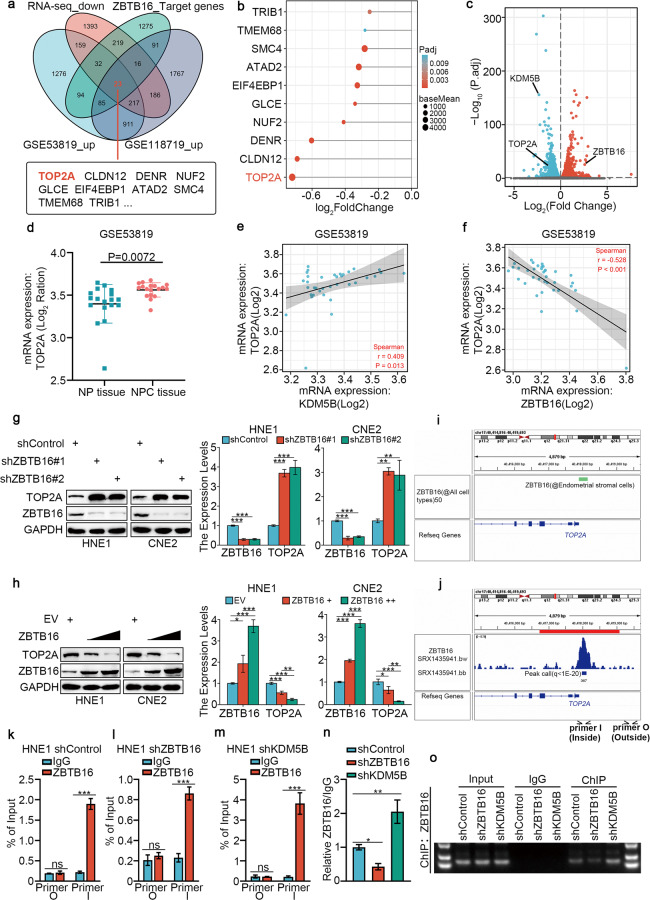
ZBTB16 negatively regulates TOP2A expression in NPC. **a** Venn diagrams showing numbers of target genes of ZBTB16 and upregulated genes in the GSE118719 and GSE53819 databases. **b** The bar chart showing the top of 10 genes ranking according to Log2FC value. **c** volcano plot was used to show the differential expressed genes from RNA-seq. **d** Differential expression analyses of TOP2A between tumor and normal tissues in GSE53819 databases. **e** TOP2A was negatively correlated with ZBTB16 in GSE53819 databases. **f** TOP2A was positively correlated with KDM5B in GSE53819 databases. **g** HNE1 and CNE2 cells were infected with shControl, shZBTB16 #1, or shZBTB16 #2 for 72 h. Cells were collected for Western blotting analysis and RT-qPCR analysis. **h** HNE1 and CNE2 cells were infected with indicated plasmids for 72 h. Cells were collected for Western blotting analysis and RT-qPCR analysis. Statistical significance was determined by one-way ANOVA followed by Tukey’s multiple comparisons test. Data presented as Mean ± SD with three replicates. **P < 0.01; ***P < 0.001. **i**, **j** The ChIP-seq of ZBTB16 on the promoter region of *TOP2A*. **k**–**o** HNE1 cells infected with shControl, shZBTB16, shKDM5B, were harvested for ChIP-qPCR using ZBTB16 antibody (**k**–**m**). The relative quantification of ChIP-qPCR in different groups was presented (**n**). And the DNA electrophoresis of the products from the ChIP assay (**o**). Statistical significance was determined by two-side Student *t*-test. Data presented as Mean ± SD with three replicates. NS not significant; *P < 0.05; **P < 0.01. ***P < 0.001.

According to previous reports, as a transcriptional regulator, ZBTB16 is unlikely to directly regulate cisplatin resistance in NPC. Therefore, we explored downstream effector molecules that might mediate cisplatin resistance. We used ChIP-Atlas to predict the potential downstream target genes of ZBTB16 and combined our RNA-seq datasets (downregulated genes) with the GSE118719 (upregulated genes) and GSE53819 (upregulated genes) databases to screen 23 candidate genes (Fig. 4a), among which TOP2A was the most significantly downregulated after KDM5B silencing (Fig. 4b). We used a volcanic map to depict the regulatory relationship between ZBTB16, TOP2A and KDM5B after KDM5B silencing (Fig. 4c). The GSE53819 dataset showed that the expression of TOP2A in tumor tissue was significantly upregulated compared with normal tissue (Fig. 4d), and was positively correlated with KDM5B (Fig. 4e) and negatively correlated with ZBTB16 (Fig. 4f). Based on the molecular function of TOP2A, we hypothesize that TOP2A plays a crucial role in mediating KDM5B to enhance cisplatin resistance in NPC cells. We detected the accumulation of DNA double-strand breaks (DSBs) induced by cisplatin by γ-H2AX foci assay and western blot. The results showed that TOP2A knockdown (Supplementary Fig. 5a–c) or combined knockdown of KDM5B and TOP2A (Supplementary Fig. 5d–f) prolonged the retention of γ-H2AX foci at the DSB sites after the treatment of cisplatin.

It has been reported that ZBTB16 acts as a transcriptional repressor [26, 27], so we assessed whether ZBTB16 directly inhibits the transcriptional process of *TOP2A*. Our results showed that ZBTB16 knockdown increased the protein and mRNA levels of TOP2A (Fig. 4g). Conversely, ZBTB16 overexpression reduced TOP2A mRNA and protein levels (Fig. 4h). KDM5B Overexpression can downregulate the expression of ZBTB16 and upregulate the expression of TOP2A (Supplementary Fig. 8a). The IHC experiments showed a significant negative correlation between ZBTB16 and TOP2A protein expression in samples from patients with NPC (Supplementary Fig. 7b). Analyses using ChIP-Atlas revealed a ZBTB16 binding peak in the promoter of TOP2A (Fig. 4i, j). Subsequent ChIP-qPCR analysis indicated that ZBTB16 bound to the promoter region of TOP2A (Fig. 4k) and that knockdown of ZBTB16 attenuated this binding (Fig. 4l, n, o) but knockdown of KDM5B enhanced this binding (Fig. 4m–o).

### KDM5B promotes progression and cisplatin resistance in NPC cells through ZBTB16-mediated transcriptional regulation of TOP2A

**Fig. 5:**
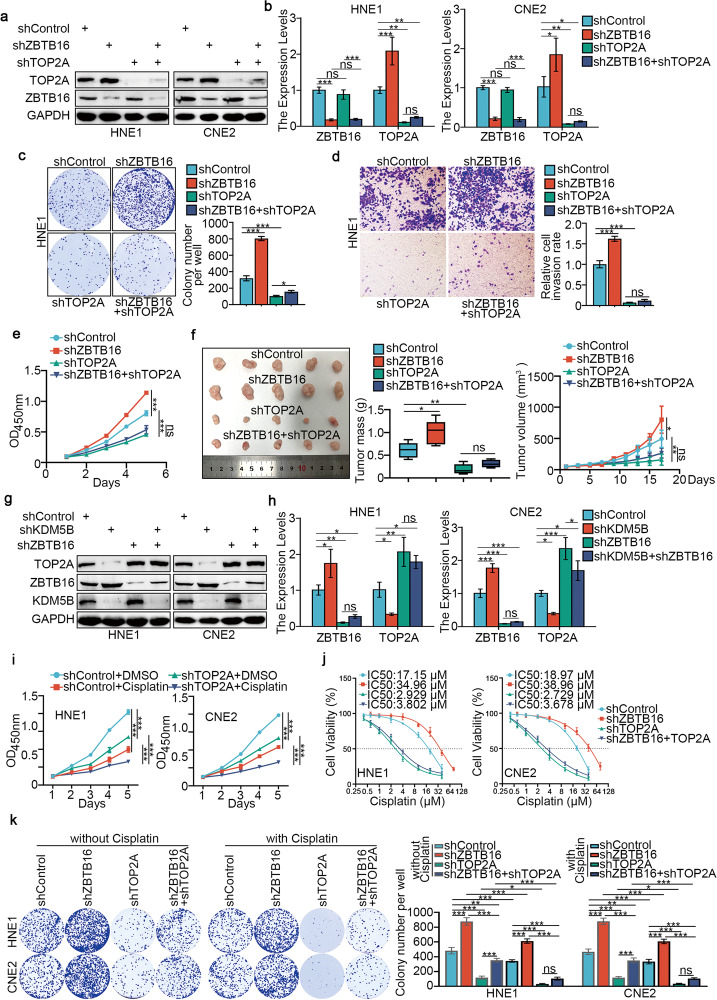
KDM5B promotes progression and cisplatin resistance in NPC cells through ZBTB16-mediated transcriptional inhibition of TOP2A. **a**–**e** HNE1 and CNE2 cells were infected with indicated shZBTB16 or shTOP2A for 72 h. Cells were collected for Western blotting analysis (**a**), RT-qPCR analysis (**b**), colony-formation assay (**c**, only HNE1 cells) and transwell assay (**d**, only HNE1 cells) and MTS assay (**e**, HNE1 cells). Statistical significance was determined by one-way ANOVA followed by Tukey’s multiple comparisons test. Data presented as Mean ± SD with three replicates. NS not significant; *P < 0.05; **P < 0.01. ***P < 0.001. **f** HNE1 cells were infected with indicated shRNAs. After 72 h puromycin selection, cells were harvested and subcutaneously injected into nude mice for xenografts assay. Tumor volumes were measured every 3 days. Tumors were harvested, photographed, and weighed. Statistical significance was determined by one-way ANOVA followed by Tukey’s multiple comparisons test. Data presented as Mean ± SD with five replicates. NS not significant; *P < 0.05; **P < 0.01. **g**, **h** HNE1 and CNE2 cells were infected with indicated shKDM5B or shZBTB16 for 72 h. Cells were collected for Western blotting analysis (**g**), RT-qPCR analysis (**h**). Statistical significance was determined by one-way ANOVA followed by Tukey’s multiple comparisons test. Data presented as Mean ± SD with three replicates. NS not significant; *P < 0.05; **P < 0.01. ***P < 0.001. **i** HNE1 and CNE2 cells were infected with shControl or shTOP2A plasmid for 48 h. then cells were treated with or without cisplatin (4 μg/ml) for another 48 h. Cells were collected for CCK-8 assay. **j** HNE1 and CNE2 cells were infected with indicated shRNAs for 72 h. Cells were treated with a serial dose of cisplatin. Then, these cells were collected for CCK-8 assay and subjected to measure the IC_50_ values of cisplatin. **k** HNE1 and CNE2 cells were infected with indicated shRNAs for 48 h. then cells were treated with or without cisplatin (4 μg/ml) for another 48 h. Cells were collected for colony-formation assay. Statistical significance was determined by one-way ANOVA followed by Tukey’s multiple comparisons test. Data presented as Mean ± SD with three replicates. NS not significant; *P < 0.05; **P < 0.01; ***P < 0.001.

To explore the function of TOP2A in NPC, we silenced TOP2A in HNE1 and CNE2 cells (Supplementary Fig. 6a, b). CCK-8, colony-formation and Transwell assays showed that TOP2A depletion inhibited NPC cell proliferation, invasion and migration in vitro (Supplementary Fig. 6c–e). We also showed that co-knockdown of ZBTB16 and TOP2A attenuated the downregulation effect of TOP2A induced by TOP2A silencing alone (Fig. 5a, b), and the inhibitory effect of NPC cells in vitro and in vivo induced by TOP2A silencing alone (Fig. 5c–f). Moreover, co-knockdown of ZBTB16 and KDM5B reduced the upregulation effect of TOP2A by ZBTB16 silencing alone (Fig. 5g, h). These data suggested that ZBTB16 is associated with downregulation of TOP2A and subsequent inhibition of tumor growth in NPC. Furthermore, TOP2A silencing in NPC cells reduced the IC_50_ values for cisplatin (Supplementary Fig. 6f), while TOP2A overexpression made NPC cells more resistant to cisplatin (Supplementary Fig. 6g), and CCK-8 assays showed that TOP2A silencing enhanced the effect of cisplatin on inhibition of NPC cell proliferation (Fig. 5i). Through Annexin-V/PI analysis, we also showed that TOP2A knockdown plus cisplatin treatment significantly increased the rate of apoptosis compared with cisplatin alone (Supplementary Fig. 6h). Importantly, IC_50_ cytotoxicity assay (Fig. 5j), colony-formation (Fig. 5k) and CCK-8 (Supplementary Fig. 6i) assays all showed that TOP2A silencing alone promoted the sensitivity of NPC cells to cisplatin, but this effect was weakened by co-knockdown of ZBTB16 and TOP2A. In order to further confirm that TOP2A was the crucial downstream of KDM5B, we re-analyzed the IHC data from the tissue microarray stained KDM5B and TOP2A, the results showed a significant positive correlation between KDM5B and TOP2A protein expression (Supplementary Fig. 7c). We knocked down TOP2A expression while suppressing the expression of KDM5B. The results showed that TOP2A protein and mRNA expression decreased, which was also observed when TOP2A alone was silenced (Supplementary Fig. 8c–f). Similarly, overexpression of KDM5B after TOP2A knockdown also resulted in a slight increase in TOP2A protein and mRNA expression (Supplementary Fig. 8g, h). CCK-8 assays showed that KDM5B inhibition enhanced the inhibitory effect of cisplatin on NPC cell proliferation (Supplementary Fig. 9a). Moreover, in *vitro* (Supplementary Fig. 9b–g) assays showed that KDM5B inhibitor AS-8351 enhanced the antitumor effect of cisplatin in NPC, and this process was enhanced after knockdown of TOP2A.

### USP7 promoted the stability of the KDM5B protein in NPC by deubiquitinating KDM5B

**Fig. 6:**
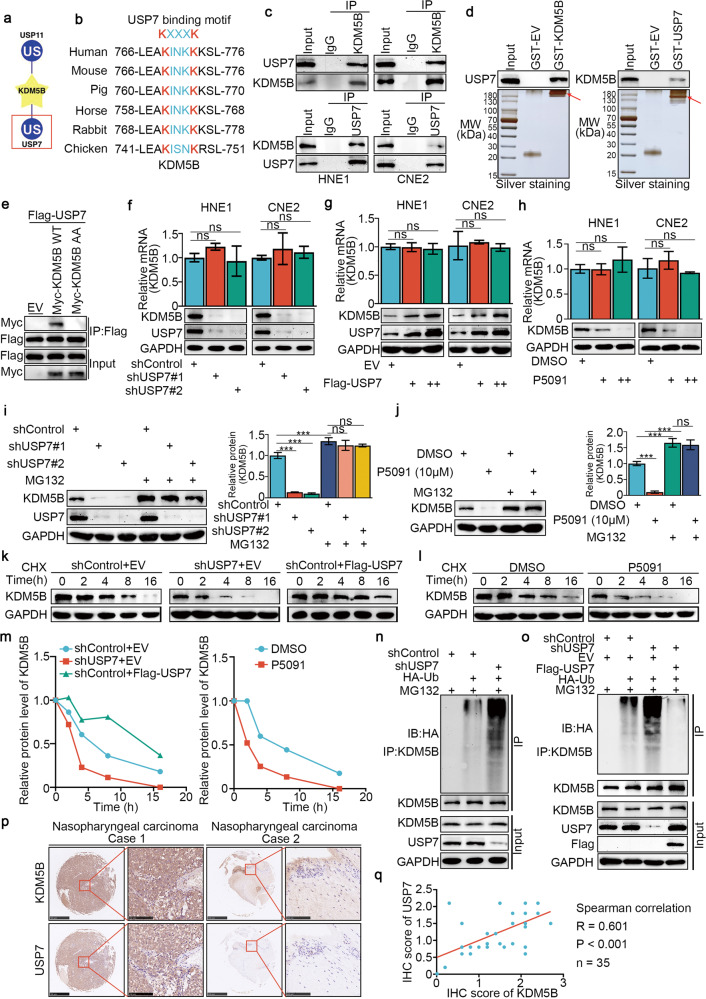
USP7 promoted the stability of the KDM5B protein in NPC by deubiquitinating KDM5B. **a** UbiBrowser showed deubiquitinationases (DUBs) that might interact with KDM5B. **b** A schematic diagram depicted that the USP7 deubiquitination consensus motif of KDM5B. **c** HNE1 and CNE2 cells were harvested and immunoprecipitated with IgG and USP7 or KDM5B antibodies. **d** Western blot analysis for USP7 and KDM5B in HNE1 cells after GST, GST-KDM5B or GST-CBX7 pulldown. The bottom panel shows the Silver staining of GST, GST-KDM5B or GST-CBX7 protein input. **e** HEK293T cells were transfected with the indicated plasmids and harvested 48 h after transfection. Co-IP experiments were performed and blotted with the indicated antibodies. **f**, **g** HNE1 and CNE2 cell lines were infected with constructed plasmids (shUSP7 #1, shUSP7 #2, Flag-USP7). After infecting 48 h and 72 h, all cells were harvested for RT-qPCR and Western Blotting analysis. All data were showed as Means ± SD (*n* = 3). Ns not significant. **h** HNE1 and CNE2 cell lines were treated with or without USP7 inhibitors P5091 (10 μM) for 48 h. Then cells were harvested for RT-qPCR and Western Blotting analysis. All data were showed as Means ± SD (*n* = 3). Ns not significant. **i**, **j** HNE1 and CNE2 cell lines were infected with constructed plasmids (shUSP7 #1, shUSP7 #2). After infecting 48 h and 72 h, the corresponding groups were treated with MG132 for another 4 h. All cells were harvested for Western Blotting analysis. **k** HNE1 cells were infected with indicated plasmids (shUSP7 and Flag-USP7). After infecting 72 h, cells were treated with Cycloheximide (CHX) and all cells were collected for Western Blotting analysis at different time points. **l** HNE1 cells were treated with or without USP7 inhibitors P5091 (10 μM) for 48 h. Cells were treated with MG132 for another 4 h. Then all cells were collected for Western Blotting analysis. **m** Statistical line chart of half-life of KDM5B protein. **n** HNE1 cells were infected with indicated plasmids (shUSP7, HA- Ub). After 24 h, cells were treated with MG132 for 4 h. Then all cells were collected for Western Blotting analysis. **o** HNE1 cells were treated with indicated plasmids (shUSP7, Flag-USP7, HA-Ub) for 24 h. Then cells were treated with MG132 for 4 h. All cells were collected for Western Blotting analysis. **p**, **q** The tissue microarray of NPC was stained with USP7 and KDM5B, respectively (*n* = 35). The typical IHC images stained with USP7 and KDM5B were shown in panel (**p**). The size of the scale bar on microscopy images as indicated in the figure. The correlation of these two proteins was shown in panel (**q**). Spearman correlation was used to determine statistical significance, *P* < 0.001.

We next analyzed the regulation of KDM5B in NPC. UbiBrowser (http://ubibrowser.bio-it.cn/) indicated a close association between the deubiquitinases (DUBs) USP11 and USP7 with KDM5B (Fig. 6a). Coincidentally, upon analyzing the amino acid sequence of KDM5B, we discovered a unique overlap between the USP7 binding motif and the conservative region of KDM5B (Fig. 6b). A coimmunoprecipitation (co-IP) assay showed that endogenously expressed KDM5B interacted with USP7 in both HNE1 and CNE2 cells (Fig. 6c). Consistent with this result, a GST pulldown assay demonstrated that there was an interaction between KDM5B and USP7 in vitro (Fig. 6d). To further investigate whether this binding motif is associated with USP7, we generated a KDM5B mutant, termed KDM5B-AA, with Lys769 and Lys773 residues all mutated to alanine. As shown that (Fig. 6e), unlike wild-type KDM5B (KDM5B-WT), the KDM5B-AA mutant failed to associate with USP7. We also found that KDM5B protein expression decreased or increased after knockdown or overexpression of USP7, respectively, while KDM5B mRNA remained constant (Fig. 6f, g). Furthermore, the changes in KDM5B observed after treatment with the USP7 inhibitor P5091 were consistent with observations following USP7 knockdown (Fig. 6h). We also found that USP7 knockdown using shUSP7 or P5091 could reduce the KDM5B protein expression, which was inhibited by the proteasome inhibitor MG132 (Fig. 6i, j). Moreover, the protein half-life of KDM5B was significantly decreased when USP7 was knocked down or inhibited, while overexpression of USP7 showed the opposite effect (Fig. 6k–m). We found that knocking down or inhibiting USP7 increased the polyubiquitination of KDM5B, and that overexpressing USP7 decreased the polyubiquitination of KDM5B in NPC cells (Fig. 6n, o). To analyze the protein expression of KDM5B and USP7 in tissue, the IHC experiments showed a significant positive correlation between KDM5B and USP7 protein expression in samples from patients with NPC (Fig. 6p, q).

### The USP7/KDM5B/ZBTB16/TOP2A axis modulates cisplatin resistance in NPC cells

**Fig. 7:**
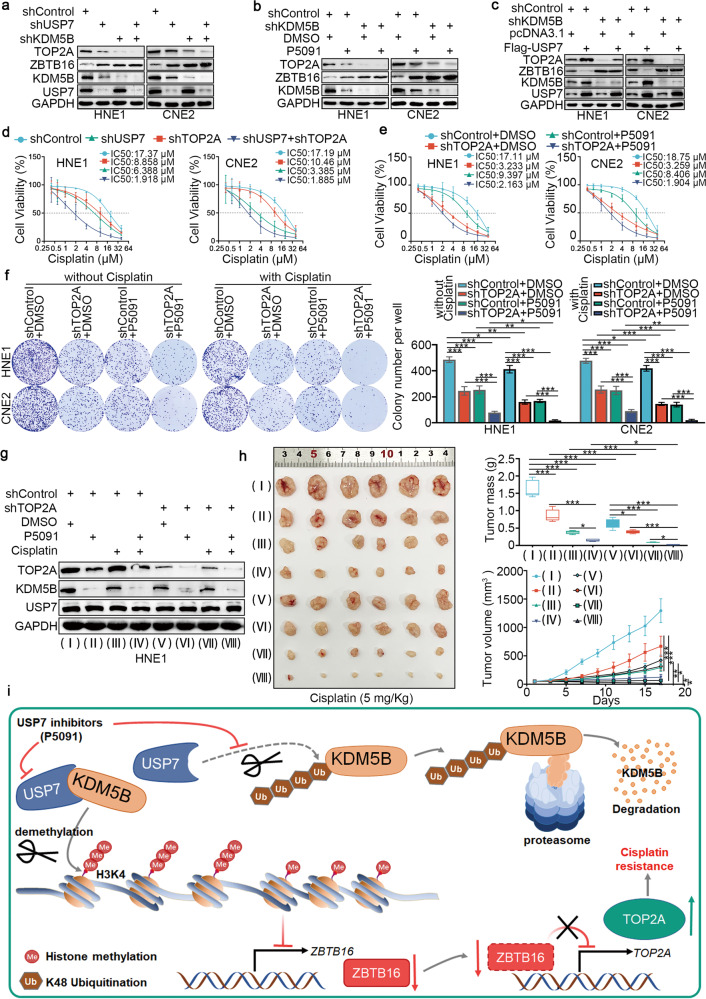
The USP7/KDM5B/ZBTB16/TOP2A axis modulates cisplatin resistance in NPC cells. **a**–**c** HNE1 and CNE2 cell lines were infected with constructed plasmids (shUSP7, shKDM5B, Flag-USP7). Cells were treated with USP7 inhibitors P5091 (10 μM) in (**b**). After infecting or treating 72 h, all cells were harvested for Western Blotting analysis. **d** HNE1 and CNE2 cells were infected with indicated shRNAs for 72 h. Cells were treated with a serial dose of cisplatin. Then, these cells were collected for CCK-8 assay and subjected to measure the IC_50_ values of cisplatin. **e** HNE1 and CNE2 cells were infected with shControl or shTOP2A plasmid for 48 h. Then cells were treated with USP7 inhibitors P5091 (10 μM) in a serial dose of cisplatin. Then, these cells were collected for CCK-8 assay and subjected to measure the IC_50_ values of cisplatin. **f** HNE1 and CNE2 cells were infected with shControl or shTOP2A plasmid for 48 h. Then cells were treated with USP7 inhibitors P5091 (10 μM) for colony-formation assay in the presence or absence of cisplatin. Statistical significance was determined by one-way ANOVA followed by Tukey’s multiple comparisons test. Data presented as Mean ± SD with three replicates. *P < 0.05; **P < 0.01; ***P < 0.001. **g**, **h** HNE1 cells were infected with indicated shRNAs for 72 h. After puromycin selection, cells were harvested for western blot analysis (**g**) and subcutaneously injected into the nude mice. The western blot analysis was repeated for three replicates. The mice were treated with or without cisplatin (5 mg·kg^−1^·day^−1^, 10 days) or USP7 inhibitors (10 mg·kg^−1^, twice a week). Representative tumor images, tumor weights, and tumor growth curves are shown. Data are shown as mean ± SD (*n* = 6). Statistical analyses were performed with one‐way ANOVA followed by Tukey’s multiple comparisons tests. Ns not significant; *P < 0.05; ***P < 0.001. The ruler on the top of the representative tumor images on panel **h** was used to indicate the specific size of tumors. **i** The schematic diagram for USP7 stabilizing KDM5B to inhibit the expression of ZBTB16, which subsequently increased the expression of TOP2A and promoted NPC cell proliferation and resistance to cisplatin.

After inhibiting or overexpressing USP7, the expression of TOP2A decreased or increased, respectively (Fig. 7a–c). This raised the question of whether USP7 regulated cisplatin resistance in NPC through the KDM5B/ZBTB16/TOP2A axis. To test this hypothesis, we reduced KDM5B expression while suppressing USP7 expression. The results demonstrated a decrease in TOP2A protein expression, which was also observed when KDM5B alone was silenced (Fig. 7a, b). Similarly, the overexpression of USP7 following KDM5B knockdown resulted in a slight increase in TOP2A protein expression (Fig. 7c). Notably, IC_50_ cytotoxicity assays (Fig. 7d) and colony-formation assays (Supplementary Fig. 10a, b) all showed that TOP2A silencing alone increased the sensitivity of NPC cells to cisplatin treatment, but this effect was enhanced by co-knockdown of USP7 and TOP2A. Consistently, in vitro (Fig. 7e, f and Supplementary Fig. 10c–e) and in vivo (Fig. 7g, h) assays showed that USP7 inhibitor P5091 enhanced the antitumor effect of cisplatin in NPC, and this process was enhanced after knockdown of TOP2A. In conclusion, USP7 stabilized KDM5B to inhibit the expression of ZBTB16 by demethylating H3K4 of histone, which subsequently increased the expression of TOP2A and promoted NPC cell proliferation and resistance to cisplatin (Fig. 7i).

## Discussion

Global changes in the epigenetic landscape have been recognized as a hallmark of cancer [28]. In the process of tumorigenesis and progression, DNA methylation and histone post-translational modification regulate complex gene expression networks, which affect tumor growth, metastasis and drug response [29, 30]. Mechanically, epigenetic regulation plays an important role in the regulation of chromatin structure and gene expression. In particular, chromatin modification, which is responsible for chromatin remodeling and specific gene transcription, is strictly controlled by different types of epigenetic modifiers through histone post-translational modifications, including acetylation, methylation and ubiquitin [31].

In recent years, the functions of epigenetic modifiers in platinum sensitivity have begun to emerge [32]. A number of chromatin modifiers that control acetylation and methylation are critically involved in the transcriptional and posttranscriptional regulation of platinum resistance-related genes at multiple levels. For instance, the helicase CHD4, a component of the nucleosome remodeling deacetylase (NuRD) complex, plays a role in promoting cisplatin resistance. It achieves this by suppressing the P21 (also known as CDKN1A) promoter, which leads to a decrease in p21 expression [33]. Furthermore, histone deacetylation and chromatin compaction have been proposed as potential factors contributing to cisplatin resistance in head and neck squamous cell carcinoma, possibly mediated through the nuclear factor-κB (NF-κB) pathway [34]. Moreover, enhancer of zeste homolog 2 (EZH2), a specific H3K27 methyltransferase, has been found to contribute to acquired cisplatin resistance in ovarian cancer cell lines in vitro and grown as xenografts [35]. However, the function of histone modifications in the regulation of the sensitivity of NPC to cisplatin is poorly understood.

Histone methylation is a complex modification that regulates transcription and chromatin dynamics [36–38]. Methylation of H3K4 is an open chromatin mark associated with active gene transcription [39]. In past decades, several demethylase such as KDM5A/B/C/D have been discovered to specifically remove methyl groups from H3K4me2/3 [40–43]. Previous studies have shown that KDM5B can impact chromatin stability and promote drug resistance through chromatin remodeling [44, 45]. For instance, KDM5B demethylates H3K4 to recruit XRCC1 and promote cisplatin resistance in gastric cancer [44]. However, prior to our study, it was unknown whether KDM5B could affect the sensitivity of NPC to cisplatin through transcriptional regulation. Our bioinformatics analysis showed that KDM5B could significantly upregulate the expression of TOP2A. We also showed that KDM5B upregulated the expression of TOP2A by inhibiting the expression of ZBTB16 through demethylation of the transcriptional initiation histone H3K4, and that ZBTB16, as a transcriptional repressor, was directly involved in the transcriptional inhibition of TOP2A. As such, our data suggests that KDM5B may regulate the sensitivity of NPC to cisplatin in a TOP2A-dependent manner in vivo and in vitro.

TOP2A leads to the release of the DNA superhelix by catalyzing the transient breaking and rejoining of two strands of duplex DNA, thus altering the topology of DNA [46–49]. In cancer treatment, TOP2A is the main target of Adriamycin (Adm), etoposide and other chemotherapeutic drugs [50, 51]. Anthracyclines, for example, can be embedded in the site where TOP2A binds to DNA, killing tumor cells by preventing broken DNA from reconnecting. Interestingly, it has been reported that TOP2A reduces DNA damage caused by the binding of platinum drugs to DNA, which can lead to drug resistance by activating nucleotide excision repair [52]. This suggests that TOP2A is a key mediator of cisplatin resistance in NPC cells. Although it has been reported that miR-125b-5p can regulate the expression of TOP2A [53], little is known about the regulation of TOP2A expression. Importantly, the complex regulatory mechanisms underlying the activity of TOP2A require further study.

Next, we showed that USP7 bound to KDM5B to stabilize KDM5B and promoted both the progression of NPC and the development of cisplatin resistance, which were both dependent on TOP2A. We further showed that the USP7 inhibitor, P5091, enhanced the sensitivity of NPC cells to cisplatin by inducing KDM5B degradation, which provides a potential therapeutic strategy for inhibiting the KDM5B/ZBTB16/TOP2A axis in NPC.

## Conclusions

In summary, we showed that increased KDM5B expression led to enhanced growth, invasion, and cisplatin resistance in NPC cells. Additionally, we discovered that KDM5B indirectly elevated TOP2A expression by suppressing ZBTB16 expression. Moreover, we established that USP7 interacted with KDM5B, preventing its degradation. Therefore, inhibiting KDM5B in combination with cisplatin treatment may yield more potent antitumor effects compared to cisplatin treatment alone in NPC patients.

### Supplementary information

Supplementary_information

Original Data File

aj-checklist

## Data Availability

The RNA-seq data is provided in Supplementary Table S4. The datasets used and/or analyzed during the current study are available from the corresponding authors (sunyajie@hust.edu.cn) on reasonable request.
