# Supplementary material for: Deubiquitinase USP7 stabilizes KDM5B and promotes tumor progression and cisplatin resistance in nasopharyngeal carcinoma through the ZBTB16/TOP2A axis
Source: Cell Death Differ. 2024 Jan 29;31(3):309–21. doi: 10.1038/s41418-024-01257-x (PMC10923876; doi:10.1038/s41418-024-01257-x)
Supplement: Supplementary file 1 — Supplementary_information [file 41418_2024_1257_MOESM1_ESM.pdf]

Deubiquitinase USP7 stabilizes KDM5B and promotes tumor progression and cisplatin resistance in nasopharyngeal carcinoma through the ZBTB16/TOP2A axis

Bin Zhang, Jie Li, Yijun Wang, Xixi Liu, Xiao Yang, Zhiyun Liao, Suke Deng, Yue Deng, Zhiyuan Zhou, Yu Tian, Wenwen Wei, Jingshu Meng, Yan Hu, Chao Wan, Zhanjie Zhang, Fang Huang, Lu Wen, Bian Wu, Yajie Sun, Yan Li, Kunyu Yang

Supplementary Material and Methods

Antibodies and reagents

KDM5B (#A14104, ABclonal, 1:500 dilution), USP7 (#66514-1-Ig, Proteintech, 1:5000 dilution), GAPDH (#60004-1-Ig, Proteintech, 1:10000 dilution), ZBTB16 (#66672-1-Ig, Proteintech, 1:2000 dilution), TOP2A (#20233-1-AP, Proteintech, 1:10000 dilution), Flag-tag (#66008-4-Ig, Proteintech, 1:5000 dilution), H3K4me3 (#A22146, ABclonal, 1:10000 dilution), and HA-tag (#51064-2-AP, Proteintech, 1:5000 dilution) were employed for western blot analysis. KDM5B (#A14104, ABclonal, 1:500 dilution), and USP7 (#66514-1-Ig, Proteintech, 1:500 dilution) were used for the immunohistochemistry of tissue microarray (#HNasN132Su01). Cisplatin (#S1166), and MG132 (#S2619) were obtained from the Selleck (Shanghai, China). Tissue microarray slides (#HNasN132Su01) were purchased from Outdo Biotech, Shanghai, China. The method of scoring the staining intensity was described previously [1].

Public datasets for data mining and bioinformatics analysis

(1) GSE118719: Transcriptome data NPC tissues were obtained from the GEO database (https://www.ncbi.nlm.nih.gov/). Data from 7 NPC biopsy specimens and 4 normal nasopharyngeal mucosal specimens were acquired.

(2) GSE53819: Transcriptome data of NPC tissues were obtained from the GEO database (https://www.ncbi.nlm.nih.gov/). Data from 18 NPC tissues, 18 non-cancerous nasopharyngeal tissues and 2992 genes were acquired.

Western blot analysis and co-immunoprecipiation (co-IP)

The collected cells were lysed with RIPA buffer (#G2002, servicebio, China), containing phosphatase inhibitors (#G2007, servicebio, China) and 1% protease inhibitors (#G2007, servicebio, China). Then the cell lysates were centrifuged at 12,000 rpm at 4 °C for 25 minutes and collected the supernatants. And 5 × loading buffer was added to the supernatants and boiled 10min in 100 °C water. The concentration of protein in cell lysates was detected by enhanced BCA protein analysis kit (#G2026, servicebio, China). The same amount of protein was loaded in each well of SDS-PAGE gels. The protein in the gels were transferred to the PVDF membranes. Then PVDF membranes were blocked with 5% non-fat milk at room temperature for 1 h and incubated with the primary antibody at 4 ℃. The next day, the membranes were washed with 1 × TBST 3 times (10 minutes each time) and incubated with the second antibody for 1 h. Then the membranes were washed again and exposed in dark field using ECL detection reagents (#G2020, servicebio, China). For co-immunoprecipiation (IP), collected cells were added with 1mL RIPA protein lysate containing 1 % protease

inhibitors on ice for at least 30 min. The supernatant was collected and co-cultured with Protein A and G Agarose beads (#G1718, Santa Cruz, America) and primary antibodies or IgG for 24 h at 4 °C. Then, the beads were washed with NETN buffer 6 times, added with 50 uL sample loading buffer, and boiled for 15 mins. Then, these samples were subjected to western blotting analysis. The antibodies used as follows: KDM5B (#A14104, ABclonal, 1:200 dilution), USP7 (#66514-1-Ig, Proteintech, 1:500 dilution).

Plasmids and siRNA transfection and shRNA infection

USP7, KDM5B, ZBTB16, and TOP2A were obtained from WZ Bioscience (Shandong, China) and GeneChem (Shanghai, China). Flag-USP7 was cloned into the CMV-MCS-3xFlag-SV40-neomycin vector by GENECHEM (Shanghai, China). Myc-KDM5B was cloned into the pEnter vector by GENECHEM (Shanghai, China). His-ZBTB16 was cloned into the pEnter vector with C-terminal Flag and His tag by WZ Bioscience (Shandong, China). HA-TOP2A was cloned into the pCMV-N-HA vector. pGEX-4T-1 vector was used to construct GST-tag plasmids. Short hairpin RNAs (shRNAs) were obtained from GeneChem (Shanghai, China), and the siRNA was purchased from RiboBio (Guangzhou, China). siRNA transfection was performed using Lipofectamine 2000 (Thermo Fisher Scientific, China), and 50 nM siRNAs were used. At least 48 hours later, cells were harvested and analyzed. The method of shRNA transfection was described previously [1]. The sequences of the shRNAs and siRNAs are provided in Supplementary Table S1.

Chromatin immunoprecipitation (ChIP) and ChIP-qRT-PCR

For ChIP-qPCR, ChIP Assay Kit (#P2078, Beyotime, China) were used, following the protocol of the manufacturer's instructions. Immunoprecipitation was performed using the appropriate antibodies as follows: KDM5B (#15327, Cell Signaling Technology, 1:50 dilution), H3K4me3 (#9751, Cell Signaling Technology, 1:50 dilution), and ZBTB16 (#66672-1-Ig, Proteintech, 1:50 dilution) were used for ChIP assay. Mouse IgG antibody (#61656S, Cell Signaling Technology, 1:1000 dilution) and rabbit IgG antibody (#3900S, Cell Signaling Technology, 1:1000 dilution) were used as a negative control. The primers were designed according to the promoter sequences of the genes of interest. The sequences of the ChIP-qPCR primers are shown in Supplementary Table S3.

In vitro cell proliferation assay and CCK-8 assay

Cell proliferation assay was performed following the manufacturer's instruction of the Cell Counting Kit-8 (CCK-8). Briefly, 1 × 103 cells were seeded in 96-well plates and cultured with 100 μL medium. 20 μL of the CCK-8 reagent (#C0037, Beyotime) was added to each well one hour before the end of the incubation period following the manufacturer's instructions. The absorbance values at 450 nm were measured using a Multimode Plate Reader (EnSpire® 2300, USA). CCK-8 assay was applied to measure the half maximal inhibitory concentration (IC_{50}) of cisplatin after treated with a serial dose of cisplatin for 24 h in HNE1 and CNE2 cells.

Cell invasion assay

Transwell chambers (8.0 μm Pore Size, CORNING) were used to assess the cell invasion ability. Cells were seeded into the Matrigel-coated invasion upper chamber with serum-free 1640 medium, and Medium containing 30% FBS was placed in the lower chambers and incubated for 18 hours in cell incubator. After incubation, the cells were fixed in methanol for 30 mins and then stained with Crystal Violet stain solution (#C0121, Beyotime). Invasion cells were evaluated under a microscope at five fields per well. All the experiments were repeated three times.

Colony formation assay

In this study, cell proliferation was evaluated using the colony formation assay. Cells were seeded into 6-well plates (1000 cells per well) and cultured in the 1640 medium containing 10% FBS at 37 °C. After 2 weeks, the cells were fixed in methanol for 30 min, stained with Crystal Violet Staining Solution for 20 mins, and washed 3 times with 1 × PBS. The number of colonies was counted and recorded. All the experiments were repeated three times.

Transcriptome sequencing (RNA-seq)

Total RNA was extracted by using TRIzol reagent (#R401-01 RNA isolater Total RNA Extraction Reagent, vazyme, Nanjing, China), and transcriptome sequencing was performed by NOVOGENE (Beijing, China) based on the Illumina platform. The library preparations were sequenced on an Illumina NovaSeq platform, and 150 bp

paired-end reads were generated. The RNA-seq data is provided in Supplementary Tables S4.

Statistical analysis

Each assay was performed in at least three independent experiments. Data were presented as the mean ± SD. GraphPad Prism 8 software was used to calculate the P value using Student's t-test for between-group comparisons and one-way ANOVA or two-way ANOVA for multiple comparisons. P value < 0.05 was considered statistically significant. In all cases, the significance of differences was indicated as follows: *P < 0.05; **P < 0.01; ***P < 0.001; not significant (ns), P > 0.05.

[1] Zhang, B., Cheng, X., Zhan, S., Jin, X., & Liu, T. MIB1 upregulates IQGAP1 and promotes pancreatic cancer progression by inducing ST7 degradation. Mol Oncol, 15(11): 3062-3075. (2021)

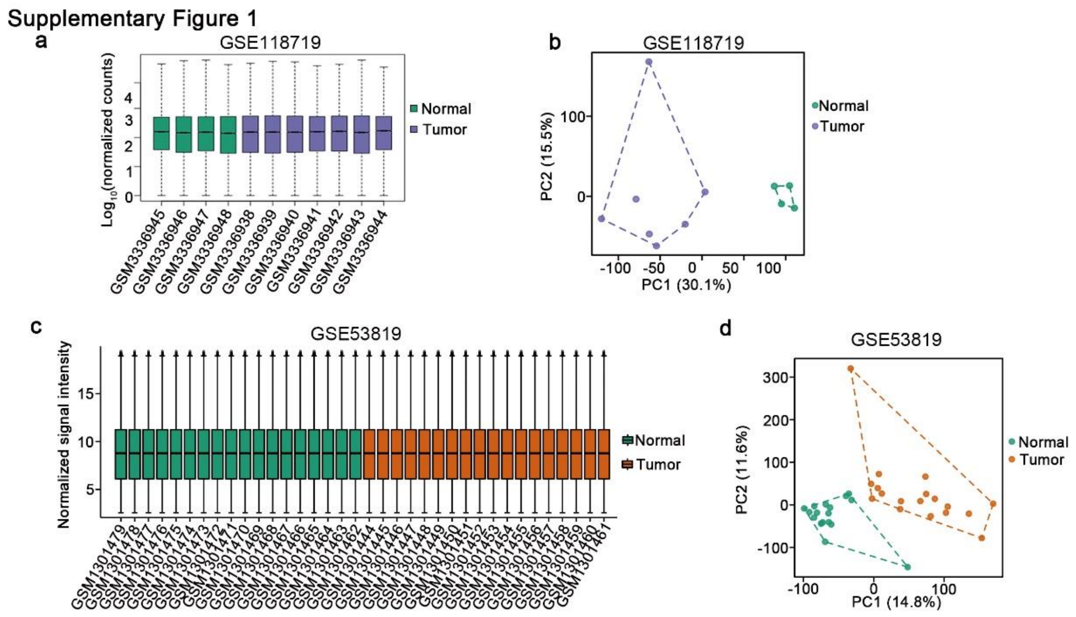

Supplementary figure 1. The visualization of the quality control of the GSE118719 and GSE53819 datasets.

a-d The GSE118719 and GSE53819 transcriptome datasets of NPC patients were obtained from the GEO database (https://www.ncbi.nlm.nih.gov/). Principle Component Analysis (**b, d**) and boxplot **(a, c**) to show the quality control of these two datasets.

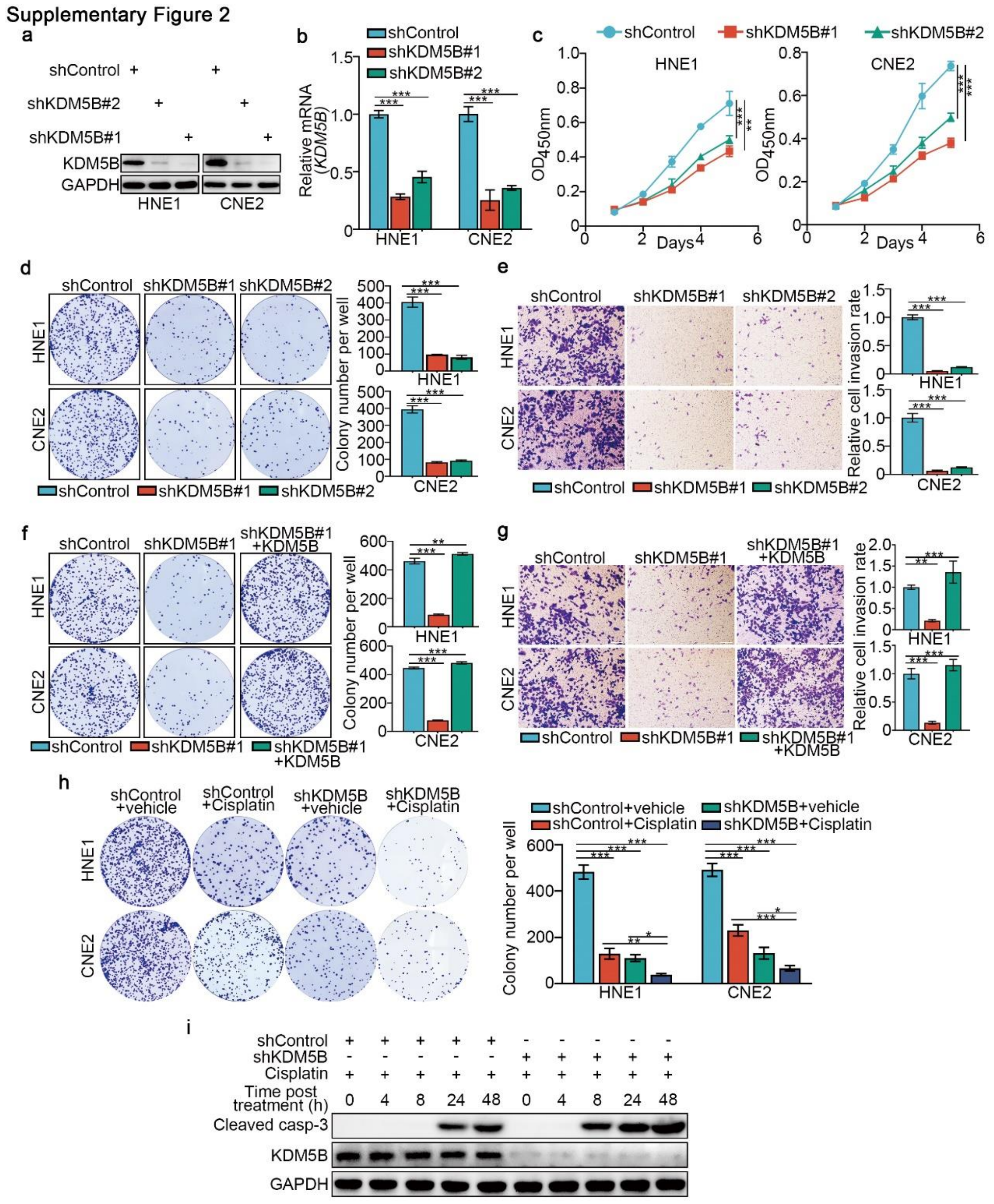

Supplementary figure 2. KDM5B promotes NPC cell proliferation and resistance to cisplatin.

a-e HNE1 and CNE2 cells infected with shControl, shKDM5B #1 or shKDM5B #2 for 48 h. Cells were harvested for Western blotting analysis (a), RT-qPCR analysis (b), CCK-8 assay (c), colony formation assay (d) and transwell assay (e). **f and g** HNE1

and CNE2 cells infected with shControl, shKDM5B or shKDM5B+myc-KDM5B for 48 h. Cells were harvested for colony formation assay (f) and transwell assay (e). **h** HNE1 and CNE2 cells were infected with shControl or shKDM5B plasmid for 48 hours. Then cells were treated with or without cisplatin (4 μg/ml) for another 48 hours. Cells were collected for colony formation assay. **i** Cells were treated with cisplatin for 0h, 4h, 8h, 24h, 48h. Cells were harvested for Western blotting analysis with indicated antibodies. Statistical significance was determined by one-way ANOVA followed by Tukey's multiple comparisons test. Data presented as Mean ±SD with three replicates. *P<0.05; **P<0.01; ***P<0.001.

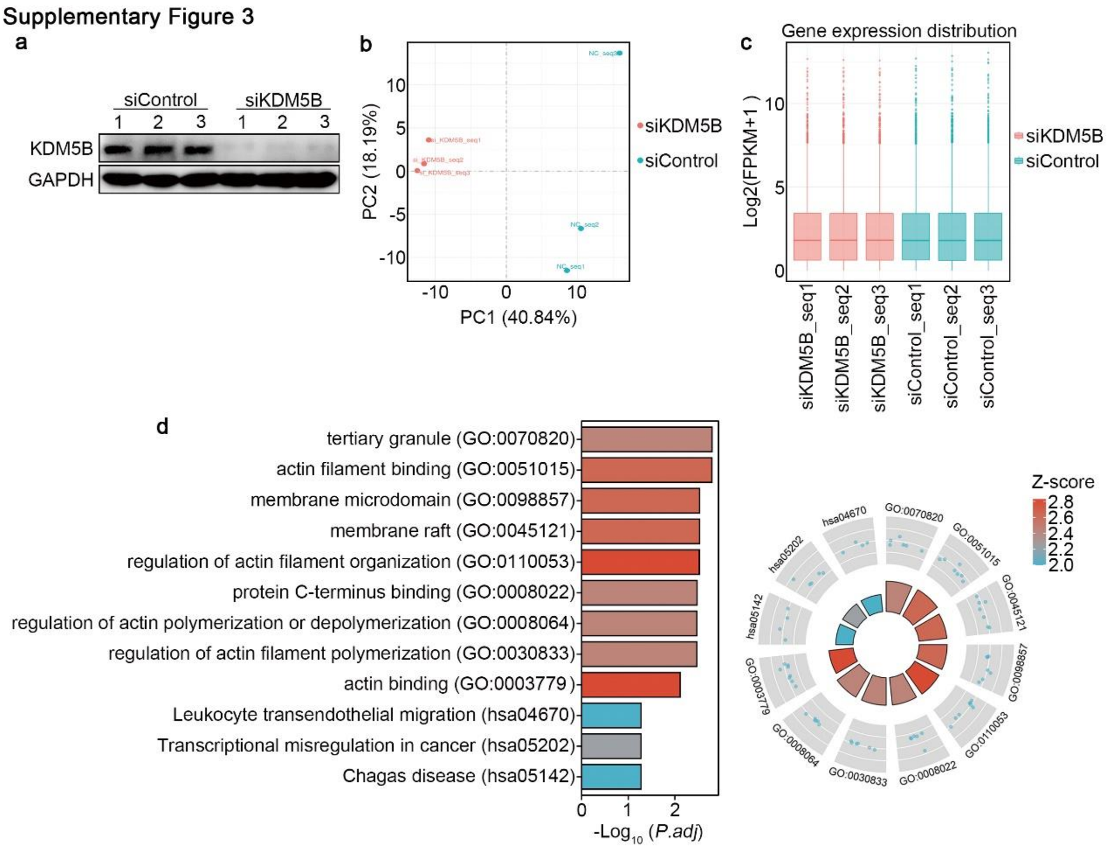

Supplementary figure 3. a HNE1 cells infected with siControl, siKDM5B for 48 h. Cells were harvested for Western blotting analysis before RNA-seq analysis. **b-c**

Principle Component Analysis (b) and boxplot (c) to show the quality control of RNA sequencing. **d** The GO enrichment analysis of 61 candidate genes.

Supplementary Figure 4

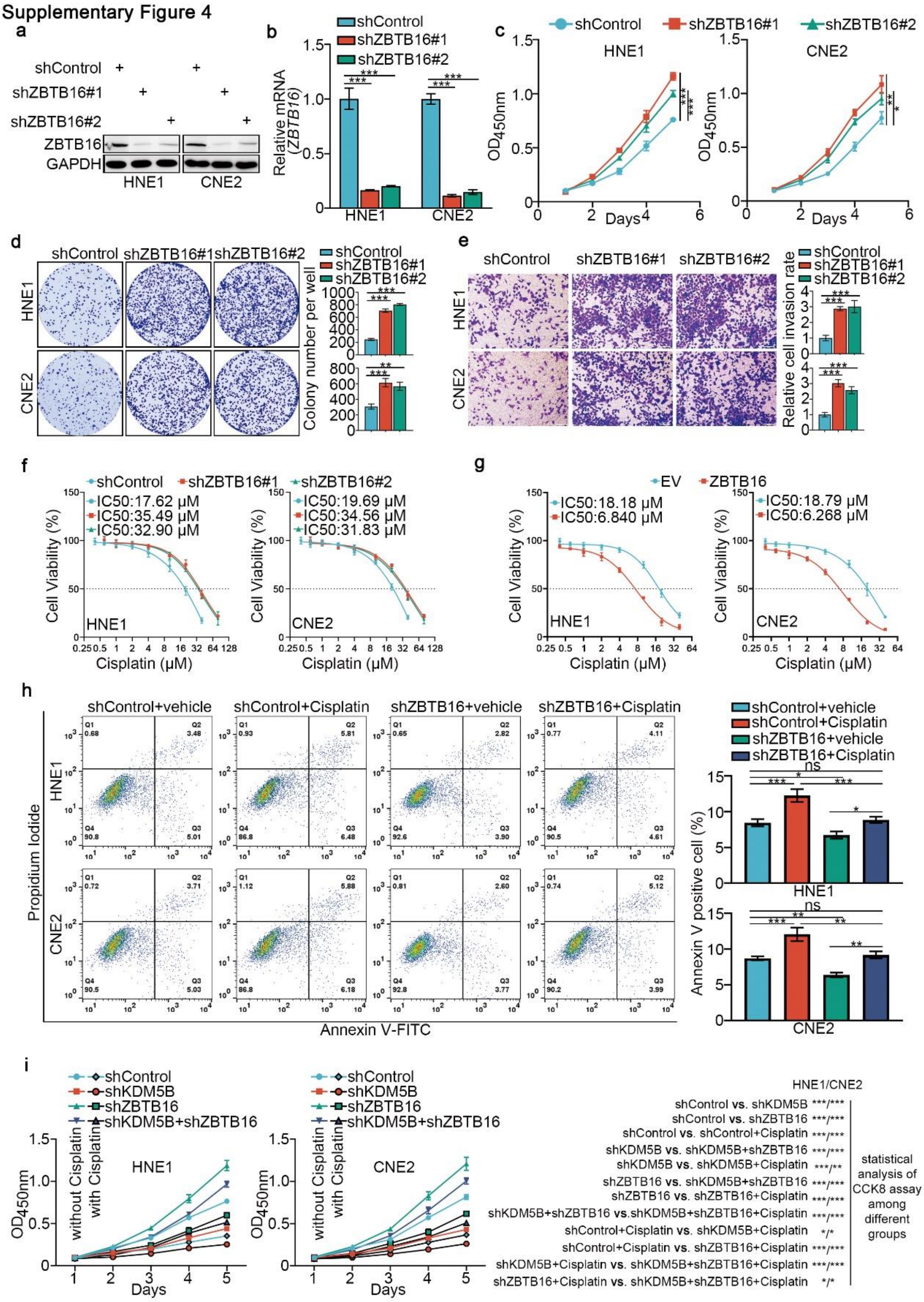

Supplementary figure 4. ZBTB16 inhibits the progression and cisplatin resistance of NPC.

a-e HNE1 and CNE2 cells infected with shControl, shZBTB16 #1 or shZBTB16 #2 for 48 h. Cells were harvested for Western blotting analysis (a), RT-qPCR analysis (b), CCK-8 assay (c), colony formation assay (d) and transwell assay (e). Statistical significance was determined by one-way ANOVA followed by Tukey's multiple comparisons test. Data presented as Mean ±SD with three replicates. *P<0.05; **P<0.01; ***P<0.001. **f and g** HNE1 and CNE2 cells infected with shZBTB16 #1, shZBTB16 #2 or ZBTB16 plasmids for 72 h. Cells were treated with a serial dose of cisplatin. Then, these cells were collected for CCK-8 assay and subjected to measure the IC50 values of cisplatin. **h** HNE1 and CNE2 cells were infected with shControl or shZBTB16 plasmid for 48 hours. then cells were treated with or without cisplatin (4 μg/ml) for another 48 hours. Cells were collected for fluorescein isothiocyanate (FITC)/PI flow cytometry. Statistical significance was determined by one-way ANOVA followed by Tukey's multiple comparisons test. Data presented as Mean ±SD with three replicates. NS not significant; *P<0.05; **P<0.01; ***P<0.001. **i** HNE1 and CNE2 cells were infected with shControl, shKDM5B or shZBTB16 plasmid for 48 hours. Then cells were treated with or without cisplatin (4 μg/ml) for another 48 hours. Cells were collected for colony formation assay. Statistical significance was determined by one-way ANOVA followed by Tukey's multiple comparisons test. Data presented as Mean ±SD with three replicates. *P<0.05; ***P<0.001.

Supplementary Figure 5

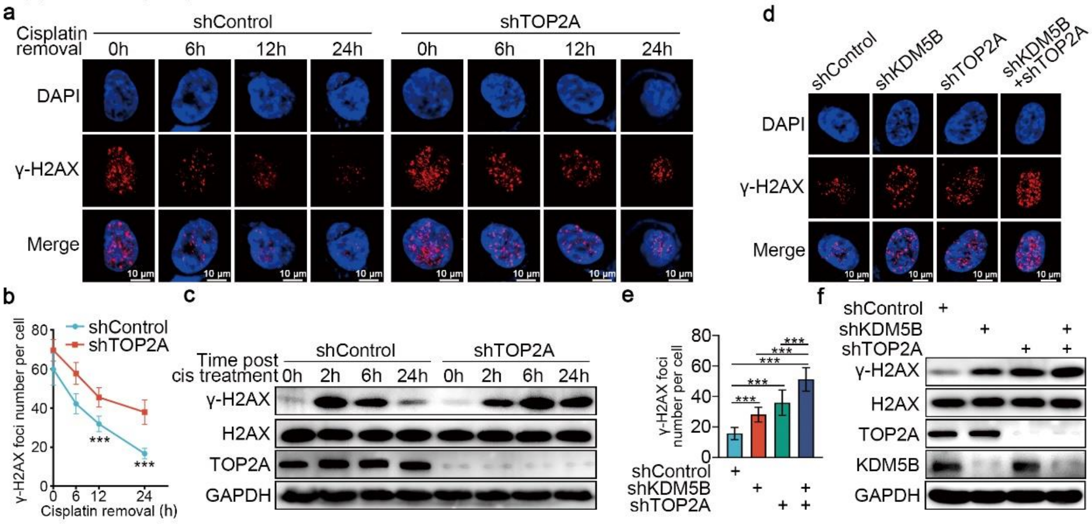

Supplementary figure 5. KDM5B promotes the repair of cisplatin-induced DSBs in NPC cells through TOP2A. a-b After treating HNE1 cells with cisplatin (4 μg/ml) for 8 hours, the cisplatin was removed. Immunofluorescence (**a**) was performed in HNE1 to detect γH2AX foci after cisplatin removal for 0, 6, 12 and 24 hours. The statistical graph of this γH2AX foci assay(**b**). **c** HNE1cells were harvested for Western blotting analysis after cisplatin treatment for 0h, 2h, 6h, 24h. **d-f** HNE1 cells infected with shControl, shKDM5B or shTOP2A for 48 h. Cells were harvested for Western blotting analysis (**f**) and γH2AX foci assay (**d**). The statistical graph of this γH2AX foci assay (**e**). Statistical significance was determined by one-way ANOVA followed by Tukey’s multiple comparisons test. Data presented as Mean ±SD with three replicates. ***P<0.001.

Supplementary Figure 6

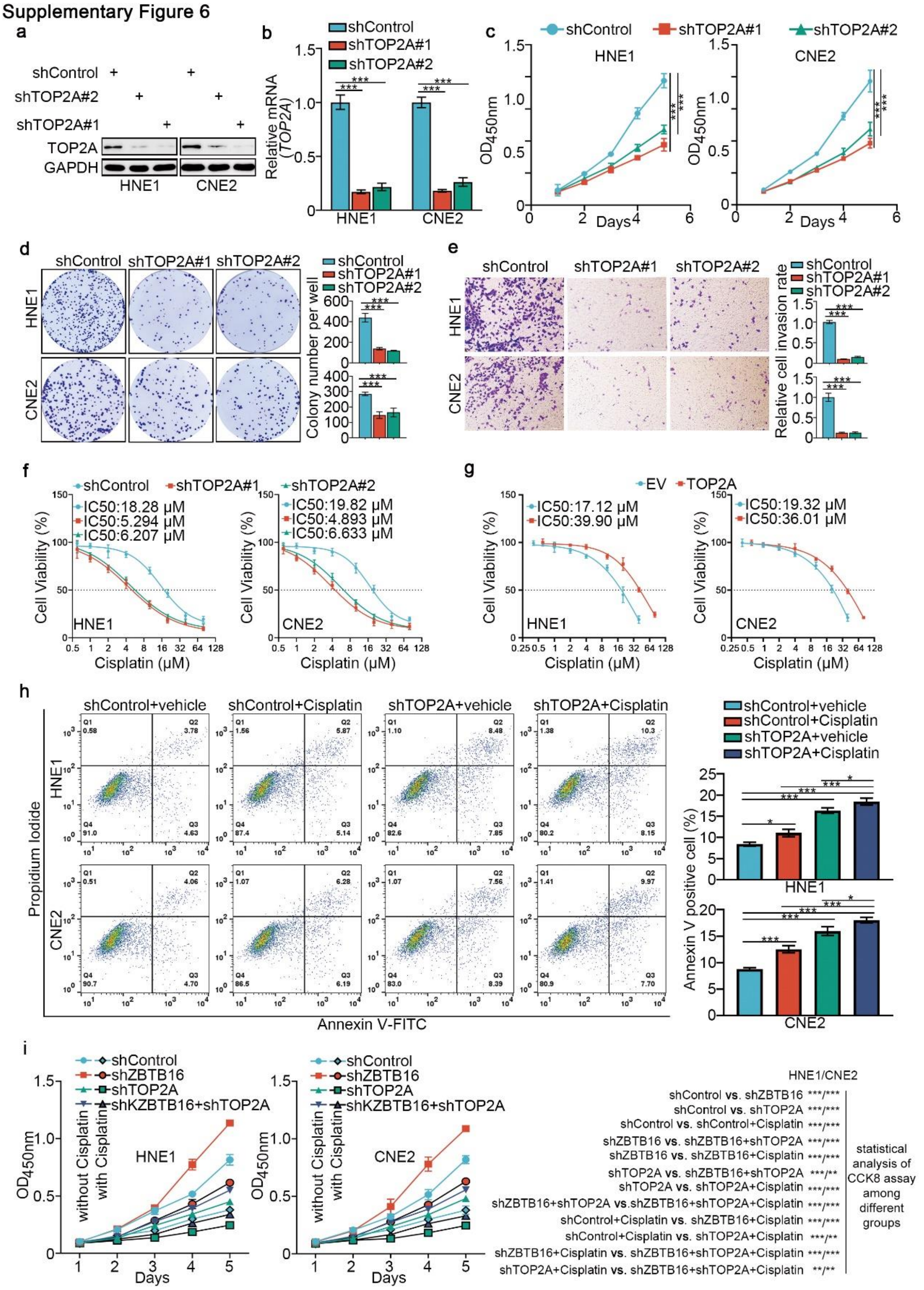

Supplementary figure 6. TOP2A promotes the progression and cisplatin resistance of NPC.

a-e HNE1 and CNE2 cells infected with shControl, shTOP2A #1 or shTOP2A #2 for 48 h. Cells were harvested for Western blotting analysis (a), RT-qPCR analysis (b), CCK-8 assay (c), colony formation assay (d) and transwell assay (e). Statistical significance was determined by one-way ANOVA followed by Tukey's multiple comparisons test. Data presented as Mean ±SD with three replicates. ***P<0.001. **f and g** HNE1 and CNE2 cells infected with shTOP2A #1, shTOP2A #2 or TOP2A plasmids for 72 h. Cells were treated with a serial dose of cisplatin. Then, these cells were collected for CCK-8 assay and subjected to measure the IC50 values of cisplatin. **h** HNE1 and CNE2 cells were infected with shControl or shTOP2A plasmid for 48 hours. Then cells were treated with or without cisplatin (4 μg/ml) for another 48 hours. Cells were collected for fluorescein isothiocyanate (FITC)/PI flow cytometry. Statistical significance was determined by one-way ANOVA followed by Tukey's multiple comparisons test. Data presented as Mean ±SD with three replicates. NS not significant; *P<0.05; ***P<0.001. **i** HNE1 and CNE2 cells were infected with shControl, shZBTB16 or shTOP2A plasmid for 48 hours. Then cells were treated with or without cisplatin (4 μg/ml) for another 48 hours. Cells were collected for colony formation assay. Statistical significance was determined by one-way ANOVA followed by Tukey's multiple comparisons test. Data presented as Mean ±SD with three replicates. **P<0.01; ***P<0.001.

Supplementary Figure 7

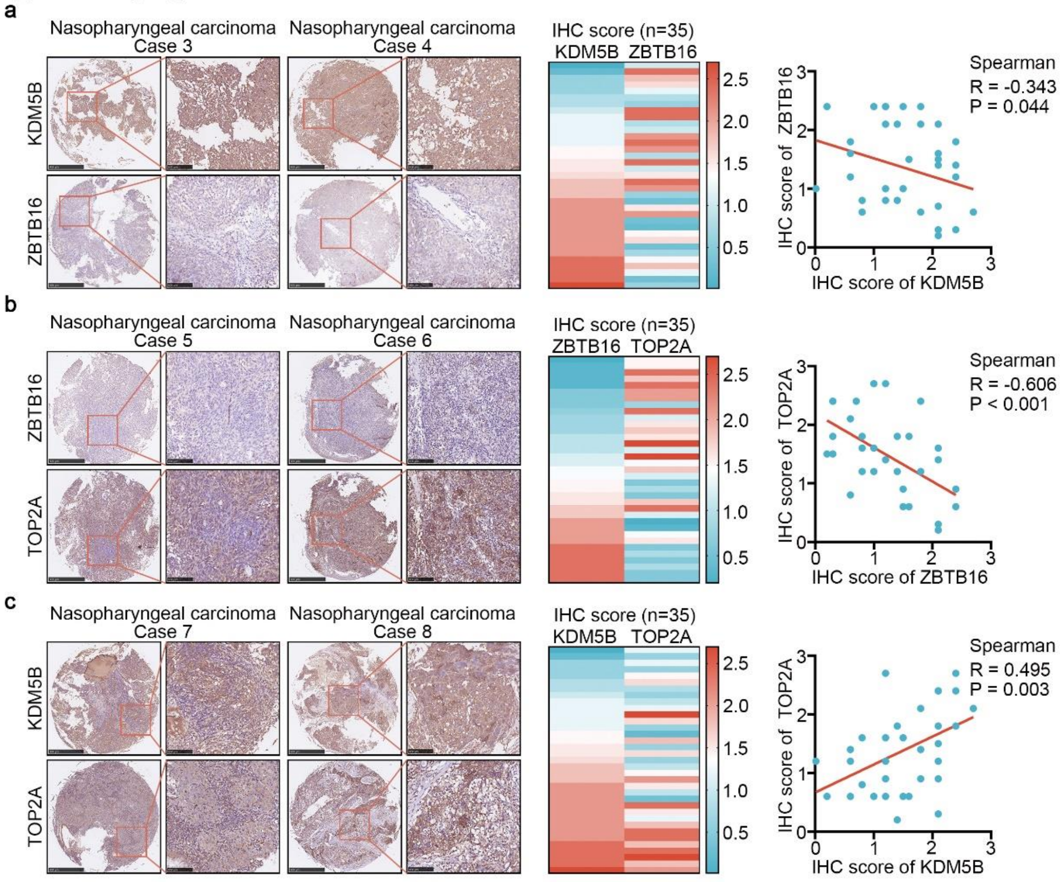

Supplementary figure 7. The relationship among KDM5B, ZBTB16 and TOP2A at the protein level.

a-c The tissue microarrays of NPC were stained with KDM5B, ZBTB16 and TOP2A, respectively (n = 35). The typical IHC images stained with KDM5B, ZBTB16 and TOP2A are shown in panel. The size of the scale bar on microscopy images as indicated in the figure. The expression levels of KDM5B, ZBTB16 and TOP2A are shown in the heatmap. The correlation of these three proteins was shown in panel. Spearman correlation was used to determine statistical significance. The P value was indicated in the figure.

Supplementary Figure 8

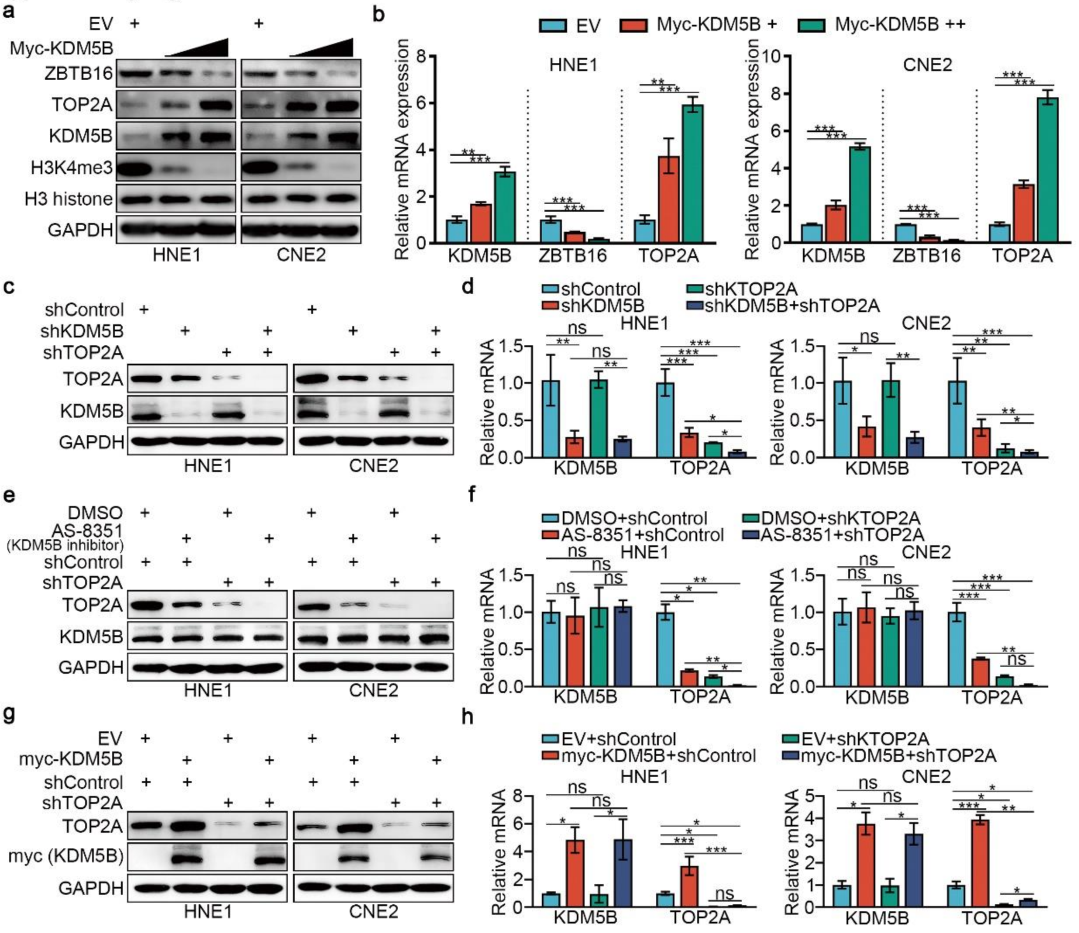

Supplementary figure 8. TOP2A was the crucial downstream of KDM5B, related to Figure 5.

a and b HNE1 and CNE2 cells were infected with indicated EV or Myc-KDM5B for 72 h. Cells were collected for Western blotting analysis (**a**), RT-qPCR analysis (**b**).

c and d HNE1 and CNE2 cells were infected with indicated shKDM5B or shTOP2A for 72 h. Cells were collected for Western blotting analysis (c), RT-qPCR analysis (d).

e and f HNE1 and CNE2 cells were infected with the indicated shTOP2A for 72 h. Cells were treated with or without KDM5B inhibitor AS-8351 (5 μM) for 24 h and harvested for Western blotting analysis (e), RT-qPCR analysis (f). **g and h** HNE1 and CNE2 cells were infected with shControl or shZBTB16 for 48 h. Then, cells were transfected with pcDNA3.1 or myc-KDM5B as indicated. After 24 h, cells were harvested for Western blotting analysis (g) and RT-qPCR analysis (h). Statistical significance was determined by one-way ANOVA followed by Tukey’s multiple

comparisons test. Data presented as Mean ± SD with three replicates. NS not significant; *P<0.05; **P< 0.01. ***P < 0.001.

Supplementary Figure 9

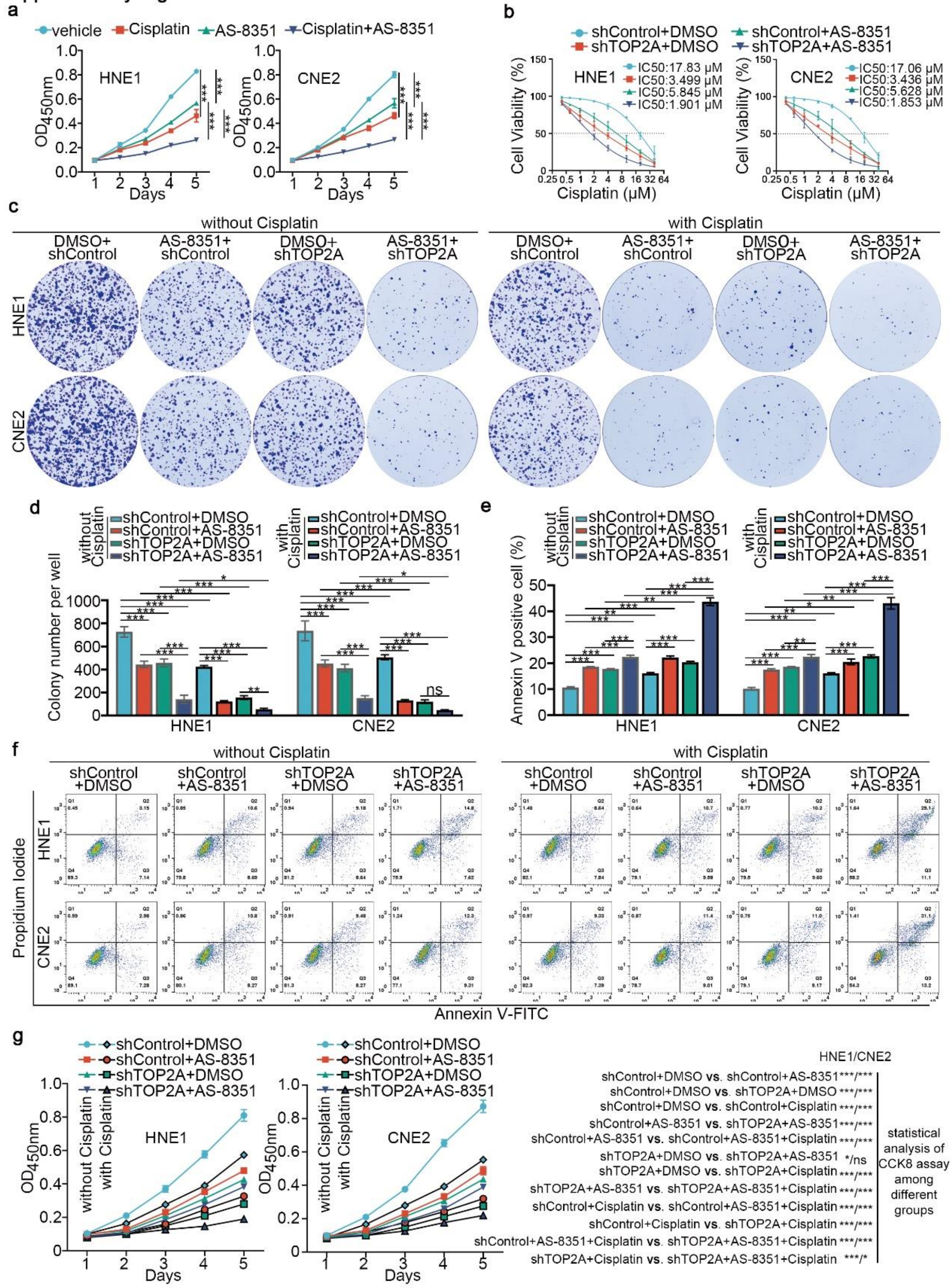

Supplementary figure 9. Targeting KDM5B and/or TOP2A can enhance the sensitivity of NPC cells to cisplatin.

a HNE1 and CNE2 cells were treated with or without KDM5B inhibitor AS-8351 (5 μM) in the presence or absence of cisplatin for 24 hours. Cells were collected for CCK-8 assay. **b** HNE1 and CNE2 cells were infected with shControl or shTOP2A plasmid for 48 hours. Then cells were treated with or without KDM5B inhibitor AS-8351 (5 μM) for 24 h in a serial dose of cisplatin. Then, these cells were collected for CCK-8 assay and subjected to measure the IC_{50} values of cisplatin. **c and d** HNE1 and CNE2 cells were infected with shControl or shTOP2A plasmid for 48 hours. Then cells were treated with or without KDM5B inhibitor AS-8351 (5 μM) for colony formation assay in the presence or absence of cisplatin. **e-g** HNE1 and CNE2 cells were infected with shControl or shTOP2A plasmid for 48 hours. Then cells were treated with or without KDM5B inhibitor AS-8351 (5 μM) for fluorescein isothiocyanate (FITC)/PI flow cytometry (e and f) and CCK-8 assay (g) in the presence or absence of cisplatin. Statistical significance was determined by one-way ANOVA followed by Tukey's multiple comparisons test. Data presented as Mean ±SD with three replicates. NS not significant; *P<0.05; **P<0.01; ***P<0.001.

Supplementary Figure 10

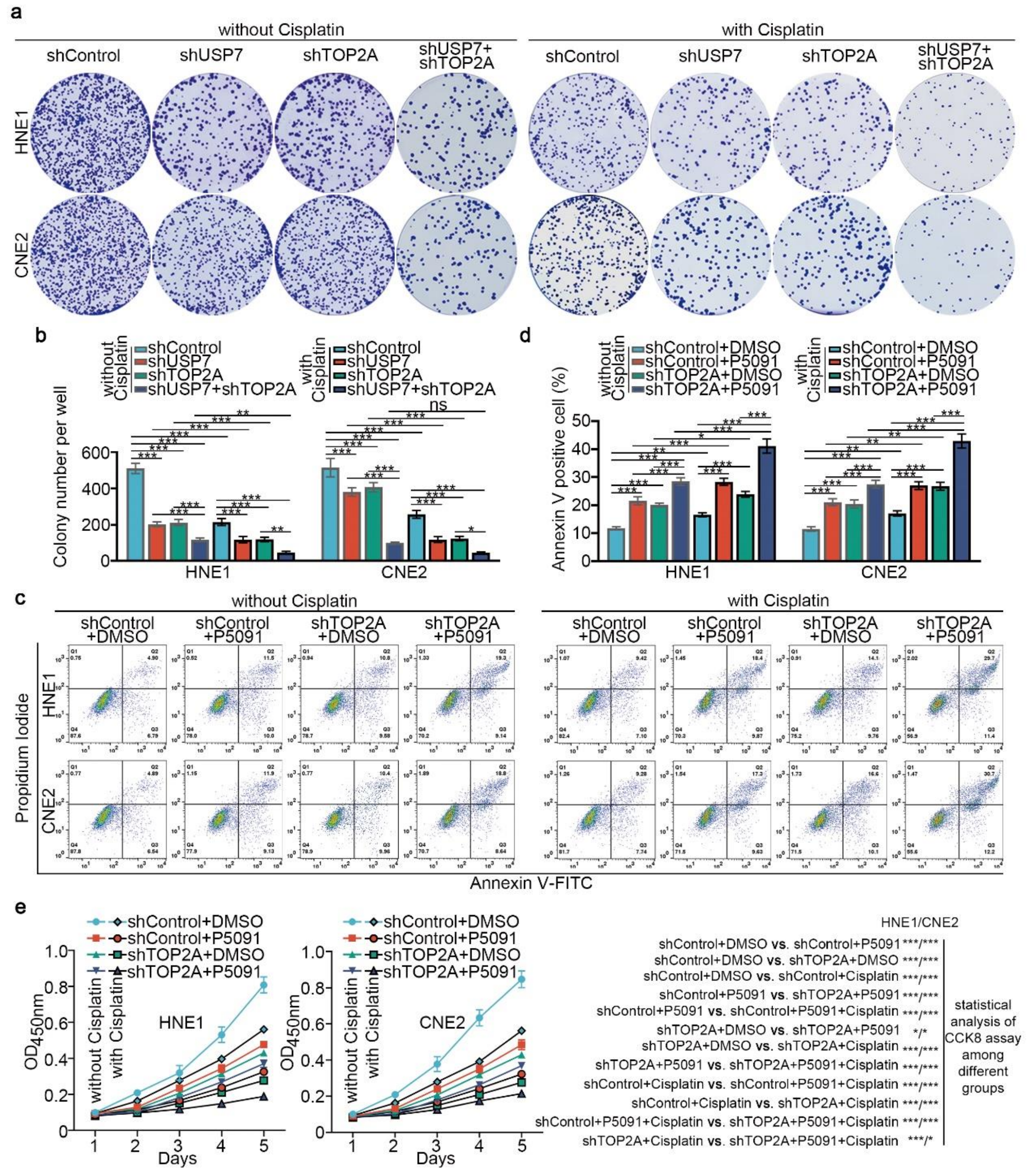

Supplementary figure 10. Targeting USP7 and/or TOP2A can enhance the sensitivity of NPC cells to cisplatin.

a and b HNE1 and CNE2 cells were infected with shUSP7 or shTOP2A plasmid for 48 hours. Then cells were treated with USP7 inhibitors P5091 (10 μM) for colony formation assay in the presence or absence of cisplatin. **c-e** HNE1 and CNE2 cells were infected with shControl or shTOP2A plasmid for 48 hours. Then cells were treated with

USP7 inhibitors P5091 (10 μM) for fluorescein isothiocyanate (FITC)/PI flow cytometry (c and d) and CCK-8 assay (e) in the presence or absence of cisplatin. Statistical significance was determined by one-way ANOVA followed by Tukey's multiple comparisons test. Data presented as Mean ±SD with three replicates. *$P<0.05$; **$P<0.01$; ***$P<0.001$.

Table S1. Sequences for shRNAs and siRNAs

Sequences for shRNAs:

shUSP7-1	5'-CCGGCCTGGATTTGTGGTTACGTTACTCGAGTAACGTAACCACAAATCCAGGTTTTTG-3'
shUSP7-2	5'-CCGGCCAGCTAAGTATCAAAGGAAACTCGAGTTTCCTTTGATACTTAGCTGGTTTTTG-3'
shKDM5B-1	5′-CCGGGCTCCCTTACTTTAGATGATACTCGAGTATCATCTAAAGTAAGGGAGCTTTTTG-3′
shKDM5B-2	5'-CCGGCCTGAGGAAGAGGAGTATCTTCTCGAGAAGATACTCCTCTTCCTCAGGTTTTTG-3'
shZBTB16-1	5'-CCGGGAATGCACTTACTGGCTCATTCTCGAGAATGAGCCAGTAAGTGCATTCTTTTTG-3'
shZBTB16-2	5'-CCGGGTGGACAGTTTGATGACCATACTCGAGTATGGTCATCAAACTGTCCACTTTTTG-3'
shTOP2A-1	5'-CCGGGCCTGATTTGTCTAAGTTTAACTCGAGTTAAACTTAGACAAATCAGGCTTTTTG-3'
shTOP2A-2	5'-CCGGGCTCCAAATCAATATGTGATTCTCGAGAATCACATATTGATTTGGAGCTTTTTG-3'

Sequences for siRNAs:

siKDM5B#1	5'-GGAAGATCTTGGACTTATT-3'
siKDM5B#2	5'-GCAGAATCTTACAACGAAT-3'

Table S2. Sequences for primers used for RT-qPCR

**Species**	**Gene**	**Forward (5'-3')**	**Reverse (5'-3')**
Human	*GAPDH*	CCAGAACATCATCCCTGCCT	CCTGCTTCACCACCTTCTTG
Human	*KDM5B*	AGTGGGCTCACATATCAGAGG	CAAACACCTTAGGCTGTCTCC
Human	*USP7*	GGAAGCGGGAGATACAGATGA	AAGGACCGACTCACTCAGTCT
Human	*ZBTB16*	CCTCAGACGACAATGACACGG	CTCGCTGGAATGCTTCGAGAT

Human	*TOP2A*	ACCATTGCAGCCTGTAAATGA	GGGCGGAGCAAAATATGTTCC

Table S3. Sequences of ChIP-qPCR primers

**Species**	**Gene**	**Forward (5'-3')**	**Reverse (5'-3')**
Human	*ZBTB16 (Primer I)*	CCGCCAGCACTAAAGATGGA	TTCGCAGTACCCGCTCTCAT
Human	*ZBTB16 (Primer O)*	ATTGCTAAAACCGCGTGACC	CTGGAGCTGAGCGAAGGTAG
Human	*TOP2A (Primer I)*	TGACACTTCCATGGTGACGG	GAGTCAGGGATTGGCTGGTC
Human	*TOP2A (Primer O)*	TTCAGGTCCCCAGTAGAGCA	TCACGGAGGTGAGCAAAAGT

Table S4. RNA-seq of siKDM5B vs. siControl

gene_id	si_KDM5B	si_Control	log2FC	pvalue	padj	gene_name	gene_biotype
ENSG00000058085	2679.55434	9772.149045	-1.866737584	1.19E-307	1.96E-303	LAMC2	protein_coding
ENSG00000167601	681.2737036	4129.127151	-2.599128127	2.56E-273	2.10E-269	AXL	protein_coding
ENSG00000106366	6983.17104	21016.13117	-1.589521466	9.96E-243	5.45E-239	SERPINE1	protein_coding
ENSG00000096060	7470.809034	2640.028489	1.500693089	6.88E-168	2.83E-164	FKBP5	protein_coding
ENSG00000163283	6800.931609	2143.946576	1.665220323	4.62E-160	1.52E-156	ALPP	protein_coding
ENSG00000117139	411.6851235	2059.944744	-2.323656446	7.72E-160	2.11E-156	KDM5B	protein_coding
ENSG00000180447	1403.320653	198.23233	2.822257218	1.22E-154	2.85E-151	GAS1	protein_coding
ENSG00000089597	13588.63246	5075.218323	1.420730353	3.64E-147	7.47E-144	GANAB	protein_coding
ENSG00000214049	2004.807653	5596.389585	-1.48130113	2.25E-145	4.11E-142	UCA1	lincRNA
ENSG00000166741	2261.14142	553.7556844	2.029594898	1.49E-144	2.45E-141	NNMT	protein_coding
ENSG00000189221	2649.238184	715.2175459	1.889673305	5.78E-140	8.63E-137	MAOA	protein_coding
ENSG00000197632	1036.362619	3160.38622	-1.608969939	9.16E-132	1.25E-128	SERPINB2	protein_coding
ENSG00000167460	10318.22087	4648.114179	1.150444872	1.59E-125	2.01E-122	TPM4	protein_coding
ENSG00000186081	13231.24987	4161.946341	1.66844889	1.54E-119	1.80E-116	KRT5	protein_coding
ENSG00000111335	5708.492306	2276.43418	1.326408069	2.41E-116	2.64E-113	OAS2	protein_coding
ENSG00000117525	3520.237115	8482.858138	-1.268931703	6.54E-116	6.71E-113	F3	protein_coding
ENSG00000138757	570.5225915	1922.251969	-1.753129975	4.40E-109	4.25E-106	G3BP2	protein_coding
ENSG00000152377	256.5365797	1195.058724	-2.219733763	1.57E-103	1.44E-100	SPOCK1	protein_coding
ENSG00000125538	723.2887286	2247.501751	-1.635423418	1.06E-102	9.15E-100	IL1B	protein_coding
ENSG00000139289	1420.785198	3523.457283	-1.31040219	2.17E-101	1.78E-98	PHLDA1	protein_coding
ENSG00000139211	4680.897277	9760.010386	-1.060016731	7.63E-96	5.97E-93	AMIGO2	protein_coding
ENSG00000100605	962.3860155	2621.758556	-1.446115395	6.15E-94	4.59E-91	ITPK1	protein_coding
ENSG00000104327	349.6041352	1290.086694	-1.883764699	1.75E-91	1.25E-88	CALB1	protein_coding
ENSG00000130589	10640.38722	4370.958546	1.283399868	2.38E-90	1.63E-87	HELZ2	protein_coding
ENSG00000131238	1392.041218	3436.231566	-1.303561848	2.32E-86	1.53E-83	PPT1	protein_coding

ENSG00000114942	2201.639865	4829.608231	-1.133444204	2.95E-85	1.87E-82	EEF1B2	protein_coding
ENSG00000183486	2530.817142	1002.410905	1.336373901	8.26E-85	5.02E-82	MX2	protein_coding
ENSG00000113083	5771.99352	2752.720833	1.068315747	1.96E-84	1.15E-81	LOX	protein_coding
ENSG00000111319	6494.626549	3241.396524	1.002617053	1.56E-81	8.54E-79	SCNN1A	protein_coding
ENSG00000185745	2654.080041	1096.187156	1.27567072	9.56E-80	5.07E-77	IFIT1	protein_coding
ENSG00000204103	872.4322755	183.0472189	2.252530188	1.56E-78	8.00E-76	MAFB	protein_coding
ENSG00000149948	1576.506613	3784.014091	-1.263416369	2.60E-78	1.30E-75	HMGA2	protein_coding
ENSG00000130635	17505.4063	8362.601741	1.065714329	1.03E-76	5.00E-74	COL5A1	protein_coding
ENSG00000185499	1833.741768	627.7411155	1.545992479	1.25E-76	5.88E-74	MUC1	protein_coding
ENSG00000173210	2826.26145	1271.657031	1.152395384	4.88E-74	2.23E-71	ABLIM3	protein_coding
ENSG00000137959	4064.875443	1824.778271	1.155765272	2.18E-68	9.42E-66	IFI44L	protein_coding
ENSG00000090054	947.2518678	2316.269044	-1.289668964	3.58E-68	1.51E-65	SPTLC1	protein_coding
ENSG00000078018	1447.815711	402.0672621	1.849024431	4.49E-68	1.84E-65	MAP2	protein_coding
ENSG00000165949	9788.917494	4536.916904	1.109292747	6.06E-64	2.26E-61	IFI27	protein_coding
ENSG00000091409	2334.386296	4740.498118	-1.021911013	2.71E-63	9.90E-61	ITGA6	protein_coding
ENSG00000111913	443.531722	57.59083617	2.942751478	1.14E-62	4.07E-60	RIPOR2	protein_coding
ENSG00000130303	5603.366743	2498.782206	1.164857664	4.27E-62	1.46E-59	BST2	protein_coding
ENSG00000132274	1907.382564	752.5714618	1.341818249	2.79E-60	9.36E-58	TRIM22	protein_coding
ENSG00000138772	1369.236227	2991.230509	-1.127407221	4.04E-58	1.25E-55	ANXA3	protein_coding
ENSG00000153815	910.0946063	1994.871374	-1.132222572	1.26E-57	3.84E-55	CMIP	protein_coding
ENSG00000143537	4783.024834	2248.1071	1.088984722	1.75E-56	5.22E-54	ADAM15	protein_coding
ENSG00000158417	1305.709778	2674.495195	-1.03453045	1.13E-53	3.27E-51	EIF5B	protein_coding
ENSG00000273749	974.3228056	2101.669977	-1.108453603	8.51E-50	2.37E-47	CYFIP1	protein_coding
ENSG00000118985	1147.777972	2478.257675	-1.110149824	1.85E-49	5.06E-47	ELL2	protein_coding
ENSG00000094975	564.5550005	1414.859575	-1.324825826	4.64E-49	1.25E-46	SUCO	protein_coding
ENSG00000089127	2329.941322	1128.774528	1.045290757	1.32E-48	3.50E-46	OAS1	protein_coding
ENSG00000261780	347.7475716	954.4389765	-1.456442199	7.21E-48	1.82E-45	LINC02582	processed_transcript
ENSG00000196611	49.7537855	323.5479884	-2.699594617	1.48E-47	3.58E-45	MMP1	protein_coding
ENSG00000175348	911.5138304	1992.603316	-1.128058732	6.03E-46	1.44E-43	TMEM9B	protein_coding
ENSG00000154188	345.0740905	49.39731445	2.8015353	4.91E-45	1.15E-42	ANGPT1	protein_coding
ENSG00000049192	47.57795902	346.7447107	-2.870065665	2.28E-44	5.13E-42	ADAMTS6	protein_coding
ENSG00000070950	448.5692384	1250.319216	-1.479175179	5.85E-44	1.26E-41	RAD18	protein_coding
ENSG00000151414	801.0698505	1727.126483	-1.108605933	1.81E-43	3.77E-41	NEK7	protein_coding
ENSG00000134954	1684.375203	3473.943079	-1.044063459	2.27E-43	4.63E-41	ETS1	protein_coding
ENSG00000102996	865.9845418	334.9119672	1.370654235	1.70E-42	3.40E-40	MMP15	protein_coding
ENSG00000164171	1156.503389	2552.908978	-1.142238292	1.32E-41	2.61E-39	ITGA2	protein_coding
ENSG00000058091	1010.206703	429.7641582	1.232696125	2.75E-40	5.13E-38	CDK14	protein_coding
ENSG00000196072	284.7206144	738.4655161	-1.375508768	5.84E-40	1.07E-37	BLOC1S2	protein_coding
ENSG00000157693	529.5646824	1209.423642	-1.190326278	1.37E-39	2.42E-37	TMEM268	protein_coding
ENSG00000165806	936.3724954	368.006959	1.346871446	2.00E-39	3.45E-37	CASP7	protein_coding
ENSG00000150630	599.7218874	1268.112203	-1.079891369	4.17E-39	7.13E-37	VEGFC	protein_coding
ENSG00000126709	9399.345599	4441.535158	1.081387785	9.69E-39	1.64E-36	IFI6	protein_coding
ENSG00000123689	1197.471882	2738.670093	-1.193755841	1.59E-38	2.63E-36	G0S2	protein_coding

ENSG00000180776	562.3908994	1255.760172	-1.159229195	2.02E-38	3.31E-36	ZDHHC20	protein_coding
ENSG00000187908	263.946073	41.61958694	2.662633323	1.62E-37	2.57E-35	DMBT1	protein_coding
ENSG00000134326	688.5327266	265.0963276	1.377114802	2.81E-37	4.39E-35	CMPK2	protein_coding
ENSG00000166736	392.4480573	91.3716507	2.105908906	7.74E-36	1.14E-33	HTR3A	protein_coding
ENSG00000068078	829.557161	341.381484	1.280266494	1.49E-35	2.12E-33	FGFR3	protein_coding
ENSG00000155893	480.5019033	150.986912	1.671167213	5.27E-35	7.22E-33	PXYLP1	protein_coding
ENSG00000064652	184.9013477	580.0591918	-1.648696571	2.08E-34	2.80E-32	SNX24	protein_coding
ENSG00000104756	330.1802777	785.4375354	-1.2506862	3.60E-34	4.80E-32	KCTD9	protein_coding
ENSG00000039987	255.4207334	37.34736855	2.768552408	1.77E-33	2.33E-31	BEST2	protein_coding
ENSG00000174804	1047.633366	494.2676813	1.083715533	7.92E-33	1.01E-30	FZD4	protein_coding
ENSG00000031691	259.6444199	688.1971054	-1.406301383	1.10E-32	1.39E-30	CENPQ	protein_coding
ENSG00000049249	360.601148	817.8374543	-1.181488976	5.34E-32	6.63E-30	TNFRSF9	protein_coding
ENSG00000060491	1843.07617	822.4932112	1.163596253	5.42E-32	6.65E-30	OGFR	protein_coding
ENSG00000244398	880.3705795	1897.513926	-1.107931959	6.04E-32	7.35E-30	AC116533.1	processed_pseudogene
ENSG00000128923	294.417045	764.8556457	-1.377291458	1.43E-31	1.71E-29	MINDY2	protein_coding
ENSG00000204767	119.9165823	393.6099704	-1.714562171	6.14E-31	7.10E-29	INSYN2B	protein_coding
ENSG00000168453	1564.491881	730.7817939	1.097537033	7.82E-31	8.93E-29	HR	protein_coding
ENSG00000173432	3267.278534	1466.791489	1.155021393	1.57E-30	1.77E-28	SAA1	protein_coding
ENSG00000178150	201.3186224	533.5738188	-1.406598265	2.10E-30	2.34E-28	ZNF114	protein_coding
ENSG00000187193	788.5398913	335.792152	1.230674285	2.60E-30	2.87E-28	MT1X	protein_coding
ENSG00000221869	2737.70214	1253.852434	1.126277583	6.31E-30	6.77E-28	CEBPD	protein_coding
ENSG00000141959	1519.242518	720.0140926	1.076670147	2.60E-29	2.69E-27	PFKL	protein_coding
ENSG00000143845	424.9047952	144.7084801	1.554342437	2.74E-29	2.81E-27	ETNK2	protein_coding
ENSG00000109906	220.2232683	36.76680967	2.586348703	2.22E-28	2.19E-26	ZBTB16	protein_coding
ENSG00000231412	165.6728084	475.4886403	-1.520188158	3.34E-28	3.27E-26	AC005392.2	lincRNA
ENSG00000226380	46.35839467	244.6055203	-2.405738463	3.79E-28	3.68E-26	LINC-PINT	lincRNA
ENSG00000076554	289.4161822	665.8459946	-1.202622056	4.10E-28	3.94E-26	TPD52	protein_coding
ENSG00000157450	596.0780601	1290.635847	-1.114670434	4.63E-28	4.42E-26	RNF111	protein_coding
ENSG00000110446	662.24353	250.6272282	1.401048402	6.75E-28	6.33E-26	SLC15A3	protein_coding
ENSG00000133106	967.6647408	447.7963144	1.112543579	2.26E-27	2.07E-25	EPSTI1	protein_coding
ENSG00000158292	1122.605756	560.3380167	1.002702314	2.69E-27	2.44E-25	GPR153	protein_coding
ENSG00000116701	417.7903963	859.7121937	-1.041193686	6.85E-27	6.02E-25	NCF2	protein_coding
ENSG00000111077	832.5091933	409.827868	1.021996537	3.42E-25	2.83E-23	TNS2	protein_coding
ENSG00000157168	349.4883447	737.0579115	-1.07642869	2.89E-24	2.28E-22	NRG1	protein_coding
ENSG00000100767	415.5769331	150.1584564	1.468159424	3.25E-24	2.56E-22	PAPLN	protein_coding
ENSG00000181333	232.5372774	578.2160753	-1.314606716	1.60E-23	1.22E-21	HEPHL1	protein_coding
ENSG00000170989	103.2395638	317.026481	-1.618875948	3.81E-23	2.86E-21	S1PR1	protein_coding
ENSG00000141384	84.33555397	287.837631	-1.771339689	4.82E-23	3.58E-21	TAF4B	protein_coding
ENSG00000167191	126.320993	352.767213	-1.483174937	6.23E-23	4.61E-21	GPRC5B	protein_coding
ENSG00000205426	83.00607998	279.5406954	-1.750433435	6.81E-23	5.00E-21	KRT81	protein_coding
ENSG00000172667	265.1289475	566.2572048	-1.094640306	1.03E-22	7.40E-21	ZMAT3	protein_coding
ENSG00000144228	152.7648197	409.8575067	-1.423483237	1.04E-22	7.48E-21	SPOPL	protein_coding
ENSG00000129480	154.6795502	404.3725445	-1.385096263	1.07E-22	7.62E-21	DTD2	protein_coding

ENSG00000197766	975.4912851	436.202819	1.160332752	1.11E-22	7.84E-21	CFD	protein_coding
ENSG00000166147	890.8893088	441.2200206	1.014062813	6.77E-22	4.56E-20	FBN1	protein_coding
ENSG00000067798	149.8890675	420.5049753	-1.488843471	1.06E-21	7.08E-20	NAV3	protein_coding
ENSG00000260604	603.8031198	274.4883017	1.137027905	1.65E-21	1.08E-19	AL590004.3	lincRNA
ENSG00000003989	422.2049976	176.2856104	1.260566413	8.50E-21	5.29E-19	SLC7A2	protein_coding
ENSG00000186847	306.6851341	715.7859865	-1.223292267	1.64E-20	9.98E-19	KRT14	protein_coding
ENSG00000105855	669.2957531	316.7428452	1.080213386	1.88E-20	1.14E-18	ITGB8	protein_coding
ENSG00000091592	283.3229691	612.6208836	-1.113706382	2.17E-20	1.31E-18	NLRP1	protein_coding
ENSG00000109452	508.6149462	1072.980547	-1.077087547	7.93E-20	4.59E-18	INPP4B	protein_coding
ENSG00000185947	456.7926469	200.9854042	1.185160379	1.42E-19	8.07E-18	ZNF267	protein_coding
ENSG00000187134	194.953513	448.2174558	-1.199885898	2.10E-19	1.19E-17	AKR1C1	protein_coding
ENSG00000136286	579.3181789	257.5932744	1.169015663	2.81E-19	1.55E-17	MYO1G	protein_coding
ENSG00000149968	14.11166797	110.0267691	-2.960231637	3.41E-19	1.87E-17	MMP3	protein_coding
ENSG00000100292	316.1923364	671.9813397	-1.088031315	4.02E-19	2.18E-17	HMOX1	protein_coding
ENSG00000226145	57.86509993	245.8184842	-2.081460918	6.57E-19	3.50E-17	KRT16P6	transcribed_unprocessed_pseudogene
ENSG00000170571	356.7333561	736.9462688	-1.045730323	2.80E-18	1.44E-16	EMB	protein_coding
ENSG00000149596	569.9589501	245.1213514	1.215578841	1.23E-17	6.06E-16	JPH2	protein_coding
ENSG00000164400	68.34590348	213.9736568	-1.648495067	1.36E-17	6.70E-16	CSF2	protein_coding
ENSG00000144815	202.2800712	446.3666639	-1.141584349	2.48E-17	1.19E-15	NXPE3	protein_coding
ENSG00000164283	5.171145563	79.20753796	-3.94349557	2.72E-17	1.30E-15	ESM1	protein_coding
ENSG00000087303	307.2636556	125.7451285	1.28894404	6.81E-17	3.19E-15	NID2	protein_coding
ENSG00000184792	355.7455907	149.3810698	1.250519952	7.27E-17	3.38E-15	OSBP2	protein_coding
ENSG00000113657	145.7741474	337.1419717	-1.208736781	3.77E-16	1.64E-14	DPYSL3	protein_coding
ENSG00000264112	118.7463236	297.4719206	-1.324719384	6.27E-16	2.68E-14	AC015813.1	lincRNA
ENSG00000204611	272.0666976	108.3512061	1.32756479	2.30E-15	9.41E-14	ZNF616	protein_coding
ENSG00000254726	406.4431025	195.335973	1.056123988	5.73E-15	2.27E-13	MEX3A	protein_coding
ENSG00000120910	181.4567094	403.6948475	-1.153974356	8.22E-15	3.22E-13	PPP3CC	protein_coding
ENSG00000277758	507.2517991	246.2378444	1.042129231	1.05E-14	4.05E-13	FO681492.1	protein_coding
ENSG00000197142	395.0644927	195.8236165	1.012497475	1.28E-14	4.87E-13	ACSL5	protein_coding
ENSG00000075213	171.7181351	376.9960032	-1.13366145	1.75E-14	6.56E-13	SEMA3A	protein_coding
ENSG00000147100	31.34429895	141.9077997	-2.174942258	2.46E-14	9.09E-13	SLC16A2	protein_coding
ENSG00000134996	197.7581136	411.9981276	-1.058835494	4.04E-14	1.47E-12	OSTF1	protein_coding
ENSG00000119979	192.2537154	399.2771146	-1.053042747	1.03E-13	3.63E-12	FAM45A	protein_coding
ENSG00000124641	160.5709991	347.6163812	-1.116102151	2.18E-13	7.36E-12	MED20	protein_coding
ENSG00000107159	158.5597489	39.10936821	2.016993076	5.71E-13	1.84E-11	CA9	protein_coding
ENSG00000120833	172.6671771	352.6991323	-1.031381097	6.74E-13	2.15E-11	SOCS2	protein_coding
ENSG00000132530	112.813389	29.02337688	1.95764878	1.19E-12	3.73E-11	XAF1	protein_coding
ENSG00000144810	205.430562	74.89561911	1.454984073	1.71E-12	5.27E-11	COL8A1	protein_coding
ENSG00000136378	93.90390358	18.40300576	2.350287465	1.84E-12	5.67E-11	ADAMTS7	protein_coding
ENSG00000137745	144.4832006	44.67806424	1.692535223	2.15E-12	6.58E-11	MMP13	protein_coding
ENSG00000005469	115.0458341	260.3636387	-1.176919697	2.18E-12	6.63E-11	CROT	protein_coding
ENSG00000047936	289.4090783	124.9234886	1.212447055	3.85E-12	1.14E-10	ROS1	protein_coding
ENSG00000146700	34.80106792	128.7788571	-1.884484034	6.10E-12	1.76E-10	SSC4D	protein_coding

ENSG00000165124	278.7905802	127.1009282	1.134137804	8.51E-12	2.43E-10	SVEP1	protein_coding
ENSG00000137573	207.7355908	86.10778351	1.26952316	9.56E-12	2.71E-10	SULF1	protein_coding
ENSG00000173702	300.9006944	140.3324663	1.100947368	1.03E-11	2.91E-10	MUC13	protein_coding
ENSG00000182795	66.91007821	177.838898	-1.410655546	1.13E-11	3.20E-10	C1orf116	protein_coding
ENSG00000157601	215.336693	86.57338121	1.313673821	1.33E-11	3.70E-10	MX1	protein_coding
ENSG00000153071	139.483828	294.0420194	-1.074388001	1.36E-11	3.78E-10	DAB2	protein_coding
ENSG00000184497	70.29320033	180.9345827	-1.363668088	2.68E-11	7.13E-10	TMEM255B	protein_coding
ENSG00000174206	113.5028833	249.4107671	-1.1358034	3.11E-11	8.19E-10	C12orf66	protein_coding
ENSG00000239620	145.3375239	50.96389632	1.51197208	4.78E-11	1.24E-09	PRR20G	protein_coding
ENSG00000185885	2222.444682	906.9852765	1.292751793	5.55E-11	1.42E-09	IFITM1	protein_coding
ENSG00000138061	235.0382695	105.5641844	1.156016991	5.61E-11	1.43E-09	CYP1B1	protein_coding
ENSG00000161896	183.3172947	67.47308103	1.444035969	9.91E-11	2.43E-09	IP6K3	protein_coding
ENSG00000182585	170.2265582	64.84875396	1.393502913	1.81E-10	4.26E-09	EPGN	protein_coding
ENSG00000141664	364.2796189	179.1725831	1.02366285	3.68E-10	8.24E-09	ZCCHC2	protein_coding
ENSG00000129521	244.4729462	116.6029185	1.069072734	3.80E-10	8.51E-09	EGLN3	protein_coding
ENSG00000111863	33.22526187	109.1739774	-1.712006328	4.44E-10	9.85E-09	ADTRP	protein_coding
ENSG00000138615	88.96641616	20.01362095	2.158019789	4.93E-10	1.09E-08	CILP	protein_coding
ENSG00000059804	18.23771358	79.03268047	-2.11194013	5.09E-10	1.12E-08	SLC2A3	protein_coding
ENSG00000171033	45.45995234	138.3863691	-1.611771183	5.36E-10	1.17E-08	PKIA	protein_coding
ENSG00000181634	133.7397746	267.5467944	-1.001451566	6.06E-10	1.31E-08	TNFSF15	protein_coding
ENSG00000182263	133.392336	276.8413003	-1.054984789	6.98E-10	1.49E-08	FIGN	protein_coding
ENSG00000232472	107.8833307	223.9093366	-1.053446997	1.32E-09	2.70E-08	EEF1B2P3	processed_pseudogene
ENSG00000146147	36.1865244	0	7.581845052	2.08E-09	4.13E-08	MLIP	protein_coding
ENSG00000230882	100.5111634	213.808758	-1.088327144	2.33E-09	4.62E-08	AC005077.4	processed_pseudogene
ENSG00000168952	85.63919656	195.6511077	-1.192654284	3.20E-09	6.27E-08	STXBP6	protein_coding
ENSG00000248607	133.6820129	50.77865724	1.394532917	3.34E-09	6.52E-08	AC117422.1	antisense
ENSG00000065325	58.22767138	10.89240106	2.414326162	3.70E-09	7.16E-08	GLP2R	protein_coding
ENSG00000117594	95.15086949	205.7952795	-1.112472014	5.02E-09	9.51E-08	HSD11B1	protein_coding
ENSG00000128512	104.2228585	214.7355347	-1.041641681	6.12E-09	1.14E-07	DOCK4	protein_coding
ENSG00000144893	43.13382992	123.3388394	-1.512578138	6.56E-09	1.22E-07	MED12L	protein_coding
ENSG00000104892	33.13891926	100.7337488	-1.603930039	1.07E-08	1.93E-07	KLC3	protein_coding
ENSG00000143369	90.74520942	190.8755169	-1.07316554	1.35E-08	2.40E-07	ECM1	protein_coding
ENSG00000011465	46.71214116	6.869372368	2.770949688	1.45E-08	2.55E-07	DCN	protein_coding
ENSG00000198074	87.88611655	27.62787674	1.668460784	1.86E-08	3.21E-07	AKR1B10	protein_coding
ENSG00000157343	43.00981429	5.510649758	2.971558681	3.32E-08	5.53E-07	ARMC12	protein_coding
ENSG00000166510	79.60919895	171.2150153	-1.105784709	3.54E-08	5.86E-07	CCDC68	protein_coding
ENSG00000285106	28.701467	91.51022927	-1.667201071	3.70E-08	6.11E-07	AC016831.6	lincRNA
ENSG00000053918	101.2815466	35.71419167	1.506518764	5.10E-08	8.15E-07	KCNQ1	protein_coding
ENSG00000166922	19.03874583	71.86147787	-1.919999216	7.30E-08	1.14E-06	SCG5	protein_coding
ENSG00000225886	78.05970948	24.62389001	1.664882759	7.97E-08	1.23E-06	AL445490.1	antisense
ENSG00000117151	54.00946045	129.2866965	-1.259241552	8.30E-08	1.27E-06	CTBS	protein_coding
ENSG00000117407	90.48595074	183.8778203	-1.022828716	1.04E-07	1.56E-06	ARTN	protein_coding
ENSG00000168961	171.6859159	79.68927721	1.108491614	1.18E-07	1.76E-06	LGALS9	protein_coding

ENSG00000119698	89.44710056	185.5547933	-1.0522716	1.47E-07	2.16E-06	PPP4R4	protein_coding
ENSG00000133069	63.69878717	142.2993438	-1.15719439	1.99E-07	2.87E-06	TMCC2	protein_coding
ENSG00000144063	67.94368362	154.401576	-1.184813137	2.20E-07	3.15E-06	MALL	protein_coding
ENSG00000057657	61.68635761	138.1412337	-1.163659809	2.26E-07	3.22E-06	PRDM1	protein_coding
ENSG00000135119	47.98981427	118.3822082	-1.300792466	3.06E-07	4.26E-06	RNFT2	protein_coding
ENSG00000127129	51.61290244	10.91466702	2.238593303	3.37E-07	4.66E-06	EDN2	protein_coding
ENSG00000220517	38.72157298	6.126913268	2.657192751	6.62E-07	8.71E-06	ASS1P1	processed_pseudogene
ENSG00000105550	170.6656423	79.90916523	1.095616874	6.92E-07	9.06E-06	FGF21	protein_coding
ENSG00000181773	163.6234001	78.74080889	1.054692516	7.71E-07	1.00E-05	GPR3	protein_coding
ENSG00000256393	32.21089097	100.383408	-1.641815045	9.95E-07	1.26E-05	RPL41P5	processed_pseudogene
ENSG00000246985	11.16963995	48.07031785	-2.101138564	1.03E-06	1.30E-05	SOCS2-AS1	processed_transcript
ENSG00000169306	11.9040493	49.2314184	-2.048776346	1.07E-06	1.33E-05	IL1RAPL1	protein_coding
ENSG00000166394	7.190251962	42.51968909	-2.577796906	1.15E-06	1.43E-05	CYB5R2	protein_coding
ENSG00000132932	43.82085088	105.6050163	-1.269426301	1.32E-06	1.62E-05	ATP8A2	protein_coding
ENSG00000257219	60.65679574	133.4828014	-1.136798727	1.37E-06	1.68E-05	LINC02407	lincRNA
ENSG00000274943	18.04771111	59.41678645	-1.72130514	1.51E-06	1.83E-05	AC079684.1	sense_intronic
ENSG00000214357	147.1785276	70.85132168	1.056645497	1.61E-06	1.94E-05	NEURL1B	protein_coding
ENSG00000232909	35.92803988	4.449424531	3.016479513	1.66E-06	2.00E-05	AL157823.2	antisense
ENSG00000079263	28.9862681	79.19757931	-1.451808448	1.91E-06	2.26E-05	SP140	protein_coding
ENSG00000103313	61.85890474	19.12910325	1.693416857	2.07E-06	2.45E-05	MEFV	protein_coding
ENSG00000225383	47.60576579	111.1601979	-1.222757373	2.20E-06	2.59E-05	SFTA1P	lincRNA
ENSG00000067715	52.95690949	122.6536323	-1.209578241	2.44E-06	2.83E-05	SYT1	protein_coding
ENSG00000164120	130.4217852	60.06420169	1.119402574	3.10E-06	3.51E-05	HPGD	protein_coding
ENSG00000174640	131.5928762	64.50879476	1.027977448	3.18E-06	3.59E-05	SLCO2A1	protein_coding
ENSG00000153233	37.93530465	91.0947893	-1.263354943	3.27E-06	3.67E-05	PTPRR	protein_coding
ENSG00000248588	19.22172436	60.56337543	-1.653212589	3.42E-06	3.82E-05	AC008517.1	lincRNA
ENSG00000278619	153.3825844	75.21510391	1.025580016	3.51E-06	3.89E-05	MRM1	protein_coding
ENSG00000250771	24.0476957	1.365978394	4.137080785	3.55E-06	3.94E-05	AC106865.1	transcribed_unprocessed_pseudogene
ENSG00000188856	107.6884306	220.0988473	-1.03051345	4.25E-06	4.64E-05	RPSAP47	processed_pseudogene
ENSG00000137198	130.9625537	64.30206687	1.026882178	4.29E-06	4.68E-05	GMPR	protein_coding
ENSG00000196196	113.6714669	53.243137	1.092570401	7.28E-06	7.57E-05	HRCT1	protein_coding
ENSG00000277196	41.18099933	9.908785192	2.05435295	7.46E-06	7.73E-05	AC007325.2	protein_coding
ENSG00000064205	31.38644257	4.440318704	2.821392159	7.51E-06	7.76E-05	WISP2	protein_coding
ENSG00000167656	30.89724404	4.40218974	2.802525475	8.39E-06	8.58E-05	LY6D	protein_coding
ENSG00000197405	93.84530962	41.42516635	1.180600076	9.90E-06	9.99E-05	C5AR1	protein_coding
ENSG00000204616	62.91919568	21.61805323	1.542480319	1.10E-05	0.000109287	TRIM31	protein_coding
ENSG00000204176	130.1911874	62.74779231	1.05162577	1.19E-05	0.00011707	SYT15	protein_coding
ENSG00000210144	128.5886377	58.08140269	1.142925776	1.53E-05	0.000145046	MT-TY	Mt_tRNA
ENSG00000141744	58.63095437	19.81757038	1.563715619	1.69E-05	0.000158643	PNMT	protein_coding
ENSG00000179869	43.11347415	94.7408612	-1.1362753	1.90E-05	0.000175532	ABCA13	protein_coding
ENSG00000187689	38.47154448	87.16950734	-1.178636436	2.06E-05	0.000189716	AMTN	protein_coding
ENSG00000008118	28.91772793	73.47835162	-1.345301414	2.24E-05	0.000204145	CAMK1G	protein_coding
ENSG00000162520	61.20481908	21.1685386	1.530178816	2.60E-05	0.000231041	SYNC	protein_coding

ENSG00000117245	33.97776263	80.49455556	-1.243583337	2.78E-05	0.000244758	KIF17	protein_coding
ENSG00000131386	41.72021956	10.68161886	1.971501981	3.06E-05	0.000266676	GALNT15	protein_coding
ENSG00000182866	7.390258772	33.03729974	-2.161316072	3.25E-05	0.000281886	LCK	protein_coding
ENSG00000154556	36.37354647	8.596798767	2.084988672	3.28E-05	0.000283773	SORBS2	protein_coding
ENSG00000242779	77.12494623	32.79614297	1.231861599	3.52E-05	0.000301518	ZNF702P	transcribed_processed_pseudogene
ENSG00000004799	138.7512197	59.40283197	1.22650882	3.64E-05	0.000310519	PDK4	protein_coding
ENSG00000134539	30.37356188	5.412624477	2.47850143	3.93E-05	0.000332959	KLRD1	protein_coding
ENSG00000269896	51.08113321	15.77953152	1.695324172	4.33E-05	0.000362879	AL513477.1	transcribed_processed_pseudogene
ENSG00000162006	117.7990608	57.63486948	1.031196241	4.52E-05	0.000376491	MSLNL	protein_coding
ENSG00000163751	4.214167394	26.08248794	-2.635740627	4.90E-05	0.000405233	CPA3	protein_coding
ENSG00000224858	28.27668513	82.46378171	-1.539906411	5.10E-05	0.000419066	RPL29P11	processed_pseudogene
ENSG00000224389	34.60851211	7.124187872	2.275317032	5.52E-05	0.000450155	C4B	protein_coding
ENSG00000241494	30.18837711	84.79671862	-1.488897631	5.57E-05	0.000453596	AL355032.1	processed_pseudogene
ENSG00000144583	28.90368005	69.2724325	-1.261168036	6.02E-05	0.000486515	4-Mar	protein_coding
ENSG00000249364	22.60960606	3.092053357	2.873462742	6.15E-05	0.000495228	AC112206.2	lincRNA
ENSG00000169213	39.7566686	87.53443537	-1.135980435	6.15E-05	0.000495228	RAB3B	protein_coding
ENSG00000168874	34.69847119	8.166348618	2.086102988	7.39E-05	0.000581895	ATOH8	protein_coding
ENSG00000245648	94.74119862	45.65925578	1.055634216	8.28E-05	0.000643615	AC022075.1	antisense
ENSG00000185565	11.27628022	41.80247736	-1.891259931	8.39E-05	0.000651145	LSAMP	protein_coding
ENSG00000179583	77.38681637	33.97310653	1.190163815	8.44E-05	0.000654792	CIITA	protein_coding
ENSG00000135678	82.2960918	37.16745953	1.143955482	8.59E-05	0.000664725	CPM	protein_coding
ENSG00000182261	31.98568884	72.50569163	-1.177728799	9.88E-05	0.000750036	NLRP10	protein_coding
ENSG00000165548	39.92886863	11.23528319	1.82592565	9.92E-05	0.000752842	TMEM63C	protein_coding
ENSG00000144821	16.22116051	48.64012372	-1.577255736	0.000101229	0.000766094	MYH15	protein_coding
ENSG00000011201	55.03234548	110.5711075	-1.003090315	0.000101879	0.000769598	ANOS1	protein_coding
ENSG00000104760	42.88898805	12.70654265	1.757022946	0.000104892	0.000789095	FGL1	protein_coding
ENSG00000197467	14.70644363	44.66220124	-1.600086015	0.000106818	0.000801377	COL13A1	protein_coding
ENSG00000137878	47.78797017	96.41984576	-1.01507435	0.000120988	0.000899472	GCOM1	protein_coding
ENSG00000268350	53.34111792	11.60989132	2.198606631	0.000125786	0.000931234	FAM156A	protein_coding
ENSG00000116678	62.40480182	26.09970238	1.260114367	0.000145135	0.001058394	LEPR	protein_coding
ENSG00000133328	68.05889614	27.14911891	1.330229007	0.000152097	0.001105732	HRASLS2	protein_coding
ENSG00000226237	26.27305869	5.124232922	2.355002978	0.00015236	0.001106665	GAS1RR	lincRNA
ENSG00000174125	4.800428921	25.53017505	-2.405115109	0.00016067	0.001160862	TLR1	protein_coding
ENSG00000187151	42.61610856	14.04299929	1.603726582	0.000161396	0.001165089	ANGPTL5	protein_coding
ENSG00000066056	1.588790789	17.03112302	-3.411523108	0.000166699	0.001200206	TIE1	protein_coding
ENSG00000164744	27.73520053	63.54239333	-1.198288712	0.000212663	0.001487465	SUN3	protein_coding
ENSG00000186567	66.98045567	28.91169285	1.209955168	0.000221072	0.001542344	CEACAM19	protein_coding
ENSG00000137501	17.11555931	48.84770444	-1.505084611	0.00022956	0.001595459	SYTL2	protein_coding
ENSG00000155816	46.93436289	17.13099834	1.455350085	0.000237684	0.00164566	FMN2	protein_coding
ENSG00000102445	15.12569177	43.78571648	-1.534017588	0.000279133	0.001887307	RUBCNL	protein_coding
ENSG00000155093	75.78177147	35.17796185	1.108186115	0.000284923	0.001920923	PTPRN2	protein_coding
ENSG00000229953	95.94224449	46.85927964	1.030902611	0.000309741	0.002067875	AL590666.2	antisense
ENSG00000242960	235.3411982	501.8957918	-1.092070279	0.000313062	0.002087506	FTH1P23	processed_pseudogene

ENSG00000184838	36.10648969	76.44975952	-1.084459934	0.000335027	0.00220968	PRR16	protein_coding
ENSG00000285294	39.88587087	81.81677918	-1.036537313	0.000344542	0.002261614	LINC00842	lincRNA
ENSG00000197301	38.59337572	80.7727684	-1.069716085	0.000348072	0.002282961	AC090673.1	antisense
ENSG00000166262	18.22140134	52.28747138	-1.525513376	0.000358935	0.002343918	FAM227B	protein_coding
ENSG00000143434	85.21816925	42.08951748	1.01918558	0.000362535	0.002366486	SEMA6C	protein_coding
ENSG00000241935	80.79023615	37.85777671	1.091851462	0.000369092	0.002401653	HOGA1	protein_coding
ENSG00000248213	48.6957008	17.62697061	1.461749487	0.000381304	0.002466465	CICP16	processed_pseudogene
ENSG00000166482	63.6892099	28.31449383	1.169560824	0.000382786	0.00247508	MFAP4	protein_coding
ENSG00000153294	27.14822297	61.54108695	-1.177094375	0.000387995	0.002505801	ADGRF4	protein_coding
ENSG00000277142	48.94977284	19.49375274	1.329988283	0.000392237	0.002526345	LINC00235	lincRNA
ENSG00000256542	40.62177042	14.41355313	1.496358184	0.000402128	0.002582866	AC148477.2	antisense
ENSG00000169760	48.48236216	18.75719799	1.366661515	0.0004091	0.002622517	NLGN1	protein_coding
ENSG00000251169	34.12109275	8.83525266	1.943001395	0.000454377	0.002880176	LINC01843	lincRNA
ENSG00000223764	63.92654263	29.29675826	1.123680212	0.000518244	0.003226504	LINC02593	lincRNA
ENSG00000172967	66.8419651	28.25807753	1.246132861	0.000523828	0.003255101	XKR3	protein_coding
ENSG00000224260	28.4525816	7.844381012	1.855194606	0.000549553	0.003394421	AL023754.1	lincRNA
ENSG00000105696	36.83571239	76.48198413	-1.05274859	0.000570633	0.003508794	TMEM59L	protein_coding
ENSG00000133519	25.48833867	59.90948157	-1.227944747	0.000595431	0.003644907	ZDHHC8P1	transcribed_unprocessed_pseudogene
ENSG00000241749	39.20113613	81.69613372	-1.063081044	0.000653607	0.003946482	RPSAP52	transcribed_processed_pseudogene
ENSG00000244731	39.41447477	12.56988979	1.645718942	0.000663588	0.003996607	C4A	protein_coding
ENSG00000232316	15.1737962	1.699754697	3.151334007	0.000675196	0.004056112	LINC02518	lincRNA
ENSG00000280587	42.33351371	16.42076386	1.367359656	0.000713526	0.004266094	LINC01348	lincRNA
ENSG00000125798	16.12375043	41.42381492	-1.36290099	0.000742512	0.004418497	FOXA2	protein_coding
ENSG00000162894	76.31382967	36.00983901	1.085687134	0.000746776	0.004437445	FCMR	protein_coding
ENSG00000277400	35.3029041	11.54544209	1.608386924	0.000760215	0.004507521	AC145212.1	protein_coding
ENSG00000110427	37.06793846	13.38590395	1.4711234	0.000827601	0.004861375	KIAA1549L	protein_coding
ENSG00000205670	85.95419782	9.929201109	3.114268087	0.000869518	0.005064227	SMIM11A	protein_coding
ENSG00000127863	5.496311112	23.82906892	-2.117454023	0.000978527	0.005603766	TNFRSF19	protein_coding
ENSG00000234432	48.43758522	20.78937755	1.218925621	0.000979696	0.005608504	AC092171.3	lincRNA
ENSG00000117707	6.758446152	24.66066754	-1.867786305	0.001005162	0.005730328	PROX1	protein_coding
ENSG00000163395	10.7733809	35.1685018	-1.69900469	0.001041839	0.005902558	IGFN1	protein_coding
ENSG00000115705	15.03309938	2.020370867	2.882537433	0.001155708	0.006465199	TPO	protein_coding
ENSG00000248187	14.55496832	2.052595483	2.824710654	0.001181063	0.006577923	AC078850.1	lincRNA
ENSG00000260302	61.29116169	28.92485299	1.081178955	0.001201383	0.006682033	AP005482.1	lincRNA
ENSG00000102931	60.18313527	26.48263919	1.180340646	0.001233326	0.006838861	ARL2BP	protein_coding
ENSG00000177494	6.136637871	23.29333771	-1.927208932	0.001364404	0.007479867	ZBED2	protein_coding
ENSG00000224081	20.32791349	47.0789476	-1.214640354	0.001407566	0.00768061	SLC44A3-AS1	transcribed_processed_pseudogene
ENSG00000137673	16.06415146	2.758277054	2.549478118	0.001454767	0.007890992	MMP7	protein_coding
ENSG00000272872	42.29945715	16.28460962	1.373386156	0.001540126	0.00827091	AP000525.1	sense_intronic
ENSG00000253313	7.995038777	26.36632658	-1.717561869	0.001556257	0.008350522	C1orf210	protein_coding
ENSG00000125872	15.22935163	39.0637634	-1.363084288	0.001587038	0.008493497	LRRN4	protein_coding
ENSG00000269376	61.55233764	28.36443149	1.116786357	0.001600094	0.008549443	AL356740.3	sense_intronic

ENSG00000188897	46.73690941	20.20078574	1.210085047	0.001615168	0.008615979	AC099489.1	protein_coding
ENSG00000273472	62.40408578	30.67589678	1.024198609	0.001634607	0.008708366	AC096733.2	lincRNA
ENSG00000160868	2.869444307	15.76501995	-2.450303618	0.001639849	0.008733457	CYP3A4	protein_coding
ENSG00000228437	16.30119523	45.06660967	-1.462783239	0.001678099	0.008925595	LINC02474	lincRNA
ENSG00000181781	17.03666771	2.68927491	2.649600427	0.001694753	0.009002521	ODF3L2	protein_coding
ENSG00000272221	56.20409438	26.52987398	1.080269544	0.001764181	0.009323091	AL645933.2	lincRNA
ENSG00000226472	59.93459697	28.84161782	1.056828021	0.001766508	0.009332387	AC008013.1	transcribed_unprocessed_pseudogene
ENSG00000234290	69.60577416	34.40512817	1.012543157	0.001773424	0.009362904	AC116366.1	antisense
ENSG00000229619	26.10234885	6.513330104	2.003869807	0.001794947	0.009461336	MBNL1-AS1	antisense
ENSG00000226007	15.77453267	2.718796654	2.530871465	0.001835727	0.009648433	BX005266.2	lincRNA
ENSG00000233328	36.73425877	73.56499114	-1.00146245	0.001837617	0.009652191	PFN1P1	processed_pseudogene
ENSG00000065413	32.45155121	67.75229108	-1.060164536	0.001856606	0.009733479	ANKRD44	protein_coding
ENSG00000180264	53.77021044	25.60615444	1.070905655	0.001908612	0.009974077	ADGRD2	protein_coding
ENSG00000273045	17.00963509	40.85428759	-1.261783717	0.00205787	0.010642368	C2orf15	protein_coding
ENSG00000008323	54.96976611	26.36177367	1.062843113	0.002144243	0.011005907	PLEKHG6	protein_coding
ENSG00000086717	23.16883498	6.105145915	1.918026186	0.002151146	0.01103789	PPEF1	protein_coding
ENSG00000223459	22.52446468	50.07702998	-1.154389395	0.002227477	0.011404629	TCAF1P1	unprocessed_pseudogene
ENSG00000005001	9.985622359	30.69545987	-1.622184185	0.002309301	0.011772206	PRSS22	protein_coding
ENSG00000205809	56.51819245	27.67326149	1.03011479	0.00232191	0.011825474	KLRC2	protein_coding
ENSG00000127774	40.15143745	16.65921775	1.265817974	0.00234291	0.011917652	EMC6	protein_coding
ENSG00000268713	30.91129191	11.26430632	1.454648204	0.002354515	0.01196557	AC005261.3	lincRNA
ENSG00000130052	46.50135585	20.50233743	1.179655149	0.002413359	0.012219257	STARD8	protein_coding
ENSG00000284883	7.34619788	24.26969779	-1.719851468	0.002577737	0.012935924	AC021066.2	unprocessed_pseudogene
ENSG00000179593	24.33433409	7.544180759	1.692878743	0.002602877	0.01303819	ALOX15B	protein_coding
ENSG00000256083	9.356790141	29.12382648	-1.643003964	0.0028584	0.014083492	AC090673.2	antisense
ENSG00000217236	3.248675087	15.65063306	-2.278225469	0.002969874	0.014578738	SP9	protein_coding
ENSG00000151846	25.85203137	54.45147234	-1.070502834	0.0029752	0.014600519	PABPC3	protein_coding
ENSG00000160870	10.64042406	29.67591931	-1.48240947	0.002985866	0.014631013	CYP3A7	protein_coding
ENSG00000267780	15.01607111	2.355498606	2.660815008	0.003051904	0.014919007	AC021594.2	lincRNA
ENSG00000177989	66.03092851	31.33398934	1.074296483	0.003095363	0.015100004	ODF3B	protein_coding
ENSG00000253406	35.40358356	14.04299929	1.33472019	0.003124077	0.015212975	AC012613.2	antisense
ENSG00000254602	17.8540122	4.13741558	2.118132841	0.003189223	0.015502645	AP000662.1	sense_overlapping
ENSG00000019186	16.13077437	39.45125313	-1.2939804	0.003243181	0.015746298	CYP24A1	protein_coding
ENSG00000272430	14.58349114	2.72014809	2.415597103	0.003353994	0.016215462	AL356056.3	lincRNA
ENSG00000169403	20.71941297	46.28597655	-1.154173635	0.003359569	0.016234623	PTAFR	protein_coding
ENSG00000248569	26.9504224	59.62542286	-1.148004422	0.003373226	0.016281462	CRSP8P	processed_pseudogene
ENSG00000283689	17.9736372	4.446223053	2.015936046	0.003470968	0.016665111	AC018553.2	antisense
ENSG00000275038	13.00172428	2.058499831	2.658969956	0.003634794	0.01730503	AC091980.2	antisense
ENSG00000260088	12.41474672	32.57540213	-1.385052137	0.003641295	0.017330962	AL445483.1	lincRNA
ENSG00000178342	34.76772741	13.92000518	1.315701604	0.00368171	0.017503036	KCNG2	protein_coding
ENSG00000260997	45.61446617	20.58905262	1.151967631	0.003796166	0.01794849	AC004847.1	sense_overlapping
ENSG00000223829	48.72124322	23.54175025	1.048996282	0.003845009	0.01812726	AC004870.2	processed_transcript

ENSG00000175899	3.824932275	16.49111744	-2.100425474	0.003985089	0.018685793	A2M	protein_coding
ENSG00000255642	15.4193541	37.90749431	-1.295053105	0.004117679	0.019159861	PABPC1P4	processed_pseudogene
ENSG00000132718	5.159651023	19.12049603	-1.892611342	0.004304015	0.019874409	SYT11	protein_coding
ENSG00000266924	17.22816038	4.07296635	2.077189538	0.00468963	0.02143272	AC021594.1	lincRNA
ENSG00000167083	6.480321897	21.13496255	-1.711756179	0.005053397	0.022828517	GNGT2	protein_coding
ENSG00000220685	3.238670748	14.63884689	-2.183604138	0.005147074	0.023175233	AL139094.1	processed_pseudogene
ENSG00000111325	40.49107794	18.39845285	1.136024448	0.00525301	0.023573069	OGFOD2	protein_coding
ENSG00000244734	35.17029436	14.64204836	1.263370947	0.005287563	0.023710335	HBB	protein_coding
ENSG00000229951	12.90508836	2.411340617	2.425343978	0.005351558	0.023951538	FLJ31356	antisense
ENSG00000230291	3.735320289	17.15731861	-2.176861849	0.005581692	0.024779033	AC078817.1	processed_pseudogene
ENSG00000183484	16.27671595	36.83233179	-1.174945601	0.005629831	0.024932109	GPR132	protein_coding
ENSG00000139187	9.941561467	27.14641604	-1.446284596	0.005982224	0.026259133	KLRG1	protein_coding
ENSG00000198732	25.30834054	9.191294923	1.459379006	0.006110659	0.026744306	SMOC1	protein_coding
ENSG00000179954	16.80260435	4.43306292	1.918699435	0.006129131	0.026791021	SSC5D	protein_coding
ENSG00000188488	22.88368678	7.82531653	1.543773602	0.006152328	0.026869417	SERPINA5	protein_coding
ENSG00000168517	30.18135317	12.26563523	1.297127843	0.006193934	0.027029572	HEXIM2	protein_coding
ENSG00000257718	16.37079853	3.702412518	2.133517366	0.006272328	0.027323199	CPNE8-AS1	antisense
ENSG00000258824	22.2666962	7.191339973	1.634699566	0.006272861	0.027323199	AL122035.1	antisense
ENSG00000249839	5.811472322	24.43289096	-2.075359231	0.006427354	0.027892611	AC011330.1	unprocessed_pseudogene
ENSG00000261499	9.710478506	26.0806379	-1.428429246	0.006486806	0.02810607	AC233699.1	unprocessed_pseudogene
ENSG00000250920	12.66251086	31.53274277	-1.321155348	0.00656416	0.028381357	AC105460.1	lincRNA
ENSG00000152463	15.21934729	3.386349261	2.158716136	0.006700245	0.028870973	OLAH	protein_coding
ENSG00000186340	36.56128458	16.39579503	1.154205808	0.006897769	0.029628849	THBS2	protein_coding
ENSG00000260325	14.14721472	3.034859911	2.209784993	0.006962571	0.029868156	HSPB9	protein_coding
ENSG00000227199	29.44141009	12.01992556	1.296686514	0.007127788	0.030481405	ST7-AS1	antisense
ENSG00000267076	7.364716357	22.228911	-1.591195799	0.00741594	0.03149235	CCDC58P3	processed_pseudogene
ENSG00000165995	8.276143434	25.3422331	-1.608575554	0.007646209	0.032319839	CACNB2	protein_coding
ENSG00000228137	13.86837443	2.758277054	2.341175802	0.007727971	0.032589979	AP001469.2	antisense
ENSG00000239405	4.438226417	17.15461574	-1.940427016	0.008006612	0.033519353	TMED10P2	processed_pseudogene
ENSG00000237975	26.78346713	9.960074289	1.432193331	0.008126409	0.033912169	FLG-AS1	antisense
ENSG00000176659	5.152627086	17.38126094	-1.756564717	0.00828406	0.034440057	C20orf197	lincRNA
ENSG00000204745	66.85801022	29.52282917	1.179891952	0.008499192	0.03516538	AC083899.1	unprocessed_pseudogene
ENSG00000279569	22.72702483	8.223542064	1.469366146	0.00872391	0.035932276	AC020763.4	TEC
ENSG00000273143	5.035982485	18.75449511	-1.884529186	0.008954934	0.036699817	AL355512.1	lincRNA
ENSG00000135519	12.9121123	29.99383261	-1.219668148	0.008999352	0.036835911	KCNH3	protein_coding
ENSG00000230513	32.36790003	14.36951981	1.172991256	0.00903307	0.036955512	THAP7-AS1	antisense
ENSG00000232838	41.23804496	20.44834546	1.009254797	0.009482857	0.038536568	PET117	protein_coding
ENSG00000267281	13.32688983	3.078893224	2.110967522	0.009715586	0.039239694	AC023509.3	protein_coding
ENSG00000213760	2.65240922	12.94499654	-2.310610571	0.009874759	0.03973605	ATP6V1G2	protein_coding
ENSG00000179344	16.59485756	36.04206362	-1.113600918	0.010184742	0.04071419	HLA-DQB1	protein_coding
ENSG00000173261	13.15064626	2.693827823	2.276467717	0.011111642	0.043654778	PLAC8L1	protein_coding
ENSG00000269720	38.67857522	18.74268642	1.045079317	0.011341377	0.044429857	CCDC194	protein_coding
ENSG00000244480	15.58005959	3.715572651	2.057411389	0.011445558	0.044805937	AC005154.2	transcribed_processed_pseudogene

ENSG00000258654	16.68446954	4.739167521	1.809279536	0.011534758	0.045047796	AC026495.1	lincRNA
ENSG00000165887	26.61900707	10.86608079	1.289000039	0.011559715	0.04509167	ANKRD2	protein_coding
ENSG00000255031	36.61463377	17.37941089	1.073353605	0.011569495	0.04510094	AP002807.1	antisense
ENSG00000215861	6.739927674	19.91964997	-1.561010097	0.012022735	0.046534537	AC245297.1	unprocessed_pseudogene
ENSG00000144837	15.48342367	4.412647002	1.804511459	0.012023074	0.046534537	PLA1A	protein_coding
ENSG00000039139	10.14305848	26.02344445	-1.35078498	0.012104872	0.046763002	DNAH5	protein_coding
ENSG00000258727	14.75901866	3.783223359	1.968521854	0.012198908	0.047059887	AL135999.1	antisense
ENSG00000180878	20.3579265	7.153211009	1.504736773	0.012312168	0.047429994	C11orf42	protein_coding
ENSG00000165874	40.49881792	18.27118437	1.150350483	0.012353243	0.047565919	SHLD2P1	transcribed_unprocessed_pseudogene
ENSG00000101280	14.86161539	32.21665699	-1.118314129	0.012543364	0.048207594	ANGPT4	protein_coding
ENSG00000115155	15.78453701	4.496160715	1.819259507	0.01277395	0.0490021	OTOF	protein_coding
ENSG00000134259	11.2537182	26.90448212	-1.258946424	0.01278092	0.04900916	NGF	protein_coding
ENSG00000112902	16.36781813	5.150553188	1.671658117	0.01287825	0.049272166	SEMA5A	protein_coding
ENSG00000182459	15.7074827	4.077519263	1.942013809	0.012979775	0.049594816	TEX19	protein_coding
ENSG00000110944	18.95062405	38.45582856	-1.022184443	0.012985952	0.049606878	IL23A	protein_coding
