# Supplementary material for: Deubiquitinase USP7 stabilizes KDM5B and promotes tumor progression and cisplatin resistance in nasopharyngeal carcinoma through the ZBTB16/TOP2A axis
Source: Cell Death Differ. 2024 Jan 29;31(3):309–21. doi: 10.1038/s41418-024-01257-x (PMC10923876; doi:10.1038/s41418-024-01257-x)

Figure 1 e

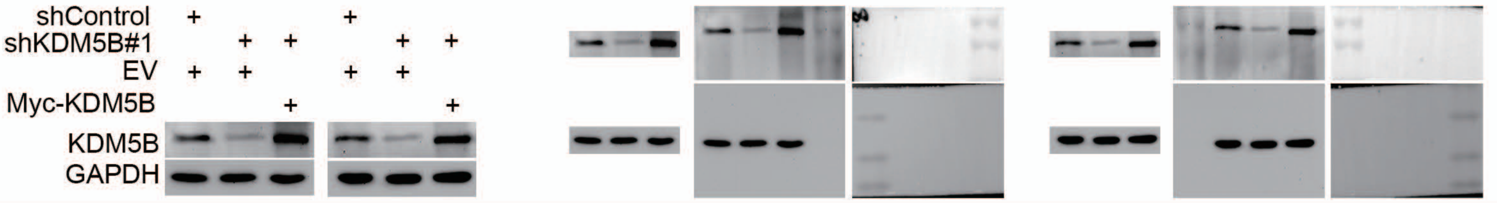

Figure 2 g

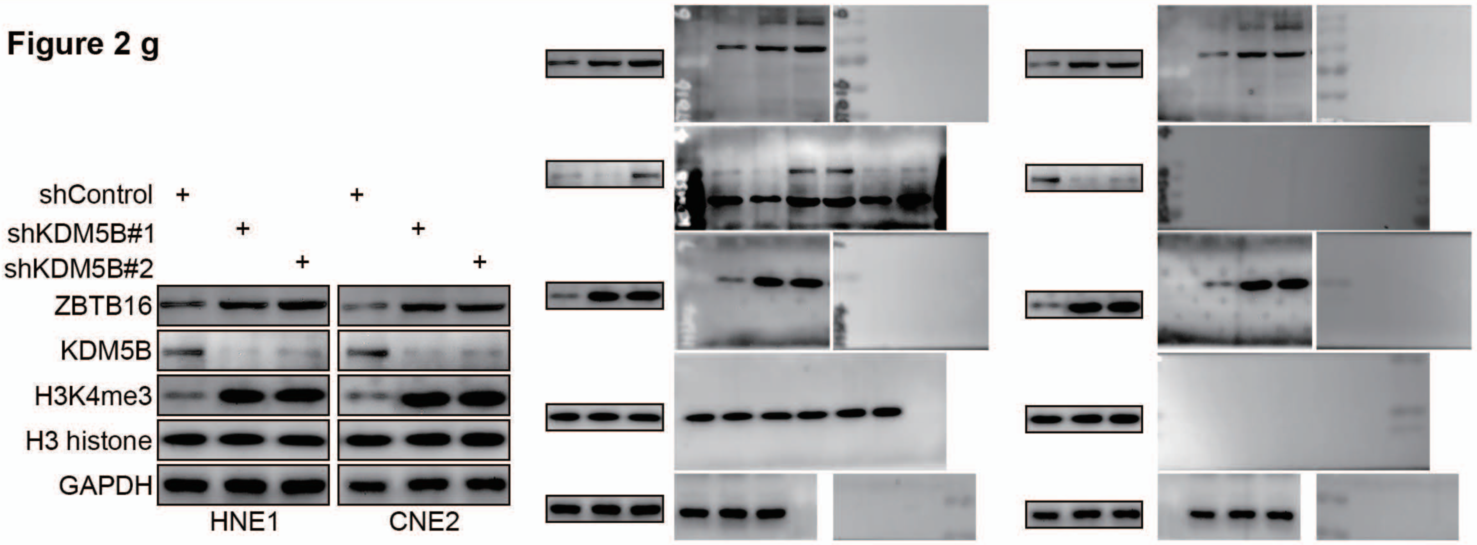

Figure 2 h

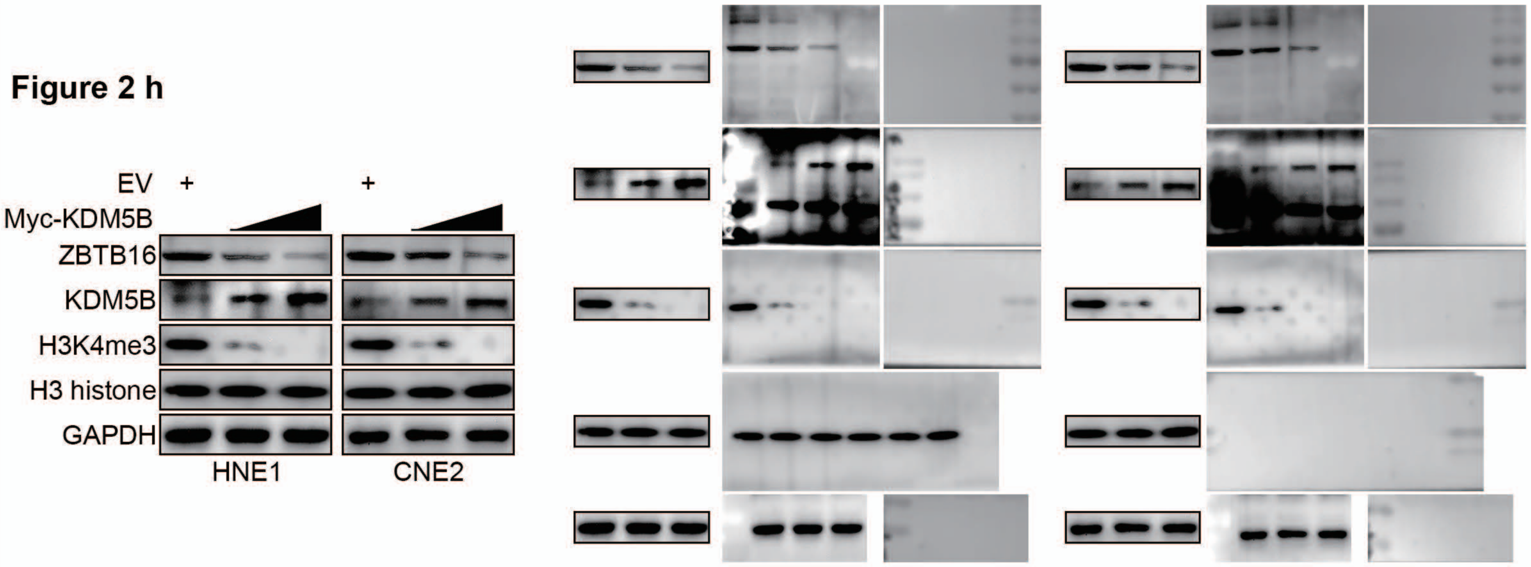

Figure 3 a

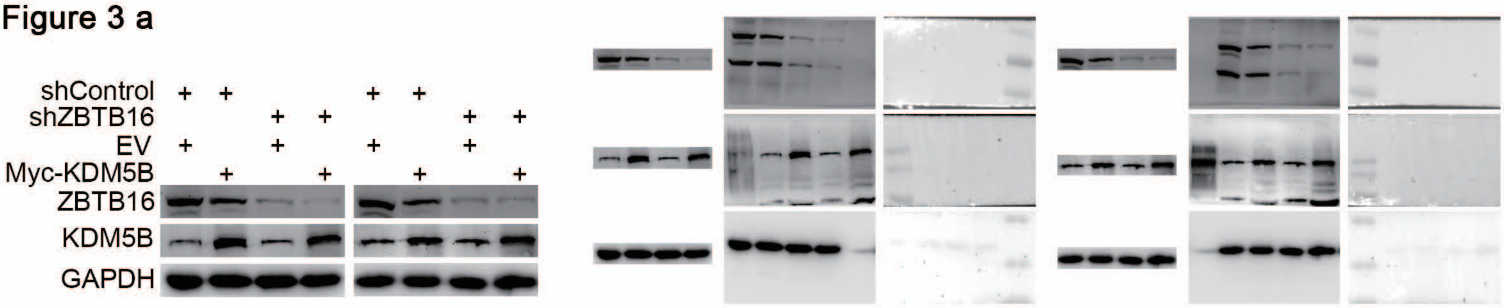

Figure 3 c

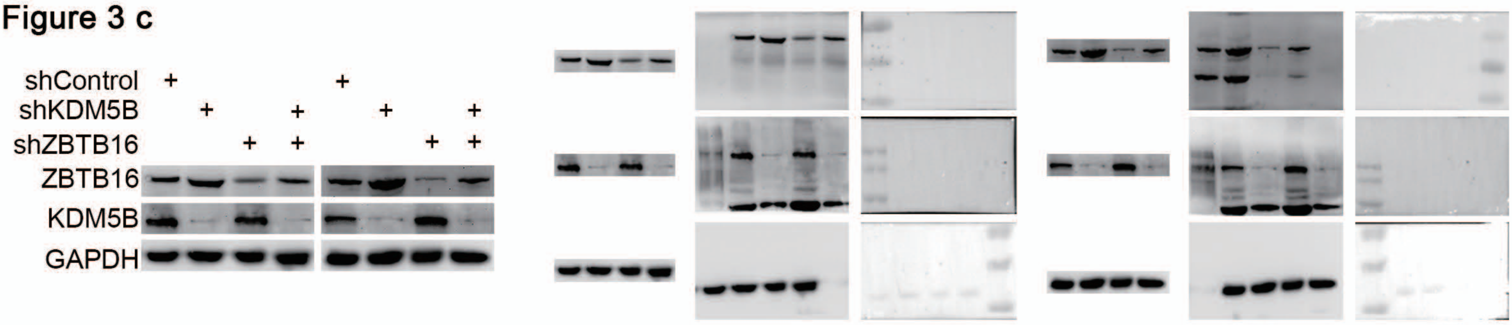

Figure 4 g

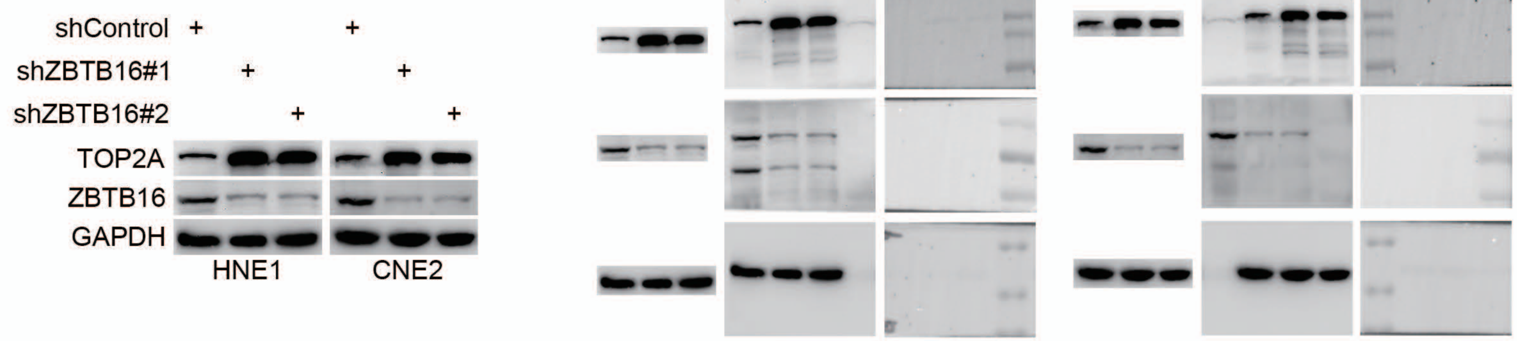

Figure 4 h

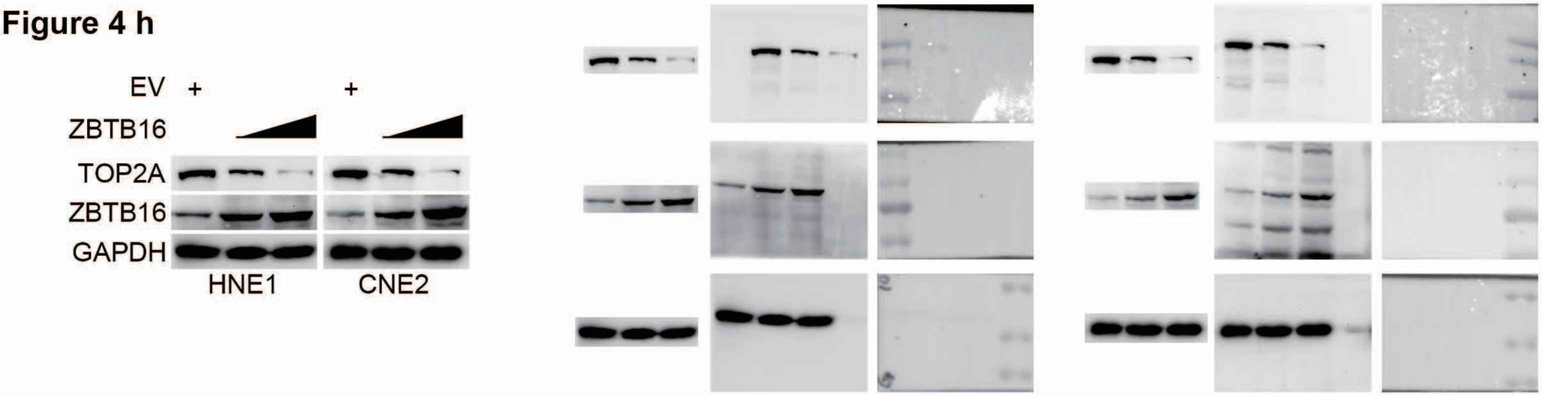

Figure 5 a

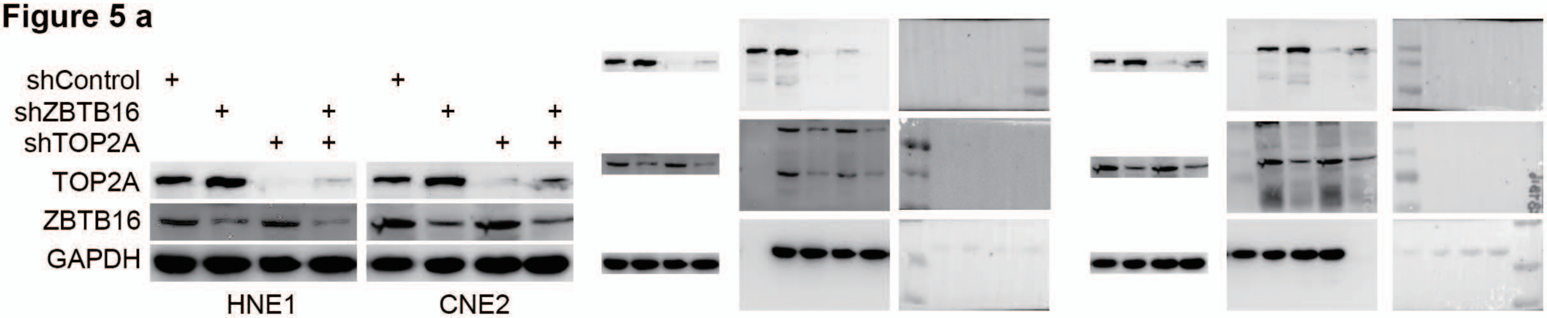

Figure 5 g

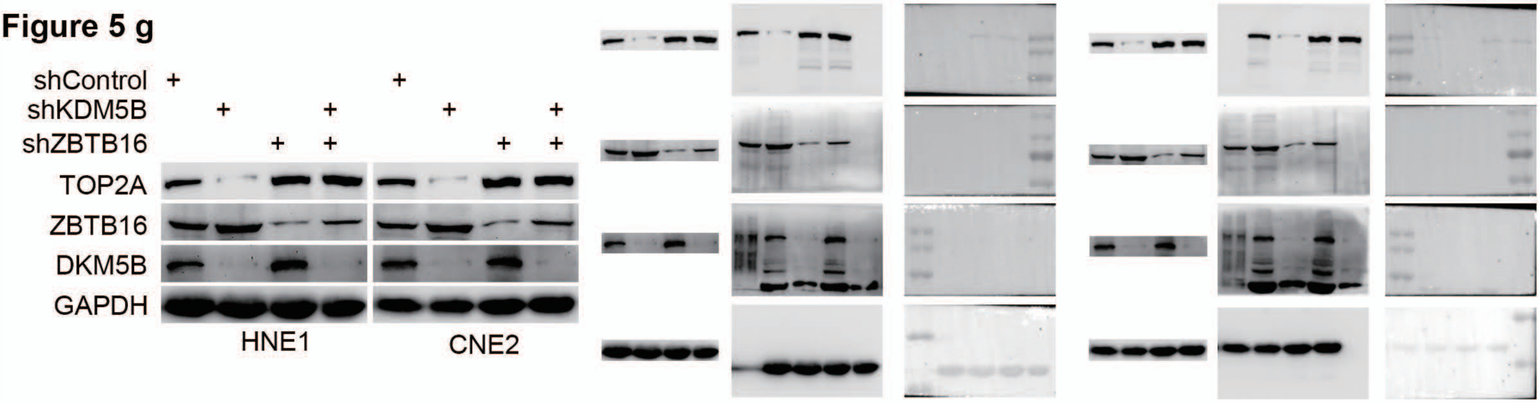

Figure 6 c

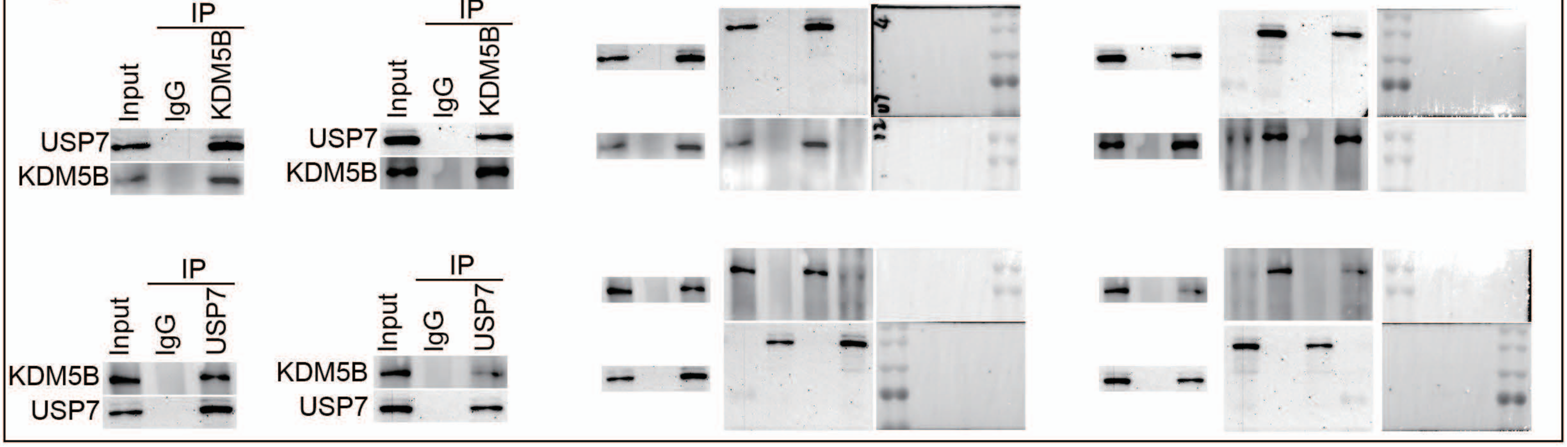

Figure 6 d

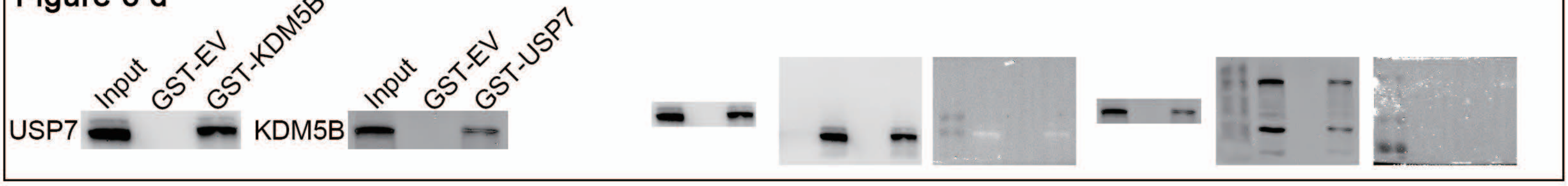

Figure 6 e

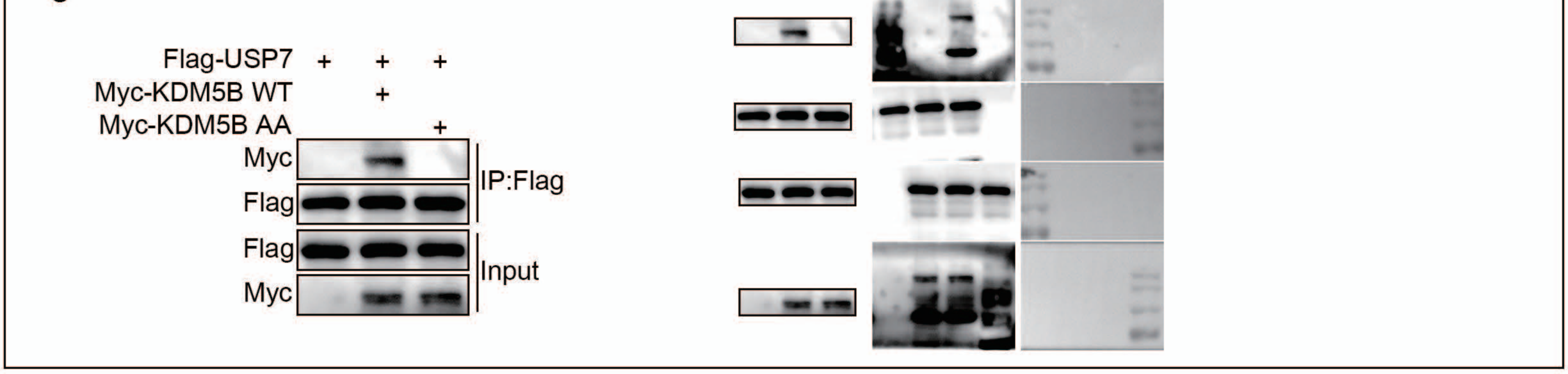

Figure 6 f

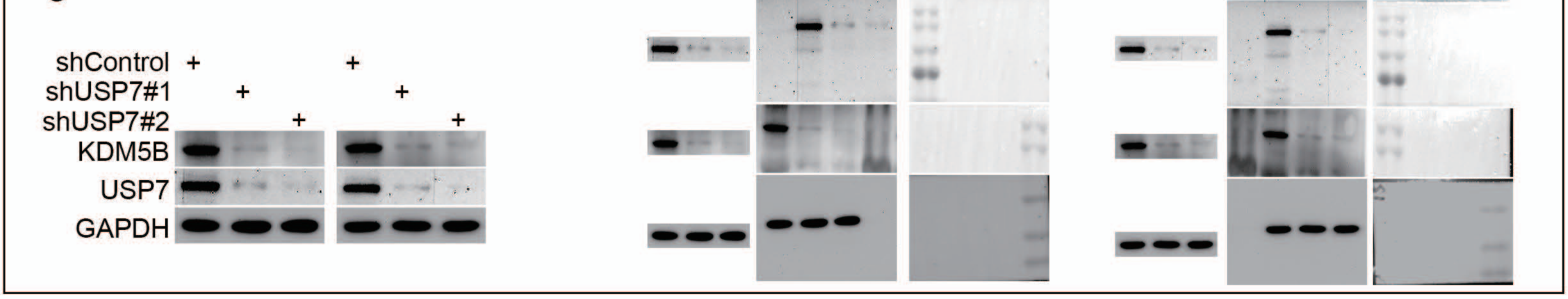

Figure 6 g

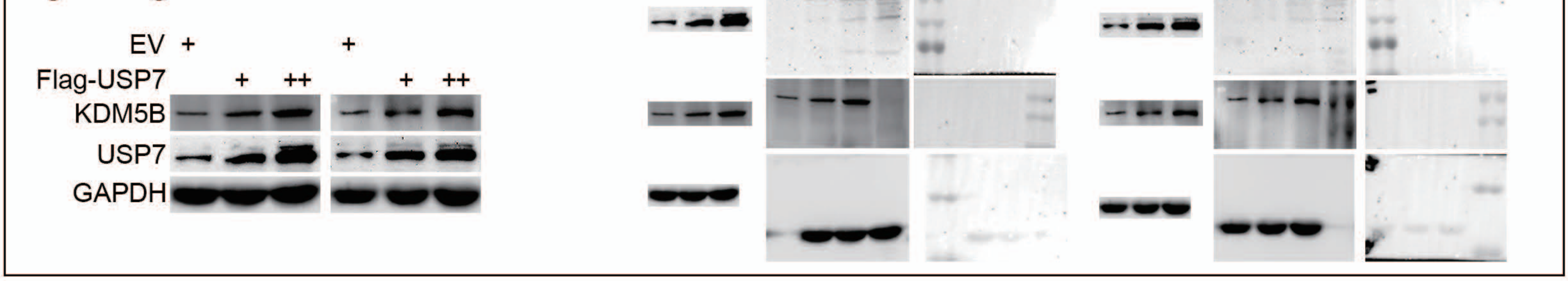

Figure 6 h

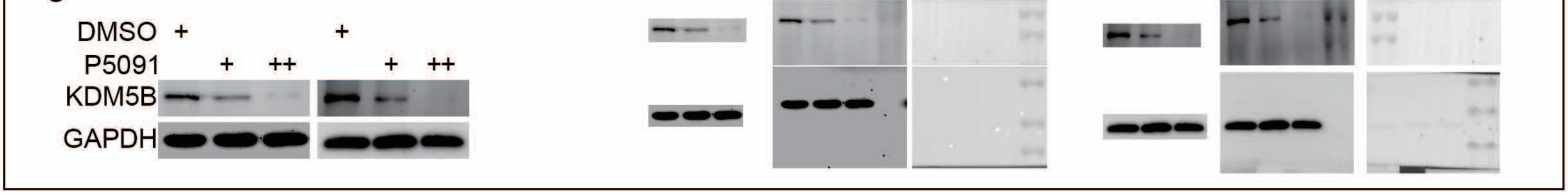

Figure 6 i

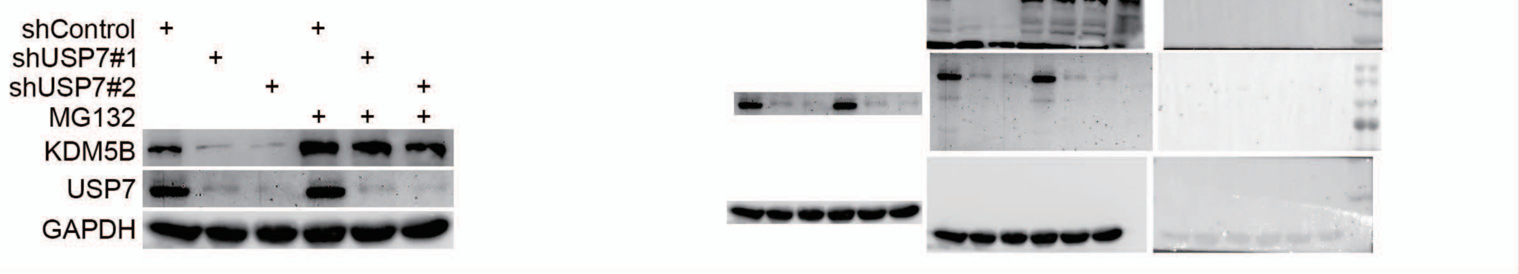

Figure 6 j

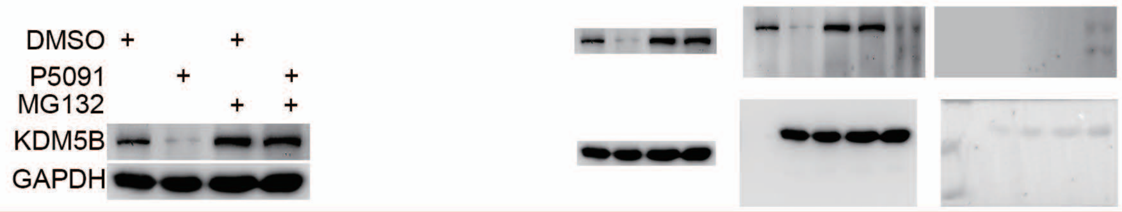

Figure 6 k

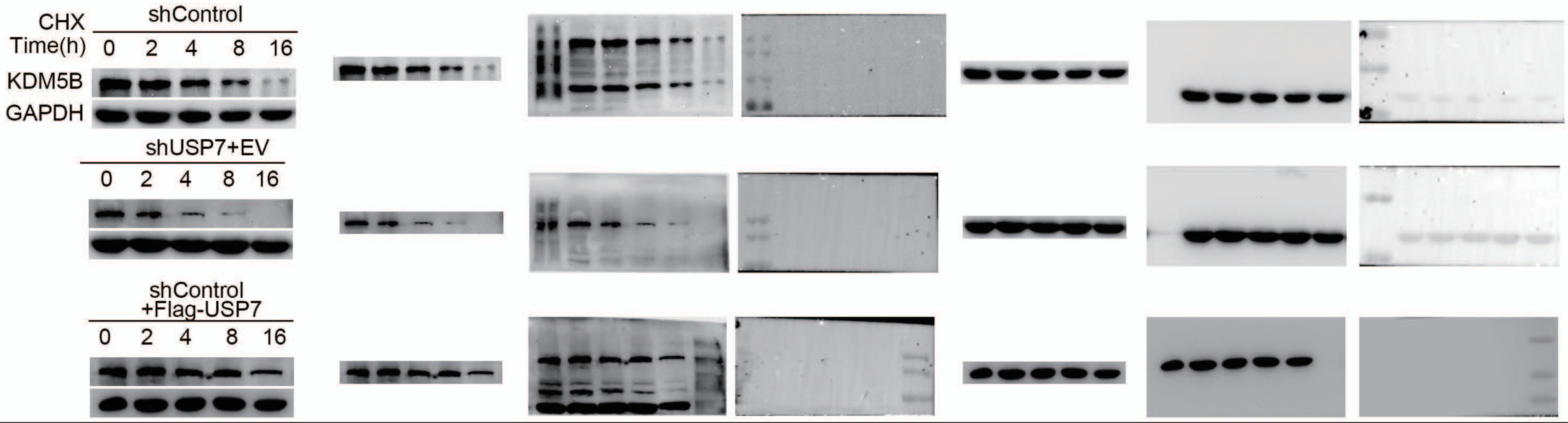

Figure 6 l

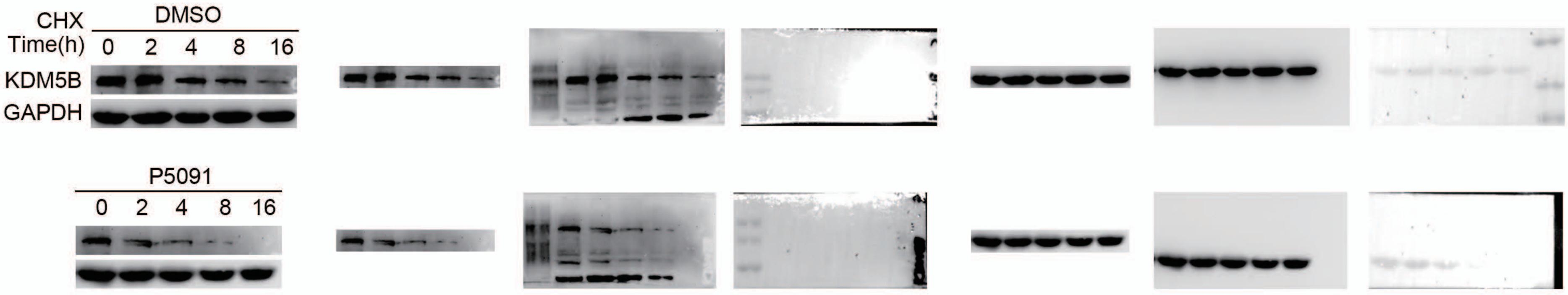

Figure 6 n

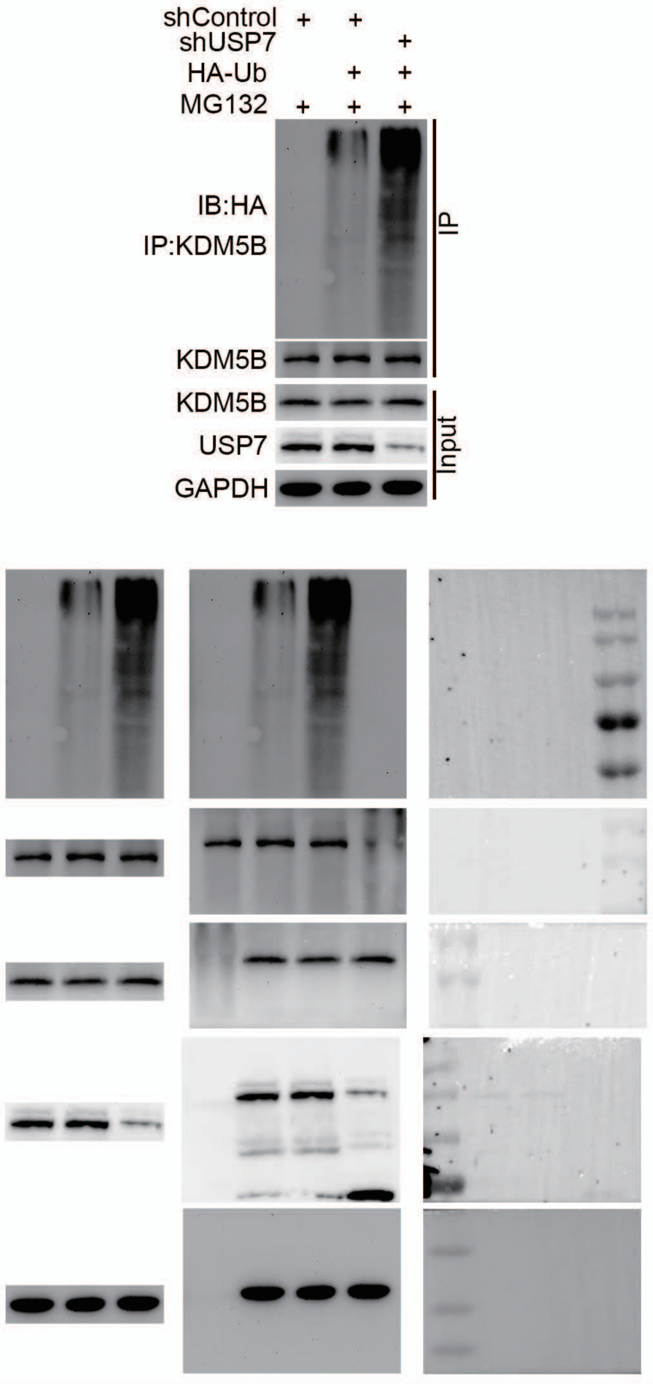

Figure 6 o

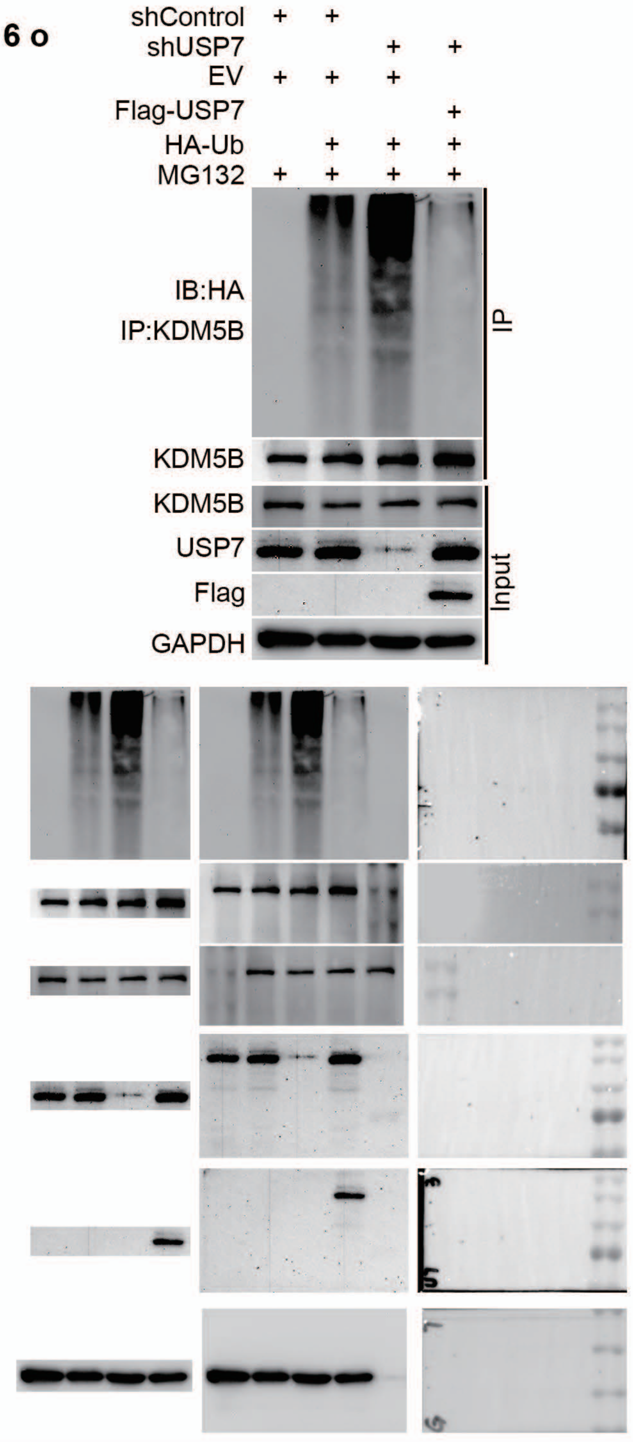

Figure 7 a

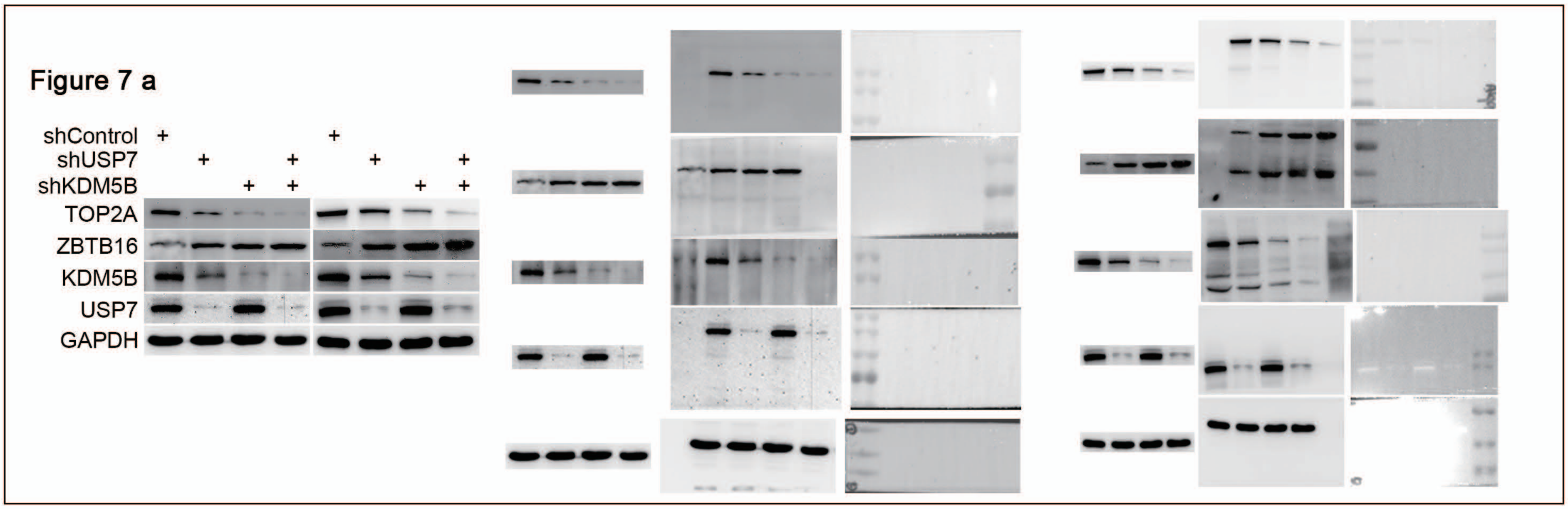

Figure 7 b

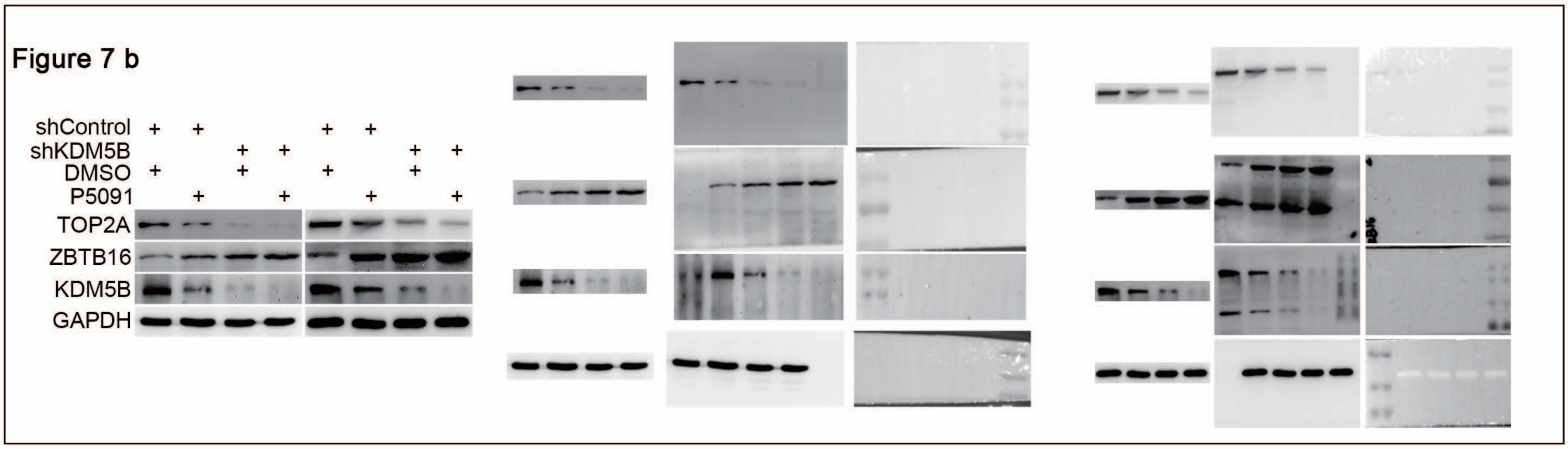

Figure 7 c

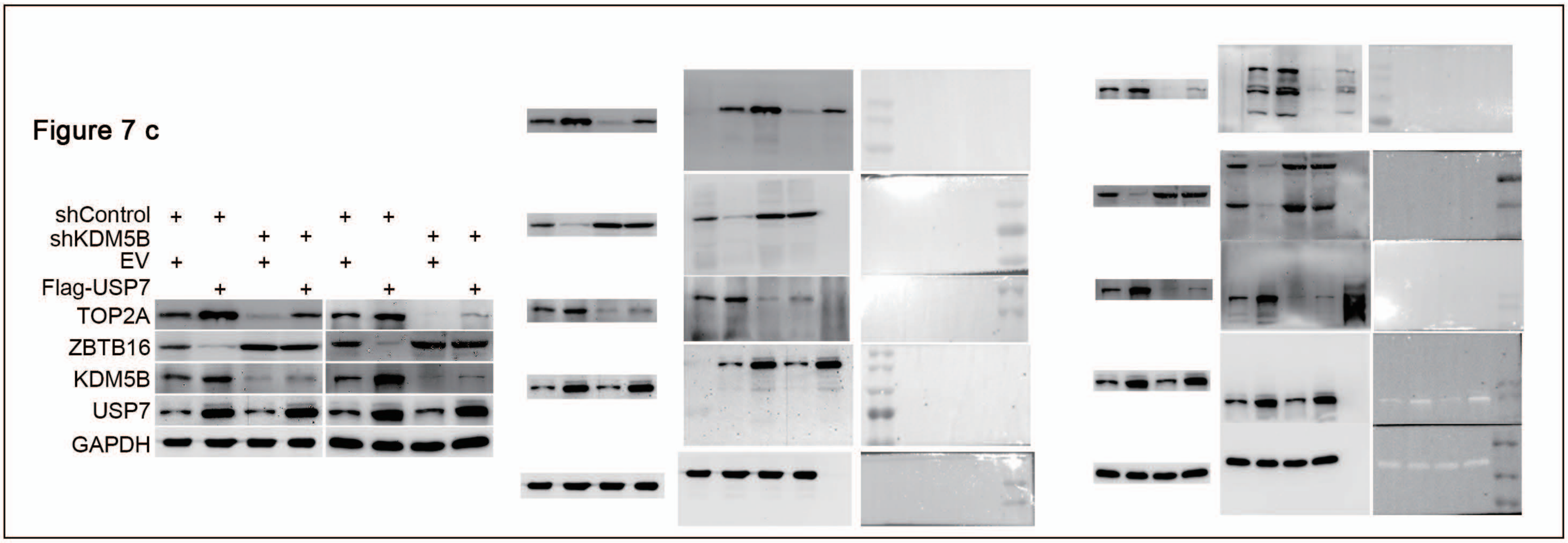

Figure 7 d

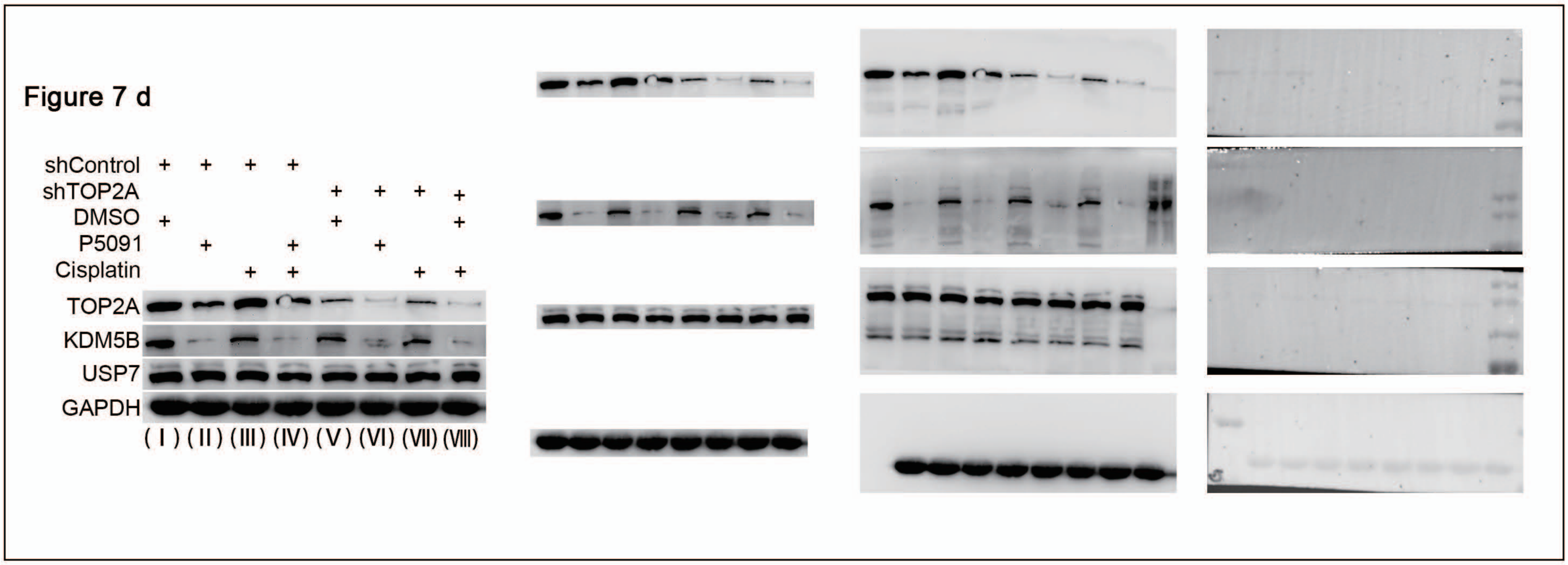

Supplementary Figure 2 a

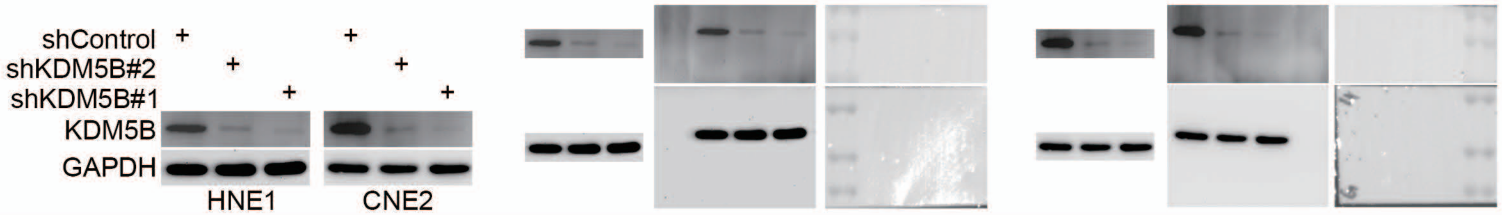

Supplementary Figure 2 i

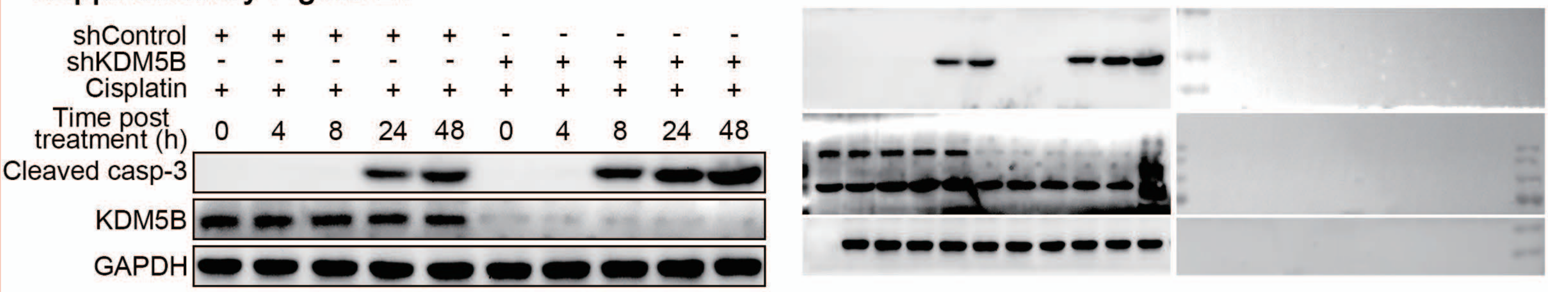

Supplementary Figure 3 a

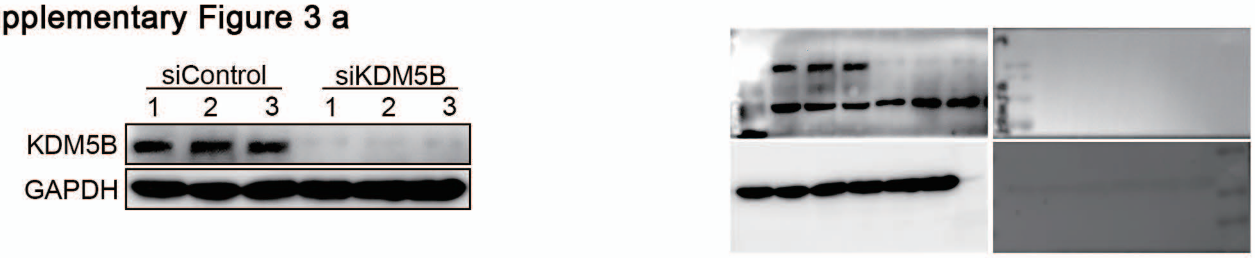

Supplementary Figure 4 a

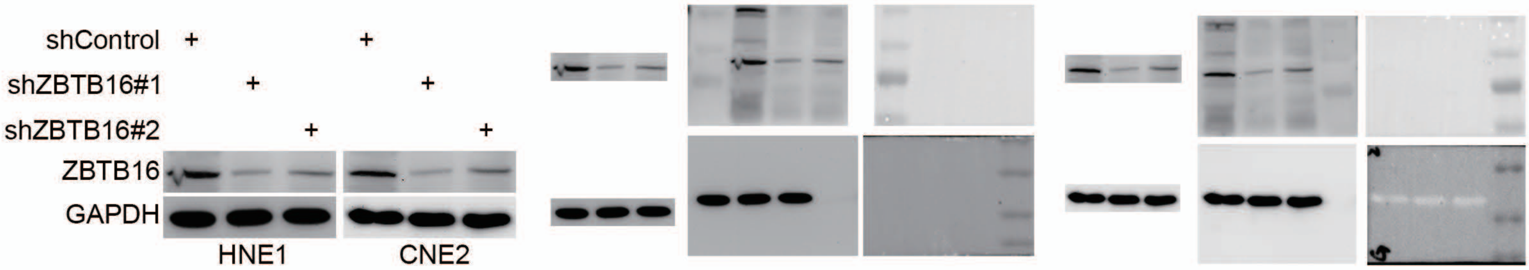

Supplementary Figure 5 c

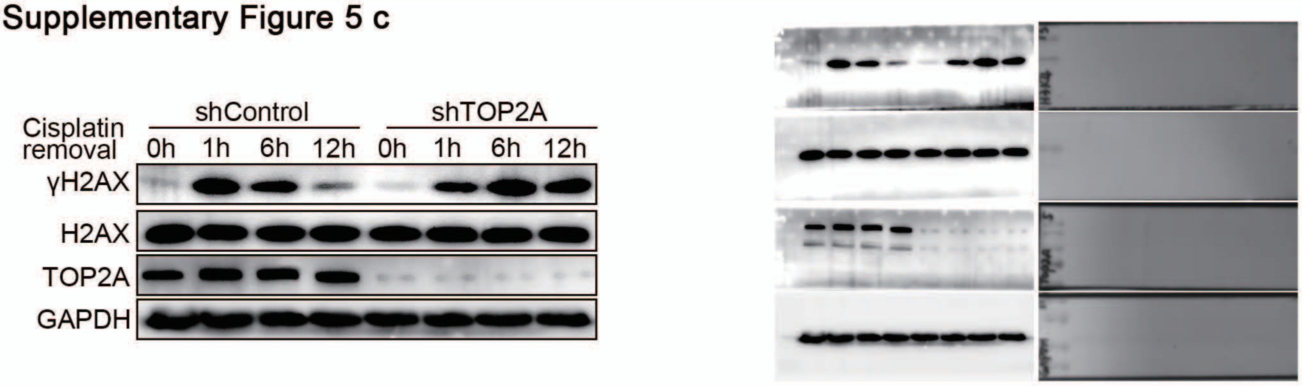

Supplementary Figure 5 f

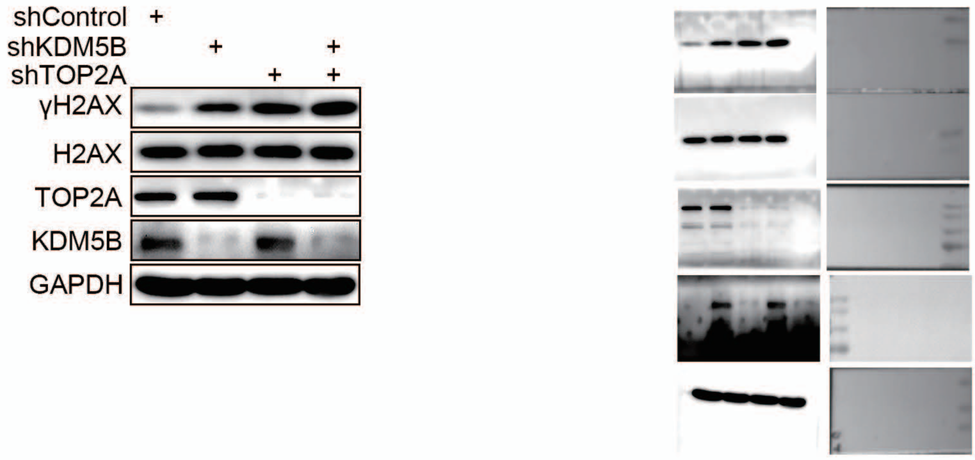

Supplementary Figure 6 a

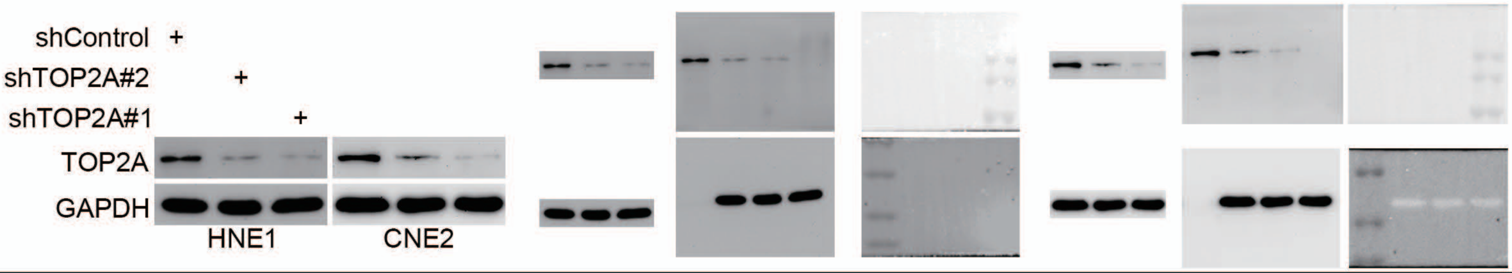

Supplementary Figure 8 a

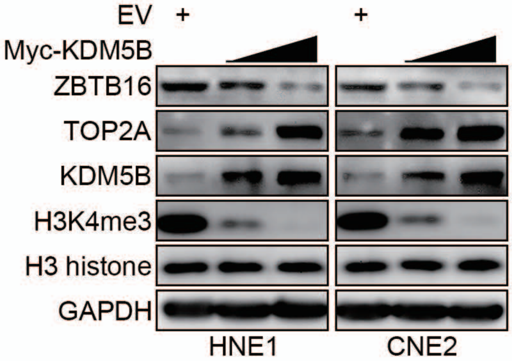

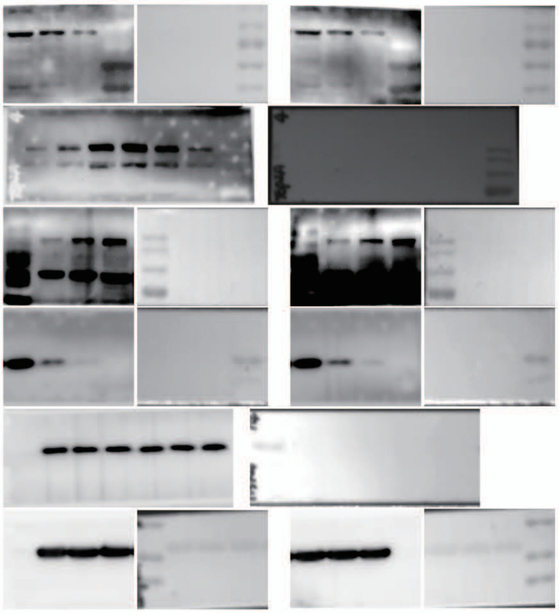

Supplementary Figure 8 c

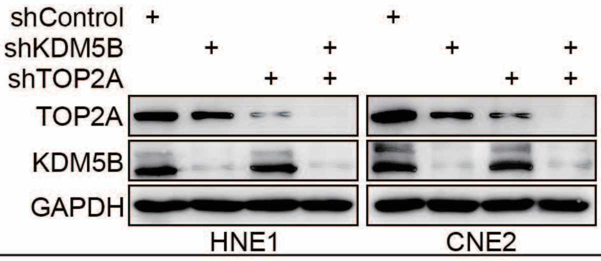

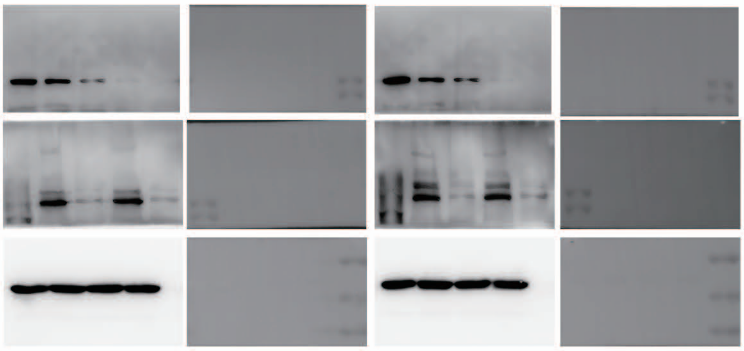

Supplementary Figure 8 e

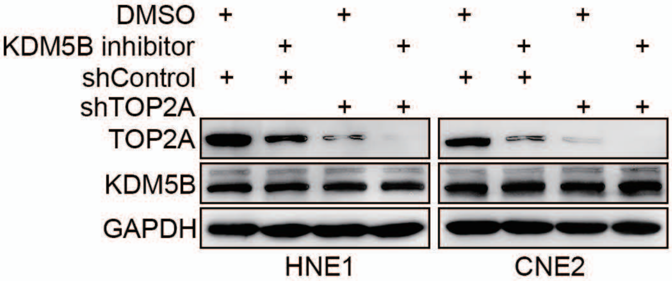

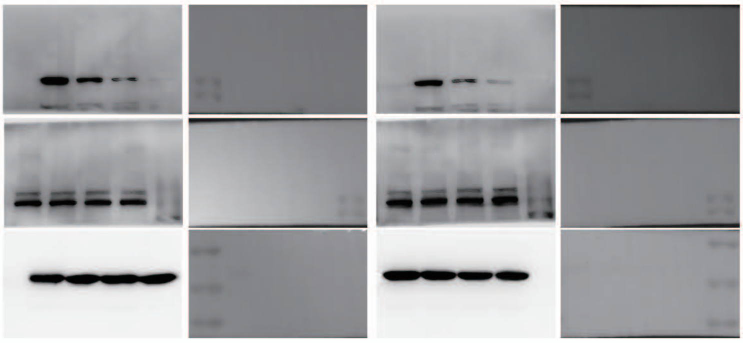

Supplementary Figure 8 g

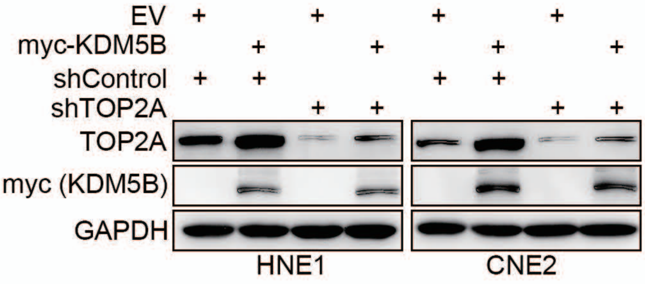

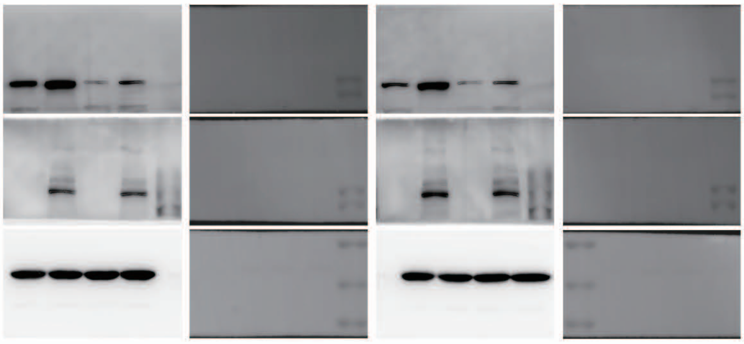

Supplement: Supplementary file 2 — Original Data File [file 41418_2024_1257_MOESM2_ESM.pdf]
